# 3D Macrocyclic Structure Boosted Gene Delivery: Multi-Cyclic Poly(β-Amino Ester)s from Step Growth Polymerization

**DOI:** 10.1021/jacs.3c04191

**Published:** 2023-07-25

**Authors:** Yinghao Li, Xianqing Wang, Zhonglei He, Melissa Johnson, Sigen A, Irene Lara-Sáez, Jing Lyu, Wenxin Wang

**Affiliations:** † Research and Clinical Translation Center of Gene Medicine and Tissue Engineering, School of Public Health, Anhui University of Science and Technology, Huainan 232001, China; ‡ Charles Institute of Dermatology, School of Medicine, University College Dublin, Dublin 4, Dublin D04V1W8, Ireland; § School of Medicine, Anhui University of Science and Technology, Huainan 232001, China

## Abstract

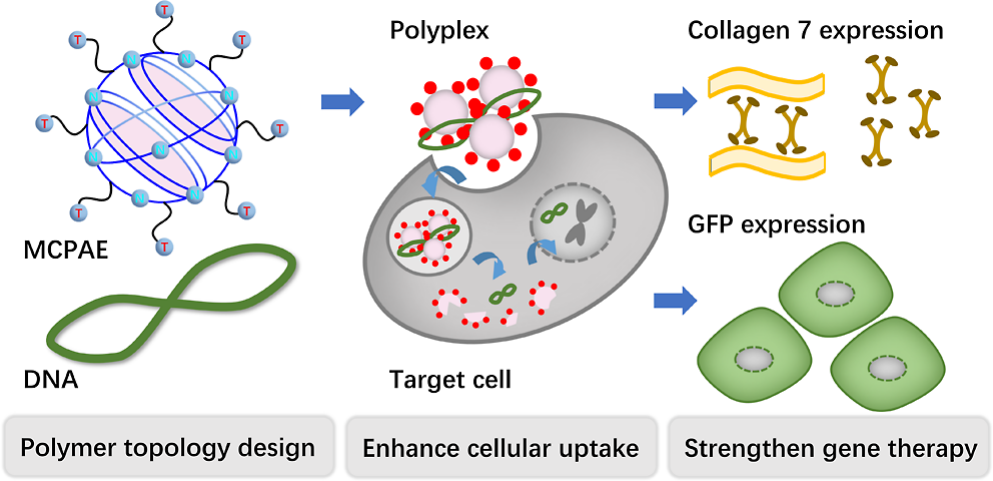

The topological structures of polymers play a critical role in determining their gene delivery efficiency. Exploring novel polymeric structures as gene delivery vectors is thus of great interest. In this work, a new generation of multi-cyclic poly(β-amino ester)s (CPAEs) with unique topology structure was synthesized for the first time via step growth polymerization. Through controlling the occurrence stage of cyclization, three types of CPAEs with rings of different sizes and topologies were obtained. In vitro experiments demonstrated that the CPAEs with macro rings (MCPAEs) significantly boosted the transgene expression comparing to their branched counterparts. Moreover, the MCPAE vector with optimized terminal group efficiently delivered the CRISPR plasmid coding both *Staphylococcus aureus* Cas9 nuclease and dual guide sgRNAs for gene editing therapy.

## Introduction

Gene therapy has become an essential field in medical research owing to its prospects for treatment for numerous diseases.^1,2^ According to the market research from Emergent Research, international cell and gene therapy companies could generate $6.6 billion in revenue by 2027, with a projected compound annual growth rate (CAGR) of 19.8% from 2020 to 2027. However, the lack of safe and efficient gene delivery vectors remains a major obstacle to large-scale clinical applications. Although viral vectors are highly effective vehicles, significant safety concerns such as severe immune responses, activation of viral components, and limited insert size have compromised their application.^3,4^ As for liposome vectors, the lipoplexes suffer from poor colloidal stability in physical environments and an inflammation potential.^5^ In contrast, polymeric non-viral vectors offer several advantages, including easy operation, modification, and purification, inexpensive synthesis and scalability, high transfection efficiency, and low cytotoxicity. These features make the polymeric vectors highly promising candidates for future gene therapy applications.^6^

Poly(β-amino ester)s (PAEs) are one of the most versatile polymer vectors for gene delivery. Since the first report by Lynn and Langer in 2000, more than 2500 linear PAEs (LPAEs) have been synthesized and tested in gene delivery.^7,8^ Although the results from LPAEs have been encouraging, the linear nature of these polymers inherently limits the potential for optimizing structures. In 2016, Wang et al. constructed highly branched PAEs (HPAEs) via a facile A2 + B3 + C2 Michael addition strategy.^9,10^ Their results have shown that the transition from linear to branch structure introduced numerous terminal functional groups, which enhanced the interaction with DNA and optimized the polymer’s assembly behavior, thus the gene transfection performance of HPAEs was significantly improved compared to their linear counterparts.^9,11−14^ This discovery has clearly demonstrated that the macromolecular structure of polymer vectors has a significant effect on their transfection efficacy. Therefore, further harnessing the polymer topology is promising for the development of next-generation highly efficient polymer gene delivery vectors.

Among the various topologies of synthetic polymers, including linear, branched, dendritic, star, and cyclic structures, cyclized polymers are of significant interest due to their 3D compact architectures and unique properties. Previous research has demonstrated that polymers with a cyclic structure often display better biocompatibility and DNA encapsulation capabilities than those with a linear or branched structure.^12,14,15^

Herein, we report the first successful construction of a series of cyclic poly(β-amino ester)s (CPAEs) with different types of ring structures by regulating the cyclization tendency at different stages of the step-growth polymerization (SGP). Their gene delivery performance and the mechanism behind the enhanced transfection were investigated and illustrated by comparing with the corresponding HPAE vectors. Finally, a class of optimal CPAE polymers with macro rings were generated after screening and optimizing the terminal groups. They were applied to efficiently deliver a CRISPR plasmid that codes for both *Staphylococcus aureus* Cas9 nuclease and dual guide sgRNAs for gene editing therapy.

## Results and Discussion

### SGP Strategy towards the Synthesis of CPAEs with Rings of Different Sizes

**Scheme 1:**
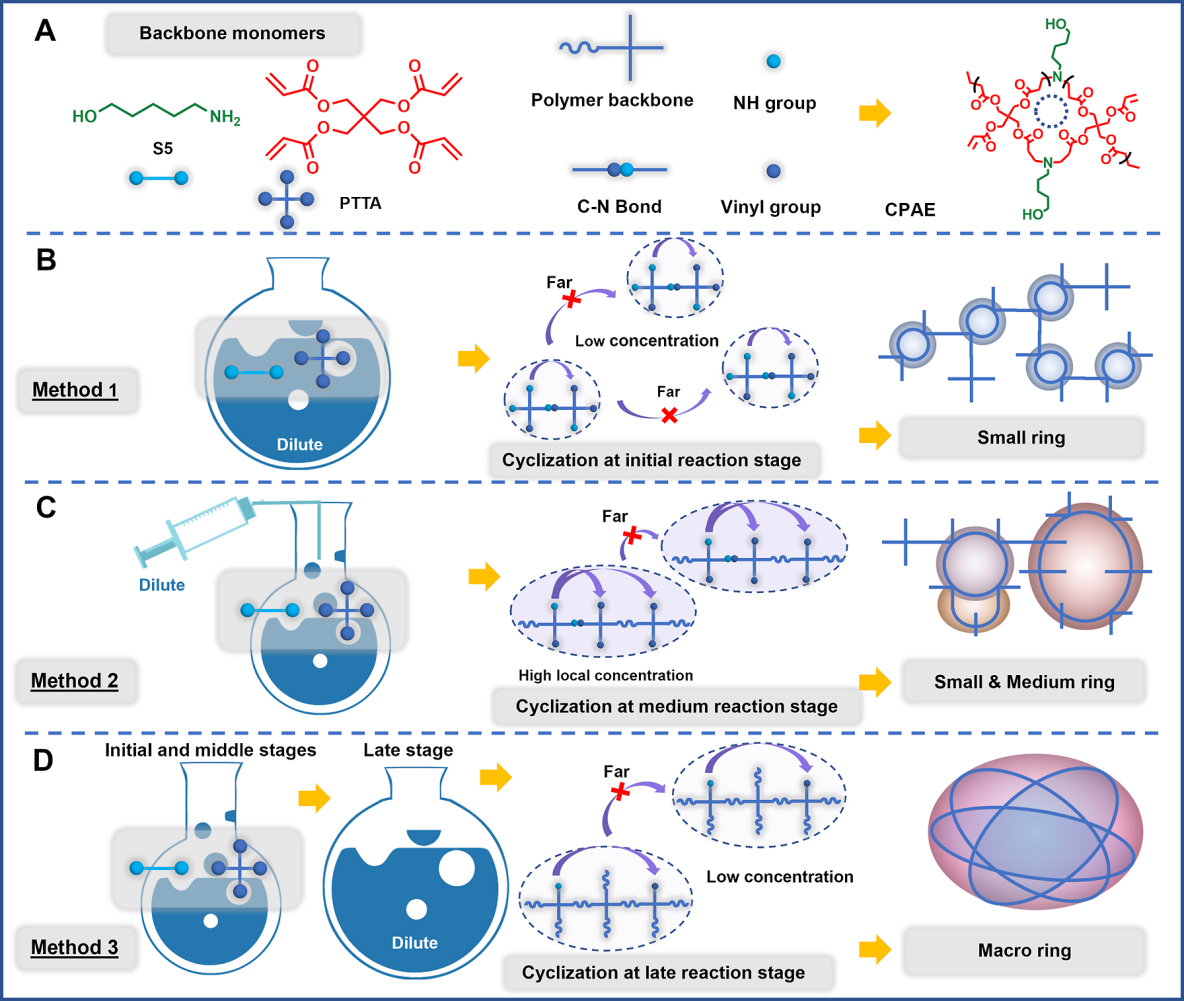
Schematic Illustration of the Chemical Structure and Formation Mechanism of Three Types of CPAEs Generated from Different SGP Methods (A) Structure of the monomer 5-amino-1-pentanol (S5) and pentaerythritol tetra acrylate (PTTA) used for CPAE synthesis. (B) Formation mechanism of CPAE with small primary rings via Method 1—direct dilution strategy. Cyclization occurred at the initial SGP reaction stage. (C) Formation mechanism of CPAE with small and medium rings via Method 2—continuous dilution strategy. Cyclization occurred at the middle SGP stage along with the chain propagation. (D) Formation mechanism of CPAE with macro rings via Method 3—late dilution strategy. Cyclization occurred at the late SGP stage between functional groups far apart on high MW macromolecular chains in diluted condition, resulting in macro rings.

In SGP, the generation of cyclic structure depends on the significant enhancement of cyclization probability relative to that of chain growth (i.e., interchain combination).^16,17^ To achieve this, the most straightforward strategy is to dilute the reaction system, which suppresses the accessibility among different chains while promoting the reaction within a molecular chain itself. Moreover, diluting the reaction system at different SGP stages leads to different cyclization structures. With these insights, in this work, using the well-studied pentaerythritol tetraacrylate (PTTA) and 5-amino-1-pentanol (S5) as backbone monomers (Scheme 1A), three SGP strategies were designed to regulate the ring-forming kinetics towards the synthesis of CPAEs with three different types of ring structures (Scheme 1B–D).

**Figure 1:**
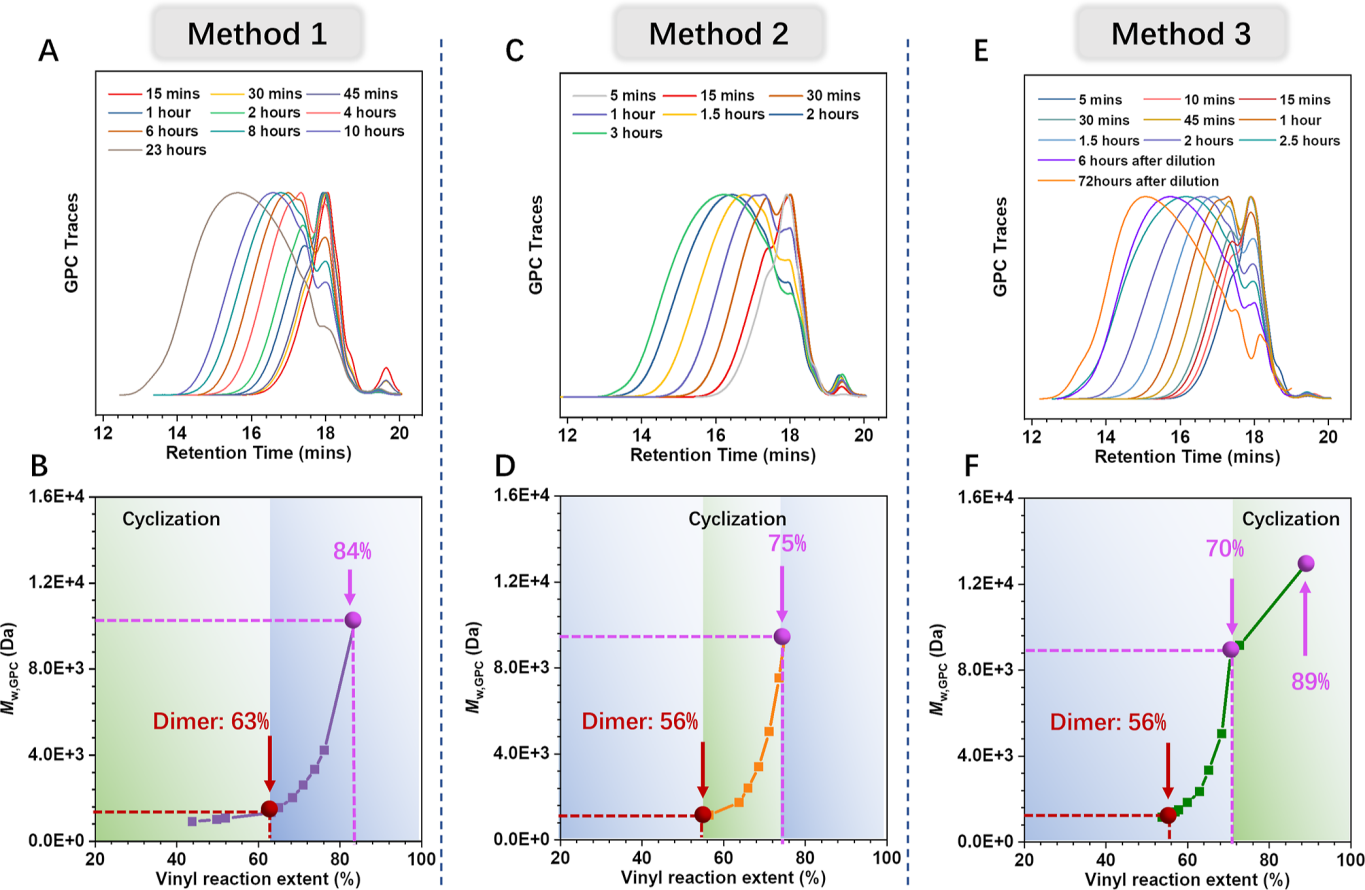
Comparison of the polymerization behaviors during the formation of three types of CPAEs via three different SGP strategies. Evolution of GPC traces of CPAE-1 (A), CPAE-2 (C), and CPAE-3 (E). Changes of weight-average molecular weight (*M*_w,GPC_) with vinyl reaction extent during the (B) Method 1 (CPAE-1), (D) Method 2 (CPAE-2), and (F) Method 3 (CPAE-3) processes. The red dash line refers to the dimer MW (*M*_w,GPC_ ∼ 1300 Da). The purple dash line refers to the polymer with targeted MW before endcapping (*M*_w,GPC_ ∼ 9500 Da). The green area is the cyclization active zone. The blue area represents the propagation dominant zone. The vinyl reaction extent was calculated via ^1^H Nuclear Magnetic Resonance (^1^H NMR) (Figures S1–S3).

Method 1—Direct dilution strategy (cyclization occurred at the initial SGP stage, Scheme 1B). Previous studies by Kricheldorf and Schwarz have shown that polymers obtained under dilution conditions contain a large number of structural units consisting of cyclic and bicyclic oligomers.^17^ Therefore, to obtain CPAEs consisting of small rings (termed as SCPAE), polymerization was carried out directly in a low reaction concentration (100 mg/mL—a typical dilute concentration that has been used in the endcapping step in HPAE synthesis to decrease the trend of intermolecular combinations.^9,18^) to promote the occurrence of primary cyclization at the beginning of the reaction. The progress of this cyclization process is shown in Figure 1A,B. It can be seen that 63% of vinyl groups have been consumed when the polymer molecular weight (MW) was close to dimer (*M*_w,GPC_ ∼ 1300 Da, GPC trace of 1 h in Figure 1A, red dash line in Figures 1B and S1). This reaction extent is much higher than the theoretical vinyl reaction extent of dimers assuming no cyclization reactions—44% (calculated from Stockmayer’s equation^19^) and that of the other two methods (56% for Method 2 and Method 3, Figure 1C,F), indicating that in Method 1, more vinyl groups were consumed by cyclization at the early stage (due to the steric hindrance of the single PTTA molecule, cyclization could only happen after a few monomers were combined). After this stage, due to the rigidity of these small primary rings, the accessibility of functional groups on the same oligomer was limited, thereby intermolecular combination was promoted to form macromolecules. When reacted to the targeted MW (*M*_w,GPC_∼ 10,311 Da, 84% vinyl reaction extent, Figure 1B), amine-rich monomer 1-(3-aminopropyl)-4-methylpiperazine (E7) was added to endcap unreacted vinyl groups to generate the final SCPAE-CPAE-1 (23 h in Figure 1A, entry 1 in Table 1, Figures S4–S6).

**Table 1:** GPC and ^1^H NMR Characterization Results of CPAEs and HPAEs

entry	polymer	*M*_w,GPC_ (Da)	terminal ratio (TR)^c^
1	CPAE-1^a^	11,651	0.628
2	CPAE-2^a^	11,609	0.839
3	CPAE-3^a^	14,244	0.662
4	HPAE-1^b^	11,854	1.404
5	HPAE-2^b^	11,544	1.166

a CPAE-1, CPAE-2 and CPAE-3 were synthesized by Method 1, Method 2, and Method 3, respectively.

b HPAE-1 and HPAE-2 was synthesized via the classic SGP method^9^ using the same monomers PTTA, S5, and E7.

c Terminal ratio (TR) is the molar ratio of E7/PTTA, calculated by ^1^H NMR (Figures S5, S8, S11, S14 and S17).

Method 2—Continuous dilution strategy (cyclization occurred at the middle SGP stage, Scheme 1C). In this method, the reaction system was continuously diluted by DMSO using a metering pump (100 μL/min). Thus, at the initial stage, where the reaction concentration was still relatively high (300 mg/mL), intermolecular reactions dominated. Then, with the continuous addition of DMSO to the reaction mixture, the probability of cyclization was gradually enhanced. Thus, along with the growth of polymer chains, CPAEs with rings of different sizes (progressively larger as the polymerization proceeded) were obtained (termed as SMCPAE). The above polymerization processes can be reflected from Figure 1D. Since the PAE dimers were formed (56% vinyl reaction extent and *M*_w,GPC_ ∼ 1300 Da), approximately 19% of vinyl groups were consumed until the targeted MW (*M*_w,GPC_ ∼ 9481 Da) was reached (Figure S2). This value is higher than that in Method 3 (14%, Figure 1F), indicating the favorable occurrence of intramolecular cyclization in Method 2 at the middle SGP stage. In addition, the formation of macro rings (which are dominant in Method 3 as described in the following text) at the late stage was suppressed due to the significant steric hindrance of the multi-cyclized chain structure formed along with the molecule propagation. After endcapping, the final SMCPAE polymer CPAE-2 consisting of small and medium rings was obtained (3 h in Figure 1C, entry 2 in Table 1, Figures S7–S9).

Method 3—Late dilution strategy (cyclization occurred at the late SGP stage, Scheme 1D). In this method, the reaction system was diluted at the late stage approaching the targeted MW (i.e., *M*_w,GPC_ = 8801 Da at 2.5 h in Figures 1E and 70% vinyl reaction extent in Figure 1F). Therefore, the classic branched PAE structure was allowed to be generated first. Then, after diluting the reaction system, the tendency to intermolecular reactions was suppressed, while intramolecular cyclization was promoted. The cyclization reaction predominantly occurred at this stage, as reflected by the slower increasing rate of MWs compared to that before dilution (Figure 1F), that is, *M*_w,GPC_ increased from 8801 to 12,965 Da when 19% vinyl groups were consumed (Figure S3). Moreover, at this stage, the vinyl reaction extent and MW were already relatively high, the functional groups that were available for further cyclization reactions were less than 10%, and were separated by a large number of monomer units on the high MW polymer backbone. This significant steric hindrance made the adjacent functional groups difficult to access each other; therefore, only two functional groups far apart were able to contact and react with each other through conformational changes resulting in macro rings (termed as MCPAE). *Via* this method, CPAE-3 was obtained for the following characterization (2.5 h in Figure 1E, entry 3 in Table 1, Figures S10–S12).

### Structure Characterization of CPAE-1, CPAE-2, and CPAE-3

To validate the different cyclic structures of CPAEs synthesized using different methods, CPAE-1 (Method 1), CPAE-2 (Method 2), and CPAE-3 (Method 3) were thoroughly characterized. HPAE-1 with the same chemical composition (i.e., PTTA, S5 and E7 units, Figure S14) and similar *M*_w,GPC_ (entry 4 in Table 1 and Figure S13) was also prepared for comparison (see detailed polymerization procedure in Supporting Information).^9^ Then, the terminal ratios (TRs, the molar ratio of E7/PTTA)^20−22^ were calculated for CPAE-1, CPAE-2, CPAE-3, and HPAE-1 based on their ^1^H NMR spectra (Figures S5, S8, S11 and S14), and the results were listed in Table 1. It can be clearly seen that compared to the terminal ratio of HPAE-1 (TR = 1.404), the TRs for all CPAEs (CPAE-1, CPAE-2, and CPAE-3) were much lower (TRs < 0.85). This indicates that the vinyl reaction extents of CPAEs are much higher than that of HPAE. However, considering that the CPAEs and HPAE (HPAE-1) have similar *M*_w,GPC_ (entries 1 to 4 in Table 1), the larger number of reacted vinyl groups in CPAE-1, CPAE-2, and CPAE-3 could only be consumed by the intramolecular reactions, thus forming cyclic structures. Otherwise, much higher *M*_w,GPC_ should be observed in these CPAEs (relative to HPAE-1) due to the occurrence of more intermolecular combinations.

**Figure 2:**
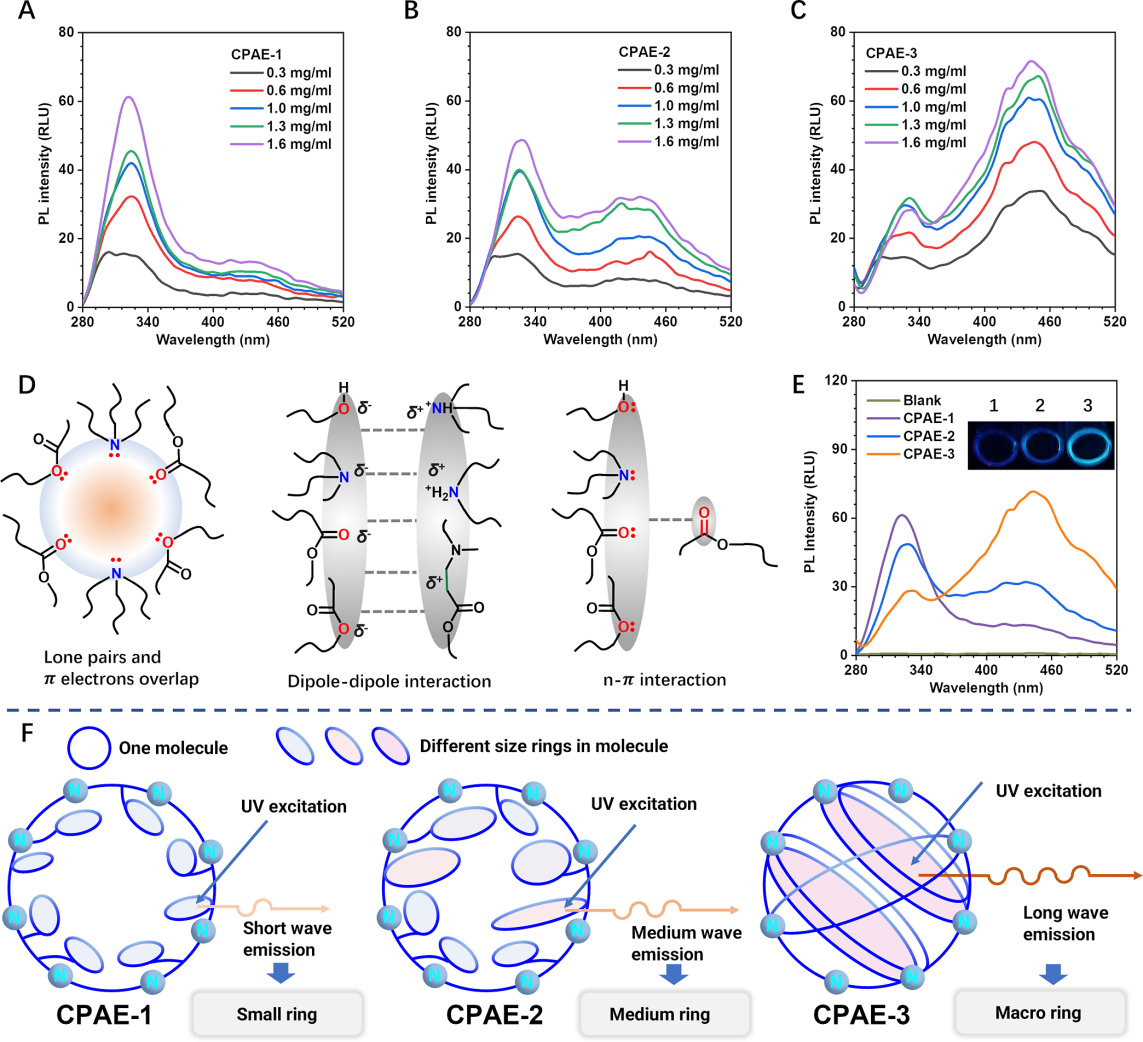
Distinct fluorescence phenomena of three CPAEs and their underlying mechanism. Different PL behaviors in relative light units (RLU) of (A) CPAE-1, (B) CPAE-2, and (C) CPAE-3 at different CPAE concentrations (in water). The excitation wavelength is 275 nm. (D) Schematic illustration of the electronic interactions in CPAE internal architecture. (E) PL behaviors in relative light units (RLU) of different CPAEs in water (1.6 mg/mL). (F) Schematic illustration of the relationship between different cyclic structures of CPAEs and their different PL behaviors.

The above results confirmed the general cyclic characteristic of CPAEs that is different from the branched structure, nevertheless, the differences in the specific cyclic structures of CPAE-1, CPAE-2, and CPAE-3 are not revealed. Chemicals containing carbon and nitrogen groups often have fluorescence characteristics, which are affected by the internal structure of the polymer. Inspired by that, the photoluminescence (PL) phenomena of CPAE-1, CPAE-2, CPAE-3 (and HPAE-1) were first examined to aid the understanding of the unique ring structures in CPAE-1, CPAE-2, and CPAE-3. As expected, even without conjugated groups, all PAEs emitted fluorescence when excited by ultraviolet light of 275 nm (Figures 2A–C, S31A). Moreover, the fluorescence intensity increased with the increase of PAE concentration, indicating a typical clustering-triggered emission (CTE) phenomenon.^23^ The mechanism of CTE suggests that the spacing between oxygen/nitrogen atoms in CPAEs/HPAE is less than the sum of their van der Waals radii, which leads to effective electronic interactions, resulting in spatial delocalization of non-covalent bonds, lengthening the effective conjugation distance, and resulting in fluorescence (Figure 2D).^24^ Interestingly, when the PL plots of three CPAEs are viewed in one image, an obvious distinction between their fluorescence behaviors can be observed—an increasingly significant red-shifted peak was observed from CPAE-1 to CPAE-3 (Figure 2E).

As the polymer emission wavelength depends on the conjugate plane caused by the spatial delocalization, a larger conjugate plane would make the emission wavelength longer, that is, red shift of the fluorescence. Also, the conjugate plane increases with the enhancement of aggregation.^25,26^ With the above insights, the distinct fluorescence phenomena displayed in Figure 2E should be correlated with the different internal molecular interactions^27^ in CPAE-1 to −3. Specifically, with a bigger ring size, more groups with lone pairs of electrons would be fixed in the denser cyclic structure, exhibiting a more red-shifted emission (Figure 2F). Therefore, compared to CPAE-2 and -3, the small ring structure in CPAE-1 (prepared from Method 1) can be reflected by the dominant fluorescence peak at ca. 325 nm (Figure 2A,F). CPAE-2 (synthesized from Method 2) also contains small rings (a relatively high emission peak at ca. 325 nm), while abundant medium-sized rings were also present in CPAE-2, as evidenced by the appearance of a long-wavelength fluorescence peak at ca. 430 nm (Figure 2B,F). For CPAE-3C [synthesized via the late dilution strategy (Method 3)], it exhibited a dominant long-wavelength emission peak at ca. 450 nm, confirming that macro rings are dominant in its cyclic structure (Figure 2C,F).

**Figure 3:**
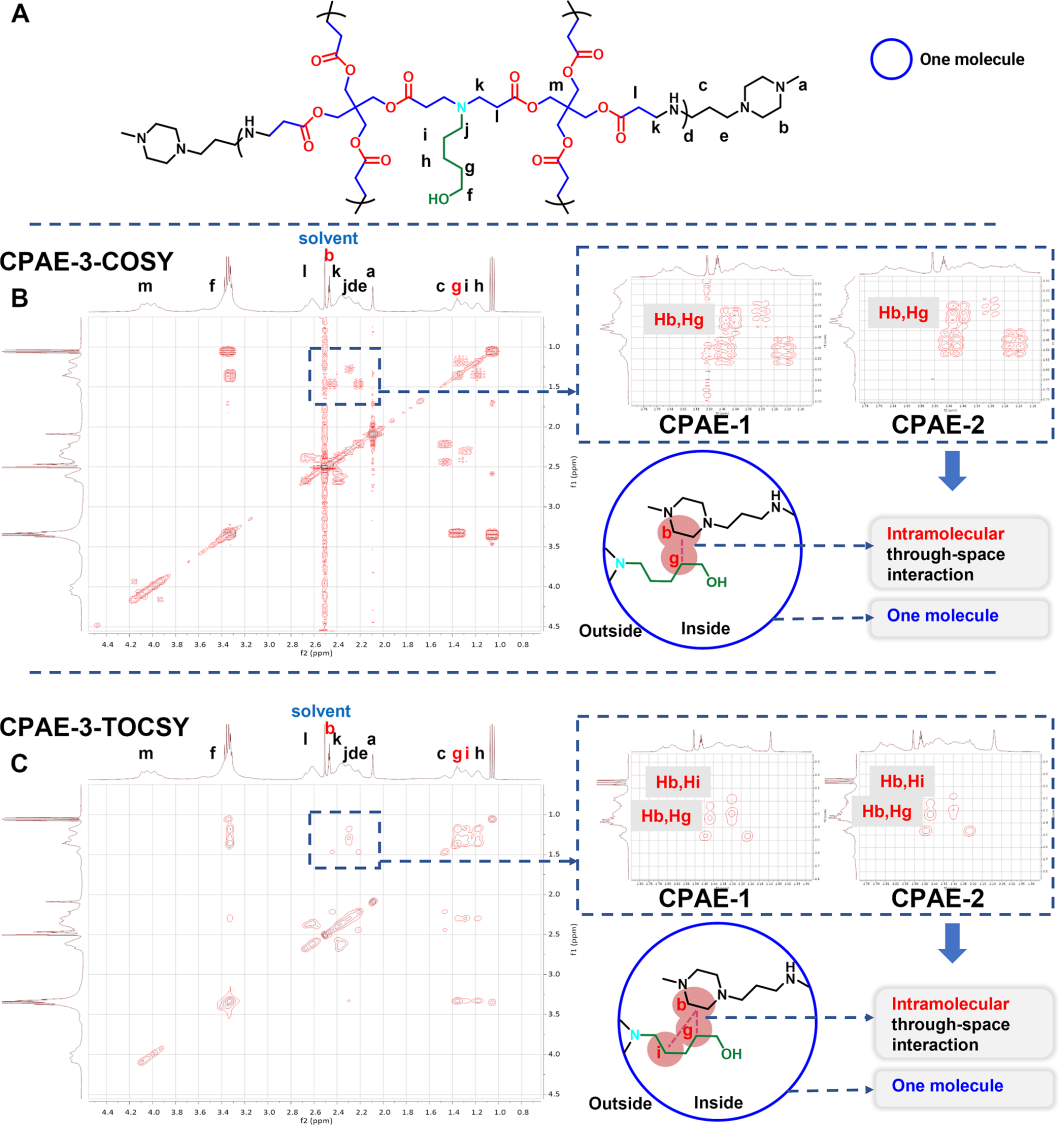
2D-NMR spectra of CPAE-1 to CPAE-3. (A) CPAE chemical structure with different protons labelled. (B) Short-range ^1^H–^1^H correlation spectroscopy of COSY of CPAE-1 to CPAE-3 (Figures S19–S21). (C) Long-range ^1^H–^1^H correlation spectroscopy of TOCSY for CPAE-1 to CPAE-3 (Figures S22–S24).

2D-NMR analysis was also conducted to give more information about the 3D topology of CPAE-1 to CPAE-3. The ^1^H–^1^H-correlated spectroscopy (COSY) spectra of CPAE-1 to CPAE-3 show that the proton of g (Hg) on S5 (Figure 3A) is coupled with the proton of b (Hb) on E7 for CPAE-1 and CPAE-2, meaning a strong through–space interaction between the terminal groups and the backbone in CPAE-1 and CPAE-2 (Figures 3B, S19 to S21). Additionally, the total correlation spectroscopy (TOCSY) revealed the corrective coupled peaks of Hb (E7) and the proton of i (Hi) on S5, Hb (E7), and Hg (S5) in CPAE-1 and CPAE-2; however, no such interaction was observed in the corresponding area for CPAE-3 (Figures 3C, S22–S24). These results indicate that most of the terminal groups in CPAE-1 and CPAE-2 are embedded in the CPAE backbones, while for the macro-ring-dominated CPAE-3, most of its terminal groups are pending outward, thus no interaction with the backbone structure was observed.

**Scheme 2:**
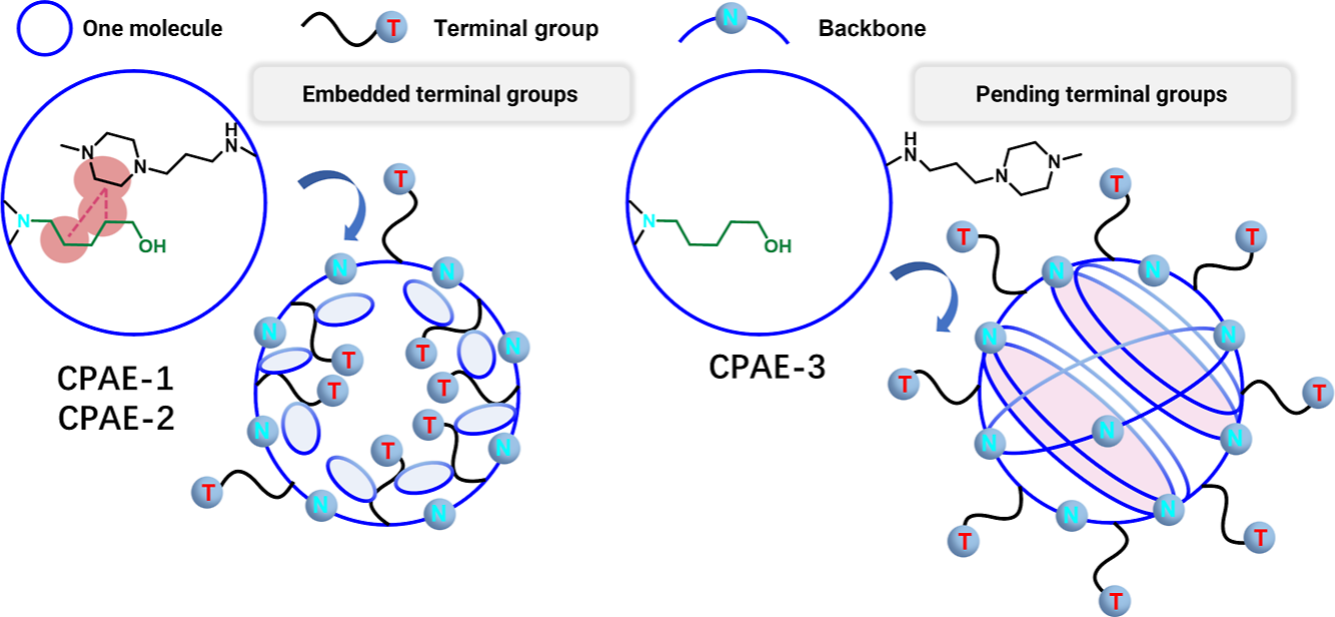
Schematic Illustration of the 3D Topologies of CPAEs with Different Cyclic Structures

The finding was further proved using the heteronuclear multiple bond correlation (HMBC) and heteronuclear single quantum coherence (HSQC) NMR spectroscopy (Figures S25–S30). In the HMBC spectra of CPAE-1 and CPAE-2, there are coupling peaks between the carbon of b (Cb) on E7 and Hg of the backbone monomer S5 (Figure S25 and S26). These peaks were, however, missing in the CPAE-3 spectra (Figure S27), where instead, the coupling peaks between carbon and proton atoms within the backbone monomer S5 [carbon of j (Cj) vs Hi and proton of h (Hh)] were found. These results further confirmed that the E7 groups were embedded within the molecule structure of CPAE-1 and CPAE-2, while pending outward in CPAE-3 which has a highly intense core. The highly intense core in CPAE-3 can be further verified by its HSQC spectra, where the coupling peaks between carbon of i (Ci) on S5 and Hg (S5) were absent due to the loss of signal caused by the interactions between the nuclear spin states of the different types of nuclei (Figure S30). This unique 3D topology of CPAE-3 can be attributed to the existence of numerous macro rings in its structure (as demonstrated in Figure 2 based on the fluorescence study), which locked the polymer backbone in a twisted conformation, forming a dense and highly cyclic core, and also the flexible terminal groups were squeezed outside on the surface (Scheme 2).

### Macrocyclic Structure-Boosted Gene Transfection

**Figure 4:**
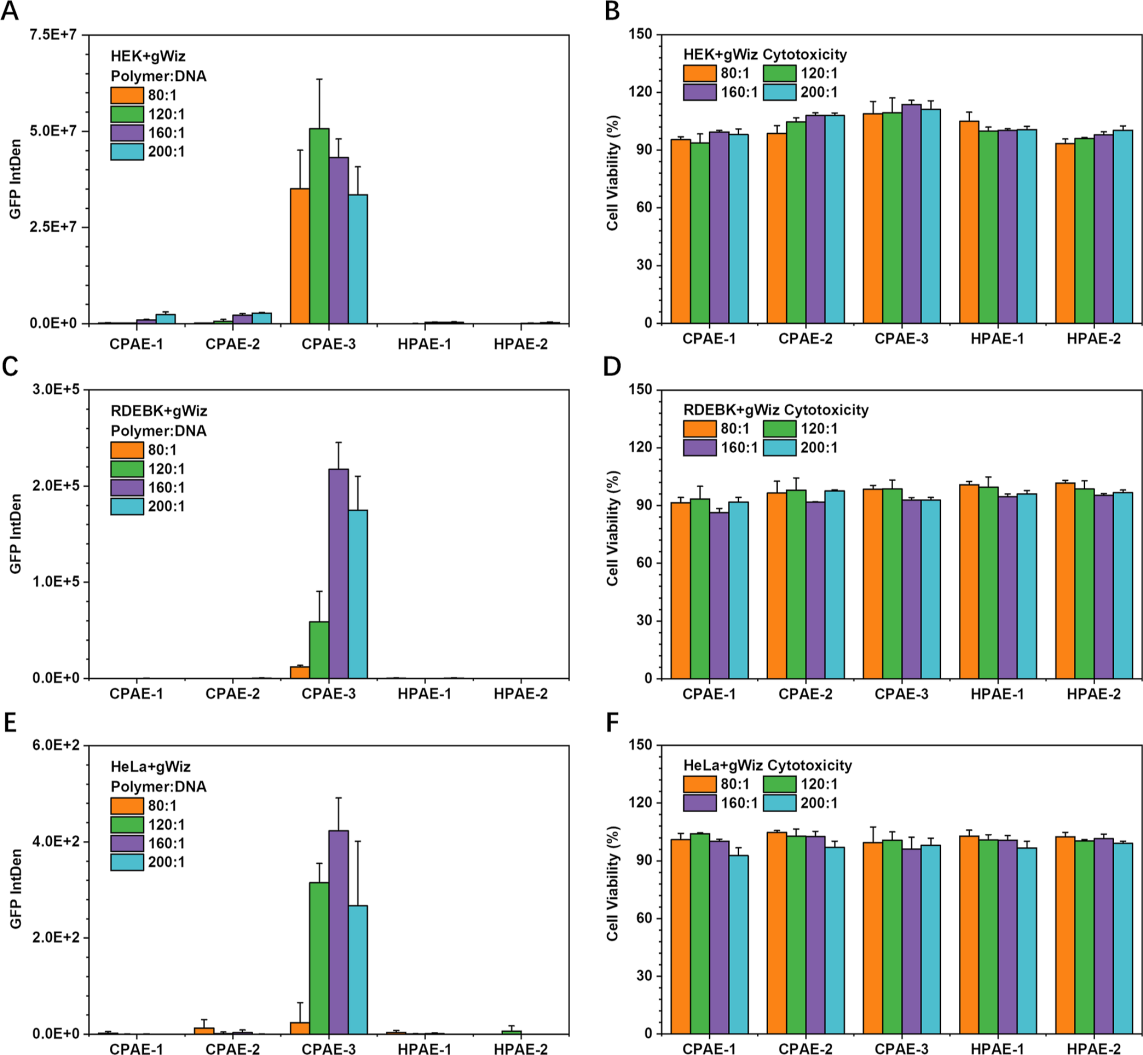
In vitro assessment of the gene transfection performance of CPAEs with different cyclic types (CPAE-1, CPAE-2, and CPAE-3, Table 1). GFP expression and cell viability of HEK (A,B), RDEBK (C,D), and HeLa (E,F) cells 48 h post-transfection by different PAE-based polyplexes. The transfection data of HPAE-1 and HPAE-2 (with reduced amount of E7 groups, Table 1) are also displayed for comparison.

Based on the above understanding of the cyclic topologies of three CPAEs, to probe their unique potential in gene delivery, the gene transfection capabilities of CPAE-1, CPAE-2, and CPAE-3 were evaluated using HEK, RDEBK, and HeLa cells. HPAE-2 with similar MW (*M*_w,GPC_ = 11,544 Da) to the CPAEs and reduced E7 groups (TR = 1.166) compared to HPAE-1 was also prepared for comparison (entry 5 in Table 1, Figures S16–S18). A series of polymer/DNA weight ratios (*w*/*w*, from 80:1 to 200:1) were used in the following transfection experiments. Green fluorescent protein (GFP) expression was used as a reporter for cell transfection (Figure 4). Surprisingly, over the range of tested polymer/DNA weight ratios and cell lines, a significantly higher transfection performance was observed in MCPAE (i.e., CPAE-3 with macro ring structure) than that of other CPAEs with smaller rings (CPAE-1 and CPAE-2) as well as the HPAEs (HPAE-1 and HPAE-2). In particular, CPAE-3, with polymer/DNA *w*/*w* of 120:1, showed a 21-fold, 18-fold, and 138-fold boost in transgene expression in HEK cells compared to the optimal groups of CPAE-1, CPAE-2, and HPAE, respectively (Figure 4A). Furthermore, no cytotoxicity was observed for CPAEs in all cell lines (over 90% cell viability), even under the highest polymer/DNA weight ratio (*w*/*w* = 200:1) (Figure 4B,D,E). These results clearly demonstrate the high potential of MCPAE in transgene expression.

**Figure 5:**
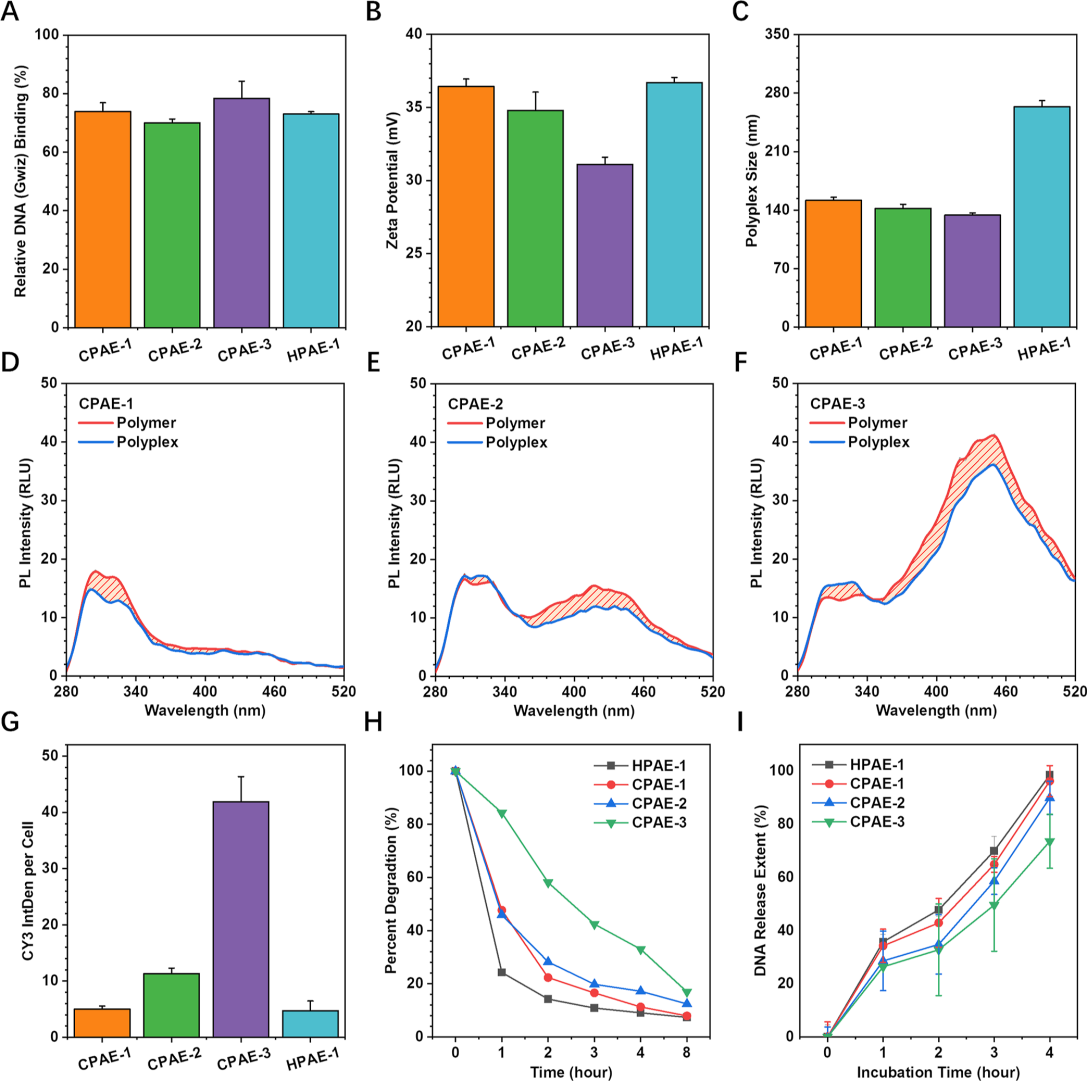
Physicochemical characteristics of CPAEs with different cyclic types in the key steps of transfection. (A) DNA binding affinity measurement using PicoGreen assay at *w*/*w* of 160:1. Polyplex zeta potential (B) and size (C) measurements at polymer/DNA weight ratio (*w*/*w*) of 160:1. The fluorescence behavior of (D) CPAE-1 polymer and polyplex, (E) CPAE-2 polymer and polyplex, and (F) CPAE-3 polymer and polyplex in 25 mM sodium acetate (pH = 4.8). The changes in fluorescence intensity were represented by calculating the differences of the integration areas (shaded area from 280 to 530 nm) of the polymer and polyplex PL intensity. (G) Cellular uptake of PAEs-based polyplexes 4 h post-transfection at *w*/*w* of 160:1. (H) Polymer degradation profile in 25 mM sodium acetate (pH = 4.8) determined using GPC (Figures S32–S35). The percent degradation was calculated according to the *M*_w,GPC_ (Table S1). (I) DNA release assessment from polyplexes formed by different CPAEs with PicoGreen assay in 25 mM sodium acetate (pH = 4.8). HPAE-1 was also displayed here for comparison.

To understand the underlying mechanism behind these distinct gene transfection behaviors, CPAE-1, CPAE-2, CPAE-3, and HPAE-1 (which has better transfection performance than HPAE-2) were further analyzed in terms of several key steps in gene transfection, that is, polyplex formation, cellular uptake, and DNA protection. Effective gene delivery depends on the formation of nano-sized polyplexes by vectors. Therefore, firstly, the interaction of CPAE-1, CPAE-2, CPAE-3, and HPAE-1 with DNA was evaluated by PicoGreen assay, dynamic light scattering (DLS), and the polyplex CTE behaviors. The PicoGreen assay results showed that all PAEs achieved a high DNA binding efficiency above 80% (the DNA binding of MCPAE, i.e., CPAE-3, is slightly higher than others) (Figure 5A). Although the DNA binding efficiencies of different PAEs were similar, according to the DLS evaluation, the cyclic structure of PAEs significantly helped condense the polyplex size compared to the branched structure (polyplex sizes < 150 nm of CPAE-1, -2, -3 vs 263 nm of HPAE-1) while maintaining high zeta potential (>30 mV for all PAEs) (Figure 5B,C). Moreover, the sizes of CPAE polyplexes decreased as the ring sizes of the corresponding polymers increased, for example, the CPAE-3-based polyplex exhibited the smallest size of 134 nm (Figure 5C). These variations in polyplex sizes formed by different PAEs demonstrate the stronger DNA condensation capability of CPAEs, especially the MCPAE. The polyplexes’ CTE behaviors were also investigated for different PAEs (Figures 5D–F, S31B). In general, due to the electrostatic interaction of positively charged PAEs and negatively charged DNA, the charge distribution and molecular orbital energy levels of PAE molecules were altered, obstructing the transition between the excited state and the ground state. Thus, compared to the fluorescence of PAE polymers, the fluorescence of their polyplexes all decreased (Figures 5D–F, S31B). Notably, by comparing the changes of fluorescence intensity of three CPAE polymers and the polyplexes formed by them (represented by the shaded area of PL intensity, Figure 5D–F), a more significant emission decrease was obtained in MCPAE (CPAE-3) (554 RLU, Figure 5F, 192 RLU of CPAE-1, Figure 5D, 288 RLU of CPAE-2, Figures 5E and 225 RLU of HPAE-1, Figure S31B). These findings suggest that the cyclic structure, particularly the macro ring structure (MCPAE), is more beneficial for nano-sized polyplex formation.

Secondly, in terms of another vital transfection procedure—cellular uptake—the behaviors of polyplexes formed by different CPAEs (CPAE-1 to CPAE-3) and HPAE-1 were studied. According to the fluorescence signal of Cy3-labeled DNA, it can be observed that the intracellular uptake of macro ring CPAE-3 far surpassed other PAEs; it was more than four times greater than that of the SCPAE (CPAE-1) and HPAE-1 after 4 h of treatment (Figure 5G). Given that the MCPAE has abundant pendent terminal groups on the molecule surface, it is reasonable to attribute the better cellular uptake performance of CPAE-3 to the interactions between the positively charged pendent terminal groups with the negatively charged phosphate of the cell membrane (Scheme 2). In addition to DNA packaging and cellular uptake, protecting DNA in endosomes is another challenge of PAE vectors due to their biodegradability nature. Impressively, due to the more intense MCPAE core, CPAE-3 was found to degrade much slower than other PAEs in sodium acetate solution (Figures 5H and S36, GPC traces with degradation are shown in Figures S32–S35). The DNA release rate from the polyplexes of different PAEs was also determined by PicoGreen assay. Consistently, due to the more stable structure of MCPAE, CPAE-3-based polyplex exhibited the slowest release rate, demonstrating its best DNA protection capability under acidic condition (Figure 5I).

Driven by the better MCPAE behavior observed from the above studies, the effect of macro rings on enhancing PAE gene transfection was investigated in more detail. A series of MCPAEs with different macrocyclic extents (MCPAE-0, -6, -12, -19, -31, and -72 h) were prepared by regulating the reaction time after dilution in Method 3 (Figures S37–S39 and Table S2). MCPAE-0h is the classic HPAE structure (obtained before dilution), while after dilution, the macrocyclic extent gradually increased from MCPAE-6 to −72 h. This increasingly obvious macrocyclic structure was reflected by the gradually enhanced CTE emission at long wavelength in Figure S40 (which is unique for the macro ring structure as demonstrated in Figure 2E) and the decreased TRs (decreased terminal groups E7, from 1.315 to 0.772 in Table S2). The polyplex formation and transfection behaviors of these MCPAEs were then carefully studied.

**Figure 6:**
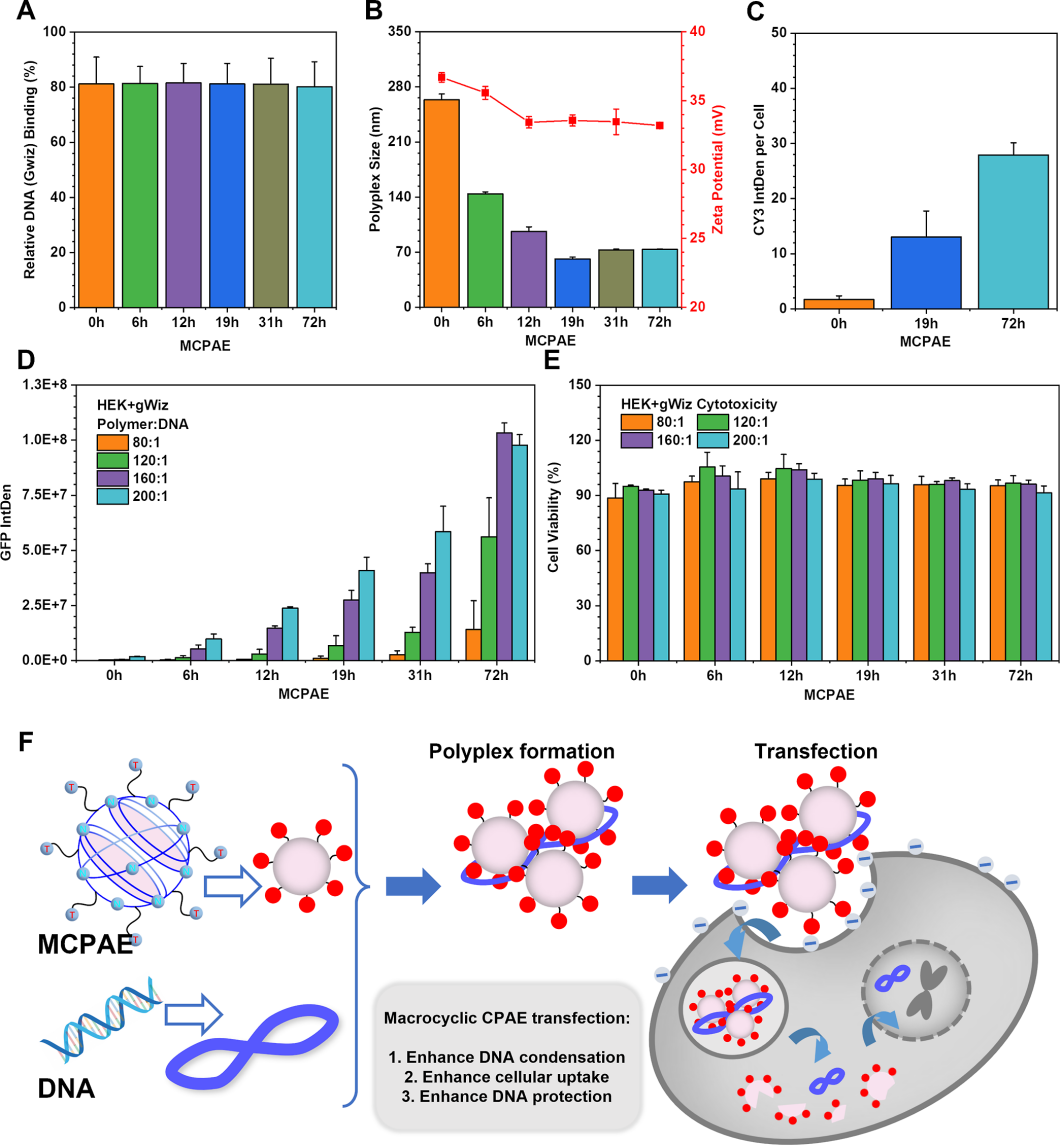
In vitro assessment of the gene transfection performance of MCPAEs with different macrocyclic extent (from 0 to 72 h after dilution in Method 3). (A) DNA binding at polymer/DNA weight ratio (*w*/*w*) of 160:1. (B) Size and corresponding zeta potential of polyplexes formed by different MCPAEs (0 to 72 h) at *w*/*w* of 160:1, 4 h post-transfection. (C) Cellular uptake efficiency of polyplexes from by different MCPAEs (0 to 72 h) with Cy3-labelled DNA at *w*/*w* = 160:1. (D) GFP expression of HEK cells 48 h post-transfection by polyplexes formed by MCPAE-0 to -72 h. € Cell viability of HEK cells 48 h post-transfection by polyplexes formed by MCPAE-0 to MCPAE-72 h at *w*/*w* of 160:1. (F) Schematic illustration of MCPAE enhanced transfection mechanism.

According to Figure 6A,B, while maintaining more than 80% DNA packaging efficiency and high surface potential (>33 mV), the polyplex sizes reduced by 70% from MCPAE-0 to MCPAE-72 h as the macro cyclization extent increased. Meanwhile, the polyplex uptake efficiency was improved more than 16-fold from MCPAE-0 to -72 h (Figure 6C). The GFP expression post-transfection of MCPAE-72 h enhanced 230-fold (*w*/*w* = 160:1) than MCPAE-0 h (*w*/*w* = 200:1) with the transition from branched structure to the macrocyclic structure while maintaining over 90% cell viability (Figure 6D,E). These results provide further evidence that the incorporation of macrocyclic structure in CPAEs is beneficial for DNA packaging, cellular uptake, DNA protection, and ultimately gene transfection. Therefore, we anticipate that MCPAEs can serve as a new generation of high-efficiency DNA delivery vector.

### Development of Highly Efficient MCPAE Gene Delivery Vectors and Their Application in Gene Therapy

#### Terminal Group Optimization

**Figure 7:**
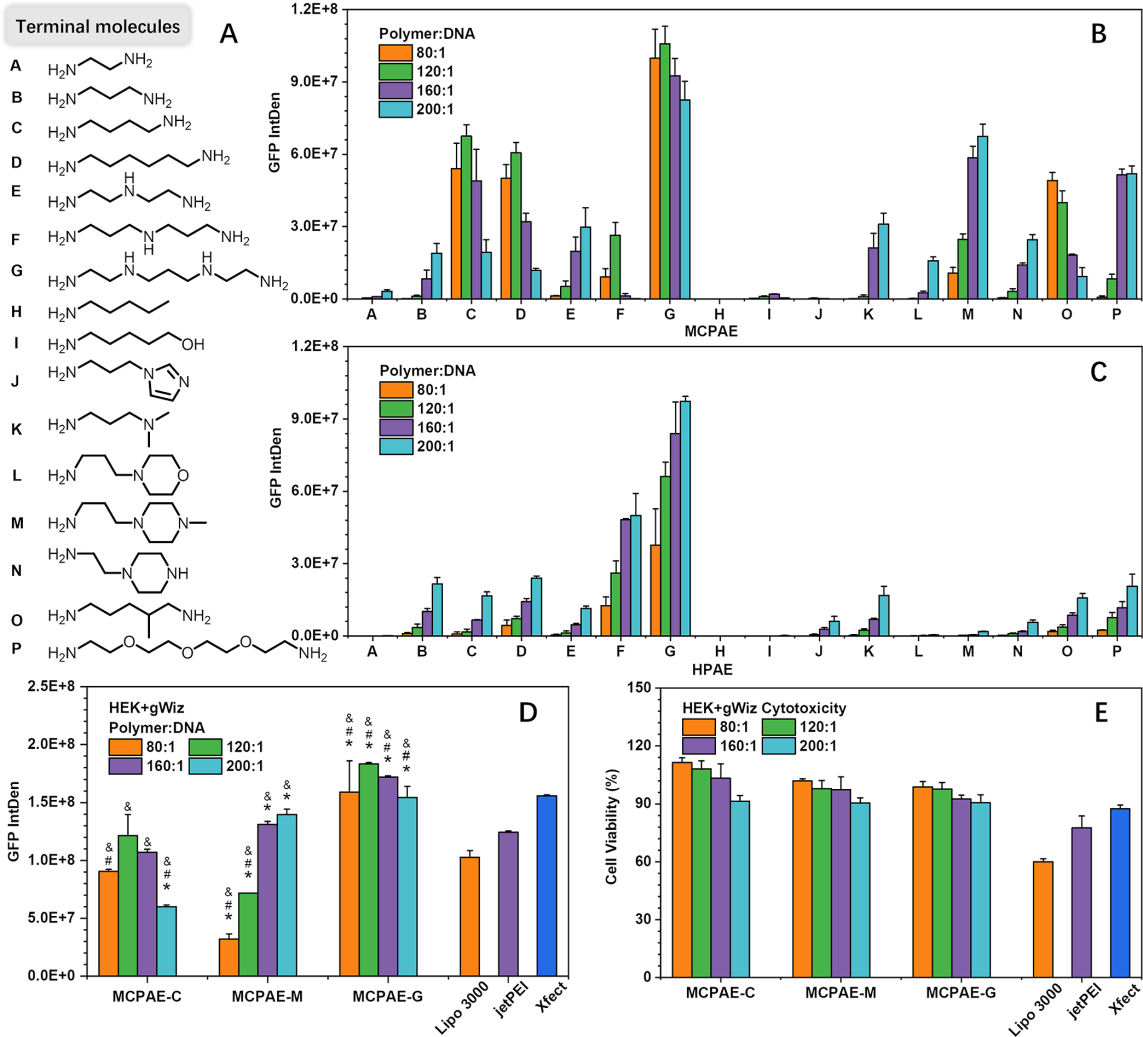
Transfection performance of MCPAEs and HPAEs with varied terminal groups. (A) The chemical structures of different terminal molecules. (B) Transfection of MCPAE-A to MCPAE-P with HEK cells. (C) Transfection of HPAE-A to MCPAE-P with HEK cells. GFP expression (D) and cell viability (E) of HEK cells 48 h post-transfection by different MCPAE and commercial polyplex agents. One-way ANOVA with data shown as average ± SD. Data points marked with asterisks (*) are statistically significant relative to the Lipo 3000 group, data points marked with pound key (#) are statistically significant relative to the jetPEI group, data points marked with ampersand (&) are statistically significant relative to the Xfect group. *P < 0.05 superior GFP expression compared with Lipo 3000; #*P* < 0.05 superior GFP expression compared with jetPEI, &*P* < 0.05 superior GFP expression compared with Xfect.

To further enhance the gene transfection performance of MCPAE, thus developing a new class of high-efficiency gene delivery vectors, the terminal groups were further screened and optimized for MCPAEs. Amine-containing terminal groups have been widely acknowledged to be beneficial for gene delivery.^28,29^ Herein, MCPAEs terminated with varied terminal amine-containing groups were constructed based on Method 3 (Figure 7A, MCPAE-A to -P, Figures S41, S42, S44 to S51, Table S3). For comparison, HPAEs with the same series terminal groups were also prepared (HPAE-A to -P, Figures S41, S43, S52 to S59, Table S4). The primary amino groups with different carbon chain lengths were first evaluated (terminal structure A to D, Figure 7A). The transfection results in Figure 7B showed that with the lengthening of the terminal carbon chains, the transfection efficacy of the terminated MCPAEs gradually increased and reached the highest performance with the terminal group C (four carbon lengths). Moreover, compared with HPAE-A to HPAE-D (Figure 7C), the transfection performances of MCPAE-A to MCPAE-D were much better, where MCPAE-C reached four times that of HPAE even at the optimal HPAE/DNA *w*/*w* ratio (200:1). Other primary and secondary amine-containing groups were also investigated (N, O, and P, Figure 7A). Regarding the GFP expression, the MCPAE group gave nearly three times higher expression than its corresponding HPAE group, and the transfection effect of MCPAE-P was comparable to that of MCPAE-D (Figure 7B,C).

Compared with primary amines, tertiary amines are more stable after protonation due to their stronger basicity, and their electrostatic interactions with DNA are stronger. Therefore, several tertiary amines were subsequently examined (K, L, and M, Figure 7A). Again, the transfection performance of MCPAE-K, -L, and -M far exceeded their HPAE counterparts; the transfection performance of MCPAE-M even surpassed the primary amino terminated MCPAE-C and -D at *w*/*w* of 200:1. Inspired by this superior performance of MCPAE-M, other terminal structures that contain multiple amine groups were evaluated (E, F, G, and N, Figure A). As expected, MCPAE-G with more amine groups showed excellent transfection ability (Figure 7B). Remarkably, it can be clearly seen in Figure 7B that MCPAE-G even achieved high transfection performance at a low polymer dosage (e.g., *w*/*w* = 80:1), in contrast, HPAE-G requires more than twice the polymer dosage to achieve a comparable transfection performance (Figure 7C). This phenomenon was also observed in other MCPAEs (such as MCPAE-C, -D, -O) and their corresponding HPAE structures. The reason for this phenomenon might be attributed to the larger number of pendent terminal groups located on the surface of MCPAEs, which would be conducive to the interaction with other species, including MCPAE molecules, DNA, and cell membrane, thus reducing the required polymer dosage. In addition, most of the MCPAEs exhibited high biocompatibility (>90% cell viability, Figures S60 and S61).

On the basis of the screening, the transfection efficiencies of the best performing polymers MCPAE-C, -G, and -M were further compared with three well-known commercial agents, Lipofectamine 3000 (Lipo 3000), jetPEI, and Xfect. As shown in Figure 7D, MCPAE-G exhibited the highest GFP expression, surpassing all commercial agents. MCPAE-C and -M also showed comparable or higher transfection capabilities than commercial agents. Additionally, all three MCPAEs displayed almost no cytotoxicity, demonstrating their potential in efficient and safe gene therapy (Figure 7E).

#### In Vitro Assessment of MCPAE-Mediated Gene Therapy for Recessive Dystrophic Epidermolysis Bullosa Treatment

**Figure 8:**
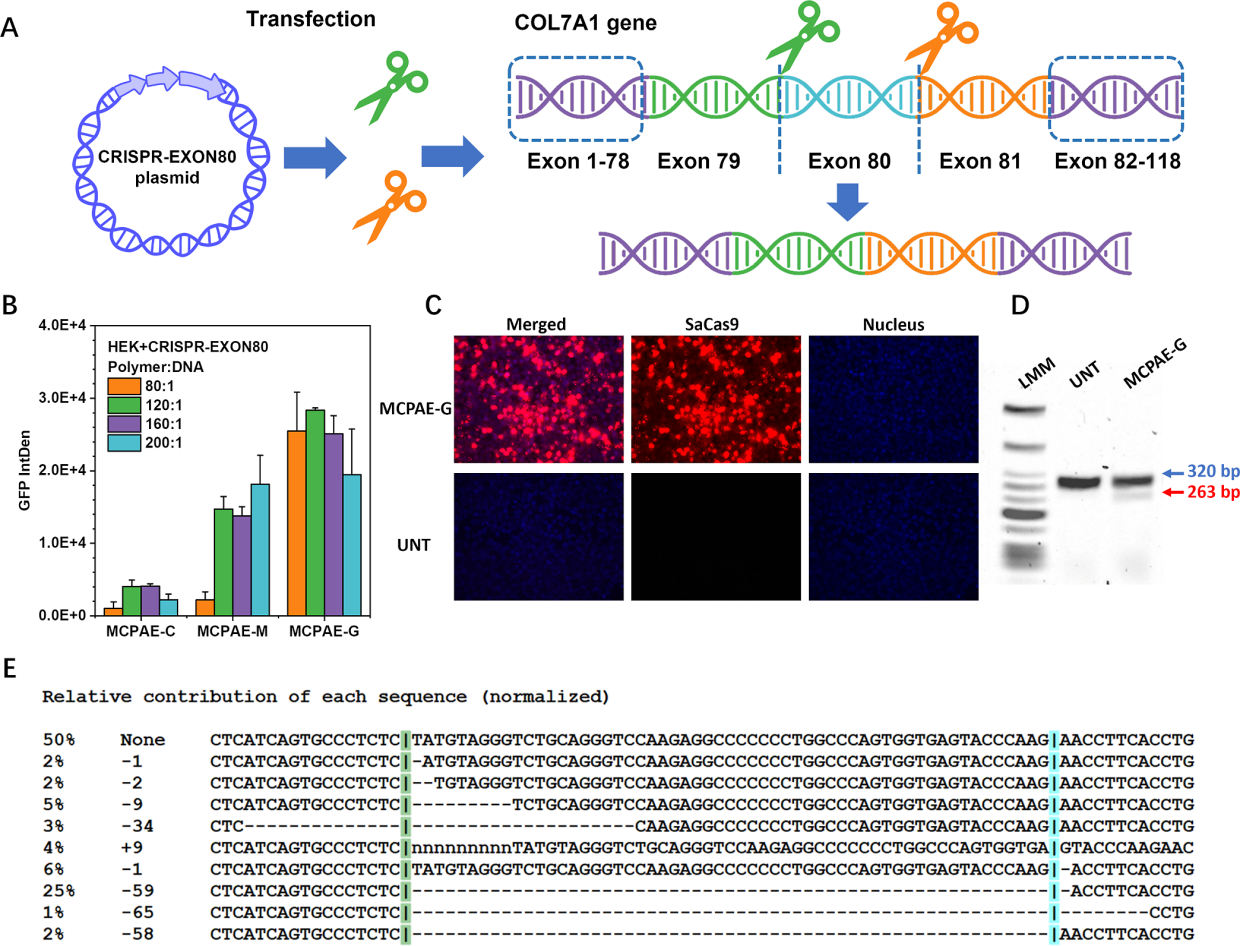
MCPAE-mediated in vitro gene editing assessment. (A) Schematic illustration of the gene editing process for targeted genomic deletion of pathogenic mutation-containing exon 80 in COL7A1 gene. (B) Transfection of CRISPR plasmid (containing + iresGFP expression cassette) using MCPAE-C, MCPAE-M, and MCPAE-G with different polymer/DNA weight (w/w) ratios. (C) Cas9 protein expression of HEK cells after incubation with MCPAE-G polyplexes. Samples were fixed and stained with Cas9 antibody in situ (red) and DAPI as the nuclear stain. (D) Surveyor mutation detector assay of HEK cells treated with MCPAE-G polyplex via agarose gel retardation. (E) Inference of CRISPR Edits analysis of Sanger sequencing data from MCPAE-G mediated CRISPR-EXON80 plasmid-transferred cells. Percentages indicate the proportion of the total DNA population with the indicated genotype.

Recessive dystrophic epidermolysis bullosa (RDEB) is one of the most severe subtypes of the rare debilitating skin disorder, which is caused by mutations in COL7A1 gene leading to absent, malformed or deficient type VII collagen (C7) and thus anchoring fibrils.^30,31^ This results in the separation of the epidermis from the dermis following minimal trauma or friction.^32,33^ Exon excision strategy has been developed as a promising approach to treat RDEB. For instance, exon 80 or exon 73, that are exons with high prevalence of mutations for the disease, have been successfully excised by the usage of CRISPR, achieving restoration of collagen VII levels.^34^ To evaluate the potential of the newly developed MCPAE vectors in the gene editing treatment of RDEB, three MCPAEs with the superior gene transfection performance, MCPAE-C, -G, and -M, were selected and applied to the CRISPR-EXON80 delivery for RDEB treatment (Figure 7B). The plasmid has 11 kb and codes GFP, *S. aureus* Cas9 nuclease (SaCas9) and dual CRISPR Cas9 single guide RNA (sgRNA) for human COL7A1 exon 80 gene excisions.^35^ The GFP gene is linked to the SaCas9 gene by internal ribosome entry site sequence and under the control of phosphoglycerate kinase (PGK) promoter. The main obstacle to the delivery of therapeutic genes, such as the CRISPR-EXON80 plasmid (Figure 8A), is their large sequence sizes.^36^ Meanwhile, editing two target positions simultaneously in this gene editing system requires a high level of delivery efficiency to ensure successful modification at both sites.^35^ According to the DLS analysis, MCPAE-C, -M, and -G all efficiently condensed the CRISPR-EXON80 plasmid into nano-sized polyplexes (<150 nm) with high zeta potentials (>33 mV) over different polymer/DNA weight ratios (from 120:1 to 200:1) (Figures S62 and S63). A reporter gene (GFP) was encoded on the CRISPR-EXON80 plasmid. Therefore, to screen for the most promising vector, these three MCPAEs were further applied to the GFP expression assessment. The results in Figure 8B show that compared to MCPAE-C and MCPAE-M, MCPAE-G displayed much better transfection performance while maintaining high cell viability over the range of tested polymer/DNA weight ratios (from 80:1 to 200:1) (Figure S64). Therefore, MCPAE-G polyplex was selected and further applied to the Cas9 production and localization investigation with immunocytochemistry (Figure 8C). Significant positive Cas9 staining is evident in cells treated with the MCPAE-G polyplex, which was predominantly localized around the nucleus, the action site for CRISPR gene editing.^37^ Based on the high-level Cas9 expression, MCPAE-G polyplex was then evaluated with the targeted genomic editing at a therapeutically relevant frequent mutation site. The expected edited band pattern was apparent in HEK293 cells transfected with CRISPR-EXON80 plasmid, on the contrary, this was not observed with the control DNA (Figure 8D). Inference of CRISPR Edits (ICE) analysis of Sanger sequencing results also confirmed the presence of a 58 bp fragment deletion in 25% of DNA sequences (Figures 8E and S65). The deleted fragment length at the targeted site matched the distance between target cut sites, inclusive of exon 80 in the COL7A1 gene,^34^ which demonstrates that the new developed MCPAE-G vector can successfully deliver complex therapeutic systems and achieve high-efficiency gene editing.

## Conclusion

In this work, a novel cyclization stage control strategy (Method 1, Method 2, and Method 3) was proposed to regulate the cyclization tendency at various SGP stages. The strategy enabled the construction of three types of 3D multi-cyclic PAEs with different ring sizes and cyclic topologies in a controlled manner. The unique topology characteristics of three CPAEs (including the different ring types and terminal group distribution etc.) were verified for the first time using fluorescence spectroscopy and 2D-NMR. The gene transfection results of the three types of CPAEs showed that the macrocyclic PAE (MCPAE) and its polyplex has considerably enhanced DNA condensation, cellular uptake, DNA protection, and thus the expression of transfected genes compared to other CPAEs and the HPAE counterparts. The top-performing MCPAE-C, -G, and -M exhibited higher transfection efficiencies than the best commercially available reagents Lipo 3000, jetPEI, and Xfect. Furthermore, the MCPAE with optimized terminal group was applied to efficiently deliver the CRISPR-EXON80 plasmid coding both *S. aureus* Cas9 nuclease and dual guide sgRNAs for in vitro gene editing. The findings from this work provide valuable insights to guide future development of high-efficiency non-viral polymeric gene delivery vectors.

## Supporting Information Available

Detailed experimental procedures, NMR spectra, GPC traces measurements, PL spectra, cytotoxicity assay, DLS measurements, sanger sequencing of edited gene, degradation analyses (PDF)
