# Supplementary material for: 3D Macrocyclic Structure Boosted Gene Delivery: Multi-Cyclic Poly(β-Amino Ester)s from Step Growth Polymerization
Source: J Am Chem Soc. 2023 Jul 25;145(31):17187–200. doi: 10.1021/jacs.3c04191 (PMC10416306; doi:10.1021/jacs.3c04191)
Supplement: Supplementary file 1 — ja3c04191_si_001.pdf [file ja3c04191_si_001.pdf]

Supporting Information

3D Macrocyclic Structure Boosted Gene Delivery: Multi-cyclic Poly(β-Amino Ester)s from Step Growth Polymerization

Yinghao Li,[1,2,†] Xianqing Wang,[2,†] Zhonglei He,[1,2] Melissa Johnson,[2] Sigen A,[*,2,3] Irene Lara-Sáez,[2] Jing Lyu,[*,2] and Wenxin Wang[*,1,2]

[1] *Research and Clinical Translation Center of Gene Medicine and Tissue Engineering, School of Public Health, Anhui University of Science and Technology, Huainan, 232001, China*

[2]*Charles Institute of Dermatology, School of Medicine, University College Dublin, Dublin 4, Dublin, D04V1W8, Ireland*

[3]*School of Medicine, Anhui University of Science and Technology, Huainan, 232001, China*

[†]These authors contributed equally.

1. Materials

For polymer synthesis and characterization, 5-amino-1-pentanol (S5), pentaerythritol tetraacrylate (PTTA), were purchased from Merck. 1-(3-aminopropyl)-4-methylpiperazine (E7) was purchased from Fisher Scientific. Other amines used in termination were all purchased from Merck. Lithium bromide (LiBr) for GPC measurements was purchased from Merck. Dimethyl sulfoxide (DMSO), dimethylformamide (DMF), acetone and diethyl ether were purchased from Fisher Scientific. Deuterated chloroform ($CDCl_3$) was purchased from Merck. Hank's balanced salt solution, and alamarBlue Assay Kit were purchased from Merck and Invitrogen, respectively. Xfect was purchased from Medical Supply Co. Ltd. Lipofectamine 3000 (Lipo 3000) transfection reagent was purchased from Bio-sciences. JetPEI were purchased from Polyplus Transfection, Illkirch-Graffenstaden, Strasbourg, France. Sodium acetate (Sigma) was diluted to 0.025 M prior to use. Picogreen was purchased from Life Technologies. Cell culture Dulbecco's modified Eagle Medium (DMEM) was purchased from Merck. Fetal bovine serum (FBS, Gibco) was filtered through 0.2 μm filters before use. gWiz-GFP commercial plasmid were obtained from Aldevron, Fargo, ND, USA.

2. Polymer Synthesis

PAEs were synthesized through a facile Michael addition reaction. The A4+B2 strategy was used, in which the tetrafunctional monomer PTTA referred to A4, S5 referred to B2, respectively. Vinyl and NH feed ratios for the synthesis of PAE base polymers are 1.2:1. Agilent 1260 Infinite gel permeation chromatography (GPC) and nuclear

magnetic resonance (NMR) were used to monitor the reaction.

HPAE Synthesis

HPAE-1, HPAE-2, and HPAE-A to P were obtained using the classical method. Typically, PTTA (3.52 g) and S5 (1.72 g) were dissolved in 12.2 mL DMSO and bubbled with argon for 15 minutes before the reaction occurred at 90 ℃. The reaction was stopped by diluting the mixture to 50 mg/mL with 92.6 mL DMSO when the weight average molecular weight ($M_{w,GPC}$) approached 10000 Da, and the reaction solution was split into two equal parts. To obtain product HPAE-1, E7 (1.07 g) was added to one part of the solution to endcap the acrylate-terminated base polymer at room temperature for 48 hours. For the control group HPAE-2, the other part of the solution was endcapped with equal molar amounts of E7 (0.78 g) and S5 (0.51 g). The resulting polymers were then precipitated in diethyl ether, dried under vacuum, and stored at −20 °C.

CPAE Synthesis Method 1

For the polymerization kinetic study, PTTA (3.52 g) and S5 (1.72 g) were dissolved in 47.1 mL DMSO and bubbled under argon for 15 minutes before the reaction was carried out at 90 ℃. To produce CPAE-1, the reaction was halted by diluting the mixture to 50 mg/mL with 52.5 mL DMSO when the desired $M_{w,GPC}$ was reached. The acrylate terminated base polymer was endcapped with E7 (3.14 g) at room temperature for 48 hours. The resulting polymers were precipitated into diethyl ether, dried under vacuum, and stored at −20 ℃.

CPAE Synthesis Method 2

For the polymerization kinetic study, PTTA (3.52 g) and S5 (1.72 g) were dissolved in 12.2 mL DMSO at concentrations of 300 mg/mL, respectively, and the solutions were bubbled with argon for 15 minutes. The PTTA solution was then heated to 90 °C, and the DMSO was added to the PTTA solution through a syringe pump at constant speeds (100 μL/min) until the solution was dilute to 70 mg/mL. To obtain product CPAE-2, the reaction was stopped by diluting the mixture to 50 mg/mL with 30.0 mL DMSO when the desired $M_{w,GPC}$ was reached. E7 (3.14 g) was then added to endcap the acrylate-terminated base polymer at room temperature for 48 hours. The resulting polymers were precipitated in diethyl ether, dried under vacuum, and stored at −20 °C.

CPAE Synthesis Method 3

For the polymerization kinetic study, typically, PTTA (3.52 g), and S5 (1.72 g) were dissolved in 12.2 mL DMSO (300 mg/mL). The solution was bubbled under argon for 15 mins and then the reaction occurs at 90 ℃. The first step was stopped by diluting the reaction solution to 50 mg/mL with 92.6 mL DMSO when $M_{w,GPC}$ was approaching the desired value. Then the reaction was carried on at 90 ℃ for another 3 days. To achieve the product CPAE-3, E7 (3.14 g) was added to endcap the acrylate terminated base polymer at room temperature for 48 h. After that, polymers were precipitated into diethyl ether and dried under a vacuum before being stored at −20 ℃.

Macrocyclic PAEs (MCPAEs) with Different Cyclic extent (MCPAE-0h to 72h) Synthesis

Method 3 was used to synthesize MCPAE-0h to 72h. PTTA and S5 were dissolved in 12.2 mL DMSO and bubbled with argon for 15 minutes before the reaction occurred at

90 °C. The first step was stopped by diluting the mixture to 50 mg/mL with 92.6 mL DMSO when the $M_{w,GPC}$ approached the desired value. The reaction was then continued for different times to achieve MCPAEs with varying macrocyclic extent. To obtain the product MCPAE-0h to 72h, one-sixth of the polymer solution was taken out at different time points. For instance, MCPAE-12h was obtained from the polymer solution after 12 hours of reaction in dilute solution. E7 was added to endcap the acrylate-terminated base polymer at room temperature for 48 hours. The resulting polymers were then precipitated in diethyl ether, dried under vacuum, and stored at −20°C.

HPAEs and MCPAEs with Different Terminal Groups Synthesis

PTTA, and S5 were dissolved in DMSO (300 mg/mL). The solution was bubbled under argon for 15 mins and then the reaction occurs at 90 ℃. Once the $M_{w,GPC}$ approached the desired value, the solution was diluted to 50 mg/mL with DMSO and split into two equal parts. For MCPAE-A to P, one part of the polymer solution was separated into several equal parts and endcapped with different terminal amines at room temperature for 48 hours. For MCPAE-A to P, the other part of the polymer solution reacted for another 3 days before being separated into several equal parts and endcapped with different terminal amines at room temperature for 48 hours. The resulting polymers were precipitated in diethyl ether, dried under vacuum, and stored at −20°C. Figure S41 to S43 show the GPC traces of the above synthesis.

3. Polymer Characterization Methods

Molecular Weight Measurements

Number average molecular weight ($M_{n,GPC}$), weight average molecular weight ($M_{w,GPC}$), and $Đ$ of polymers were determined by GPC equipped with a refractive index detector (RI), a viscometer detector (VS DP) and a dual angle light scattering detector (LS 15° and LS 90°). To monitor the molecular weight of polymers during the polymerization process, 20 μL of the reaction mixture was collected at different time points, diluted with 1 mL of DMF, filtered through a 0.2 μm filter and then measured by GPC. The columns (PolarGel-M, Edinburgh, UK, 7.5 mm × 300 mm, two in series) were eluted with DMF and 0.1% LiBr at a flow rate of 1 mL/min at 60 ℃. Columns were calibrated with linear poly(methyl methacrylate) (PMMA) standards.

Nuclear Magnetic Resonance (NMR)

The chemical structure and composition of polymers were confirmed with one- and two-dimensional NMR spectra of ^{1}H NMR, ^{1}H, ^{1}H-COSY, ^{13}C, ^{1}H-HSQC, ^{13}C, ^{1}H-HSQC, ^{1}H, ^{1}H-TOCSY, and ^{13}C NMR. Polymer samples were dissolved in $CDCl_3$. Measurements were carried out on a Varian Inova 400 MHz spectrometer (Edinburgh, UK). To monitor the reaction extent during the polymerization process, 100 μL of the reaction mixture was collected at different time points, diluted with 800 μL of deuterated solvent and then measured by NMR. The vinyl reaction extent was calculated based on ^{1}H NMR: Vinyl reaction extent = (1 − remained vinyl amount/ original vinyl amount)*100% = $[1 - (I_a) / (I_m / 2)]$*100%, where I_a and I_m stand for the integral intensity of peaks a and m in ^{1}H NMR spectrum.

Polyplex Preparation

Generally, the polymers were initially dissolved in DMSO to stock solutions (100 mg/mL), and then the stock solutions were further diluted with 25 mM sodium acetate buffer according to the w/w ratio. DNA was diluted to 0.1 mg/mL with sodium acetate buffer. The polymer solutions were added into the DNA solution, vortexed for 10 s, and allowed to stand for 15 min.

Picogreen Assays

The polyplex were prepared as described above. 2 μg of DNA was used for each sample preparation. Then, 60 μL of Picogreen solution, which was prepared according to supplier's instructions, was added and allowed to incubate for another 5 min. To a 96-well plate, 200 μL of medium (without serum) or water was added, and then 30 μL of the polyplex solution was added. Fluorescence measurements were carried out with a plate reader with an excitation at 490 nm and emission at 535 nm. DNA binding efficiency (BE) was calculated as Equation (1)

$$BE = {(F_{DNA} - F_{Sample})}/{(F_{DNA} - F_{Blank})} \quad (1)$$

F_{DNA} was the fluorescence measurement of free DNA without polymer, F_{Sample} was the fluorescence of a polyplex at a given weight ratio between polymer to DNA, and F_{Blank} was the fluorescence from PicoGreen working solution only with the buffer used for polyplex formulation.

Size and Zeta Potential of Polyplexes

The polyplex was prepared as described above. After that, the sizes and zeta potentials

of polyplexes were measured with a Malvern Panalytical Zetasizer (ZTS1240). All the measurements were repeated three times.

Polymer Degradation Analysis

Polymers were first prepared as stock solution in DMSO (100 mg/mL). For each polymer, 100 μL stock solution was dissolved in 100 mL 25 mM sodium acetate. Then the solution was incubated at 37 °C for different times. For each measurement, 10 mL stock solution was collected and freeze-dried. The molecular weight information of polymer was measured by GPC. The $M_{w,GPC}$ and $M_{n,GPC}$ percent degradation were calculated as Equation (2) and (3)

$$Percent\ degradation\ (M_{w,GPC,different\ time\ point}) = \left(1 - {M_{w,GPC,different\ time\ point}}/{M_{w,GPC,0h}}\right) * 100\%$$

(2)

$$Percent\ degradation\ (M_{n,GPC,different\ time\ point}) = \left(1 - {M_{n,GPC,different\ time\ point}}/{M_{n,GPC,0h}}\right) * 100\%$$

(3)

Fluorescence Assessment

For the polymer measurement, the polymers were initially dissolved in DMSO to stock solutions. Then the 50 μL stock solution was diluted in 3mL water. For the polyplex and polymer comparison, the polyplexes were prepared as described above at a 160:1 polymer to DNA ratio. Then the 100 μL polyplex solution was diluted in 3 mL water. Equal amount of polymer was diluted in 100 μL sodium acetate, then further diluted in 3 mL water for measurement. Fluorescence measurements were carried out with a plate

reader with excitation at 275 nm.

Cell Culture

Human recessive dystrophic epidermolysis bullosa keratinocyte cells (RDEBK) were cultured using standard cell culture techniques in keratinocyte growth complete FAD medium (KCa). Human Embryonic Kidney 293 cells (HEK293) and human cervical cancer cells (HeLa) were cultured in Dulbecco's modified Eagle Medium high glucose containing 10% fetal bovine serum and 1% Penicillin-Streptomycin. Cells were cultured at 37 ℃ with 5% CO_2 in a humid incubator, under standard cell culture techniques.

Cytotoxicity Assessment (alamarBlue assay)

To perform alamarBlue assay, cell supernatants were first removed and then cells were washed with HBSS, followed by the addition of 10% alamarBlue reagent in HBSS. Living, proliferating cells maintain a reducing environment within the cytosol of the cell, converting the non-fluorescent ingredient resazurin in alamarBlue to the highly fluorescent compound resorufin. This reduction results in a color change from blue to light red and allows for the quantitative measurement of cell viability, based on the increase in overall fluorescence and color of the media. The alamarBlue solution from each well was transferred to a fresh flat-bottomed 96-well plate for fluorescence measurements at 590 nm. Control cells without any treatment were used to normalize the fluorescence values and plotted as 100% viable.

Polyplex Cellular Uptake

GFP DNA was labelled with a Cy3 (a red fluorescent dye) labelling kit as per the

recommended protocol. HEK cells were seeded in 96-well plates. Gene transfection was conducted as above with 2.5 μg/mL of DNA. After 4 hours, the medium was removed, and cells were washed with PBS three times and incubated with Hoechst 33342 for 30 mins. Fluorescent images of Cy3 and DAPI were visualized under a fluorescence microscope (Olympus IX81). The intensity of Cy3 fluorescence and cells number in each image were analyzed using the ImageJ Fiji software (NIH, Bethesda, MD, USA).

Cell Transfection

Cells were transfected with polyplexes prepared as described above, mixed with the cell culture medium, and added to cells at DNA concentration of 5 μg/mL. Expression of the GFP reporter gene green fluorescent protein (GFP) was visualized 48 h after transfection using an Olympus IX81 fluorescence microscope. The intensity of GFP fluorescence was analysed and semi-quantified using the ImageJ Fiji software.

Immunocytochemistry of SaCa9

Cells were pre-seeded in 24-well plates containing 12 mm coverslips and transfected as described above. After 72 hours incubation, cells were washed three times in ice-cold phosphate buffer saline and then fixed with ice-cold acetone: methanol for 20 min at -20 °C. Samples underwent three washes with PBS, to be then blocked in 3% bovine serum albumin and incubated with a rabbit anti-SaCas9 primary antibody at a dilution of 1:500. The coverslips were then incubated with AlexaFluor™ 568-labeled secondary antibody at 1:800 dilution, mounted on microscope slides with Fluoroshield mounting medium with DAPI, and imaged using an Olympus IX83 microscope.

Exon Excision Efficiency Evaluation

Cells were seeded and transfected with CRISPR-EXON80 plasmid (Centro de Investigaciones Energéticas, Medioambientales y Tecnológicas-CIEMAT, Madrid, Spain) in 24-well plate and the genomic DNA for treated cells were extracted 72 hours after transfection using lysis buffer (Tris [pH 8] 100 mM, EDTA 5 mM, SDS 0.2%, NaCl 200 mM and 1 mg/mL proteinase K; Merck, Kenilworth, NJ, USA) and ethanol precipitation. The target gene was amplified using DNA polymerase master mix. The PCR products was run and visualized on 1.8% agarose gel. The PCR products were also sent to Eurofins Genomics for Sanger sequencing. Inference of CRISPR Edits (ICE) analysis (Synthego, Redwood City, CA, USA) was used to confirm editing events and to track formation of insertion–deletion mutations (indels) in a pooled cell population.

4. Experiment Data

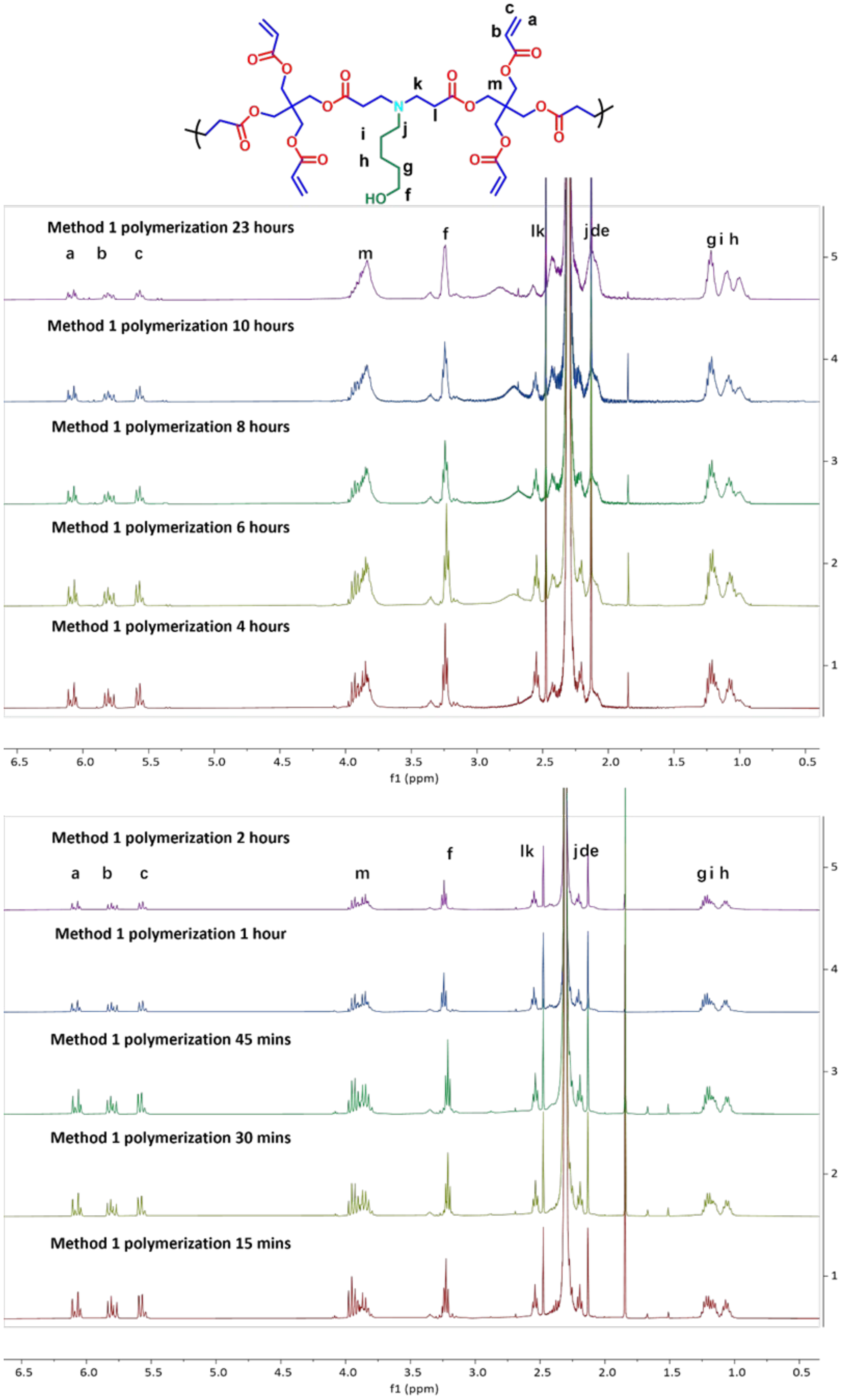

Figure S1. ^{1}H NMR spectra of CPAE synthesized by Method 1 for polymerization kinetic study.

Vinyl reaction extent = (1 − remained vinyl amount/ original vinyl amount)*100% = [1 − (I_a) / (I_m / 2)]*100%, where I_a and I_m stand for the integral intensity of peaks a and m in ^{1}H NMR spectrum.

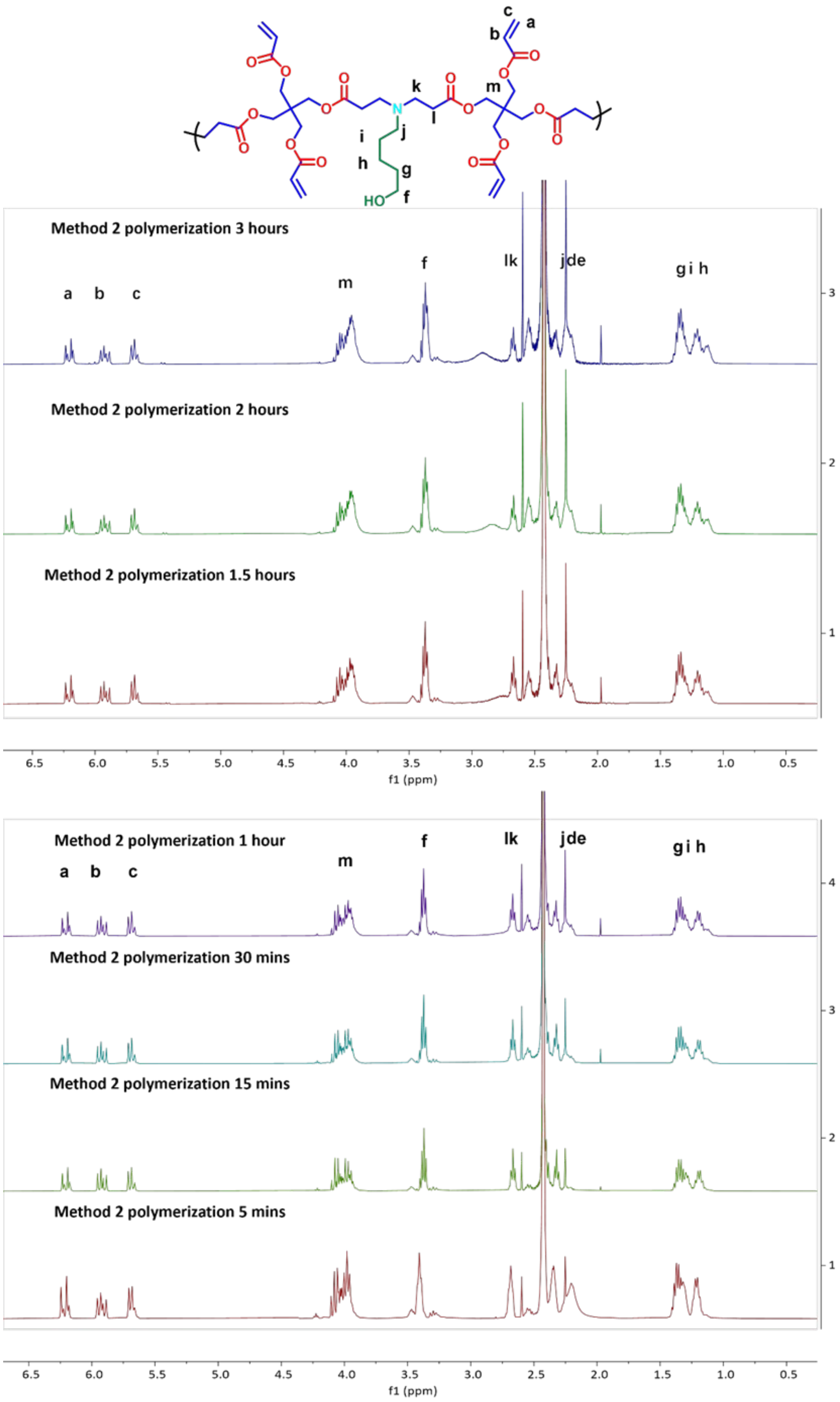

HO
Method 2 polymerization 3 hours
Method 2 polymerization 2 hours
Method 2 polymerization 1.5 hours
a b c
m
f
lk
jde
gi h
6.5 6.0 5.5 5.0 4.5 4.0 3.5 3.0 2.5 2.0 1.5 1.0 0.5
f1 (ppm)
Method 2 polymerization 1 hour
Method 2 polymerization 30 mins
Method 2 polymerization 15 mins
Method 2 polymerization 5 mins
6.5 6.0 5.5 5.0 4.5 4.0 3.5 3.0 2.5 2.0 1.5 1.0 0.5
f1 (ppm)

Figure S2. ^{1}H NMR spectra of CPAE synthesized by Method 2 for polymerization kinetic study.

Vinyl reaction extent = (1 − remained vinyl amount/ original vinyl amount)*100% = [1 − (I_a) / (I_m / 2)]*100%, where I_a and I_m stand for the integral intensity of peaks a and m in ^{1}H NMR spectrum.

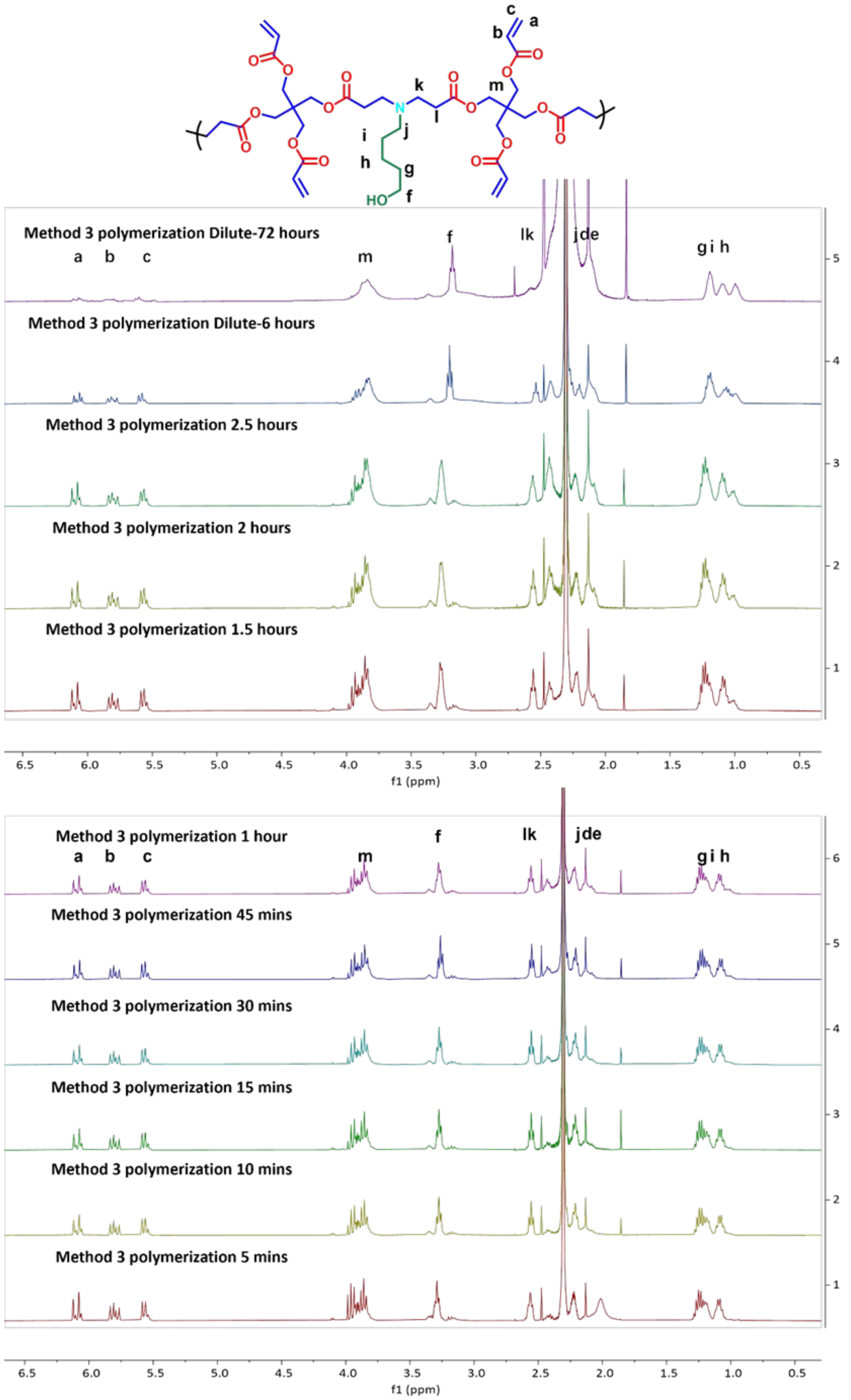

Method 3 polymerization Dilute-72 hours
Method 3 polymerization Dilute-6 hours
Method 3 polymerization 2.5 hours
Method 3 polymerization 2 hours
Method 3 polymerization 1.5 hours
Method 3 polymerization 1 hour
Method 3 polymerization 45 mins
Method 3 polymerization 30 mins
Method 3 polymerization 15 mins
Method 3 polymerization 10 mins
Method 3 polymerization 5 mins
a b c
m
f
lk
jde
gi h
f1 (ppm)

Figure S3. ^{1}H NMR spectra of CPAE synthesized by Method 3 for polymerization kinetic study.

Vinyl reaction extent = (1 − remained vinyl amount/ original vinyl amount)*100% = [1 − (I_a) / (I_m / 2)]*100%, where I_a and I_m stand for the integral intensity of peaks a and m in ^{1}H NMR spectrum.

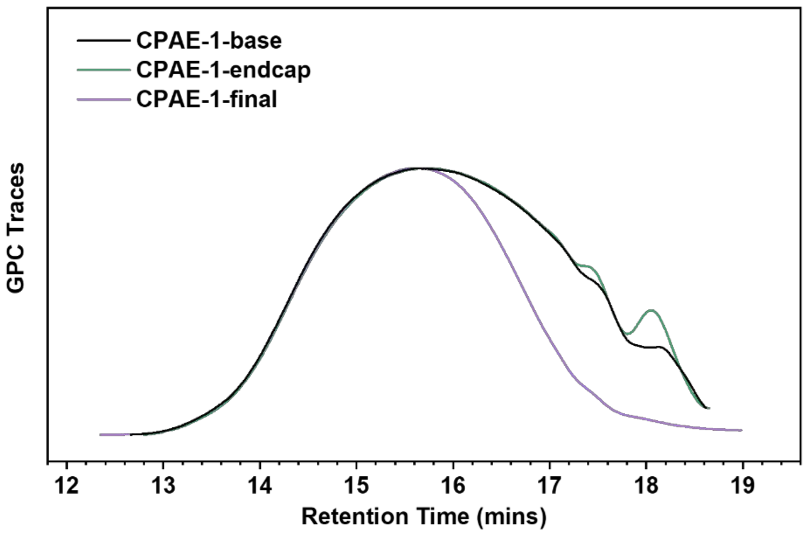

Figure S4. GPC traces of CPAE-1 synthesized by Method 1.

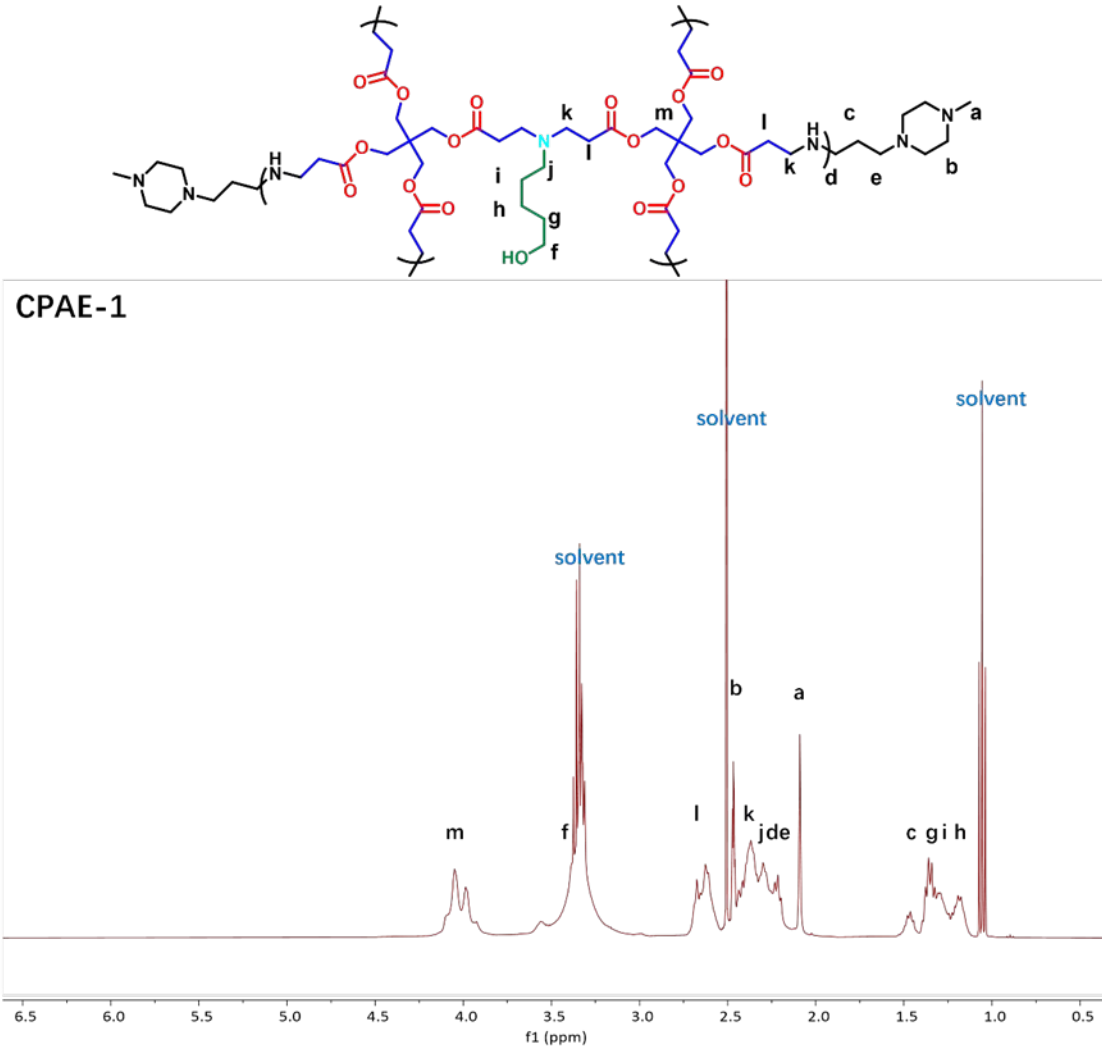

Figure S5. ^1H NMR spectra of CPAE-1 synthesized by Method 1.

Terminal ratio (TR) = [E7] / [PTTA] = $[(I_a) / 3] / [I_m / 8]$, where I_a and I_m stand for the integral intensity of peaks a and m in ^1H NMR spectrum.

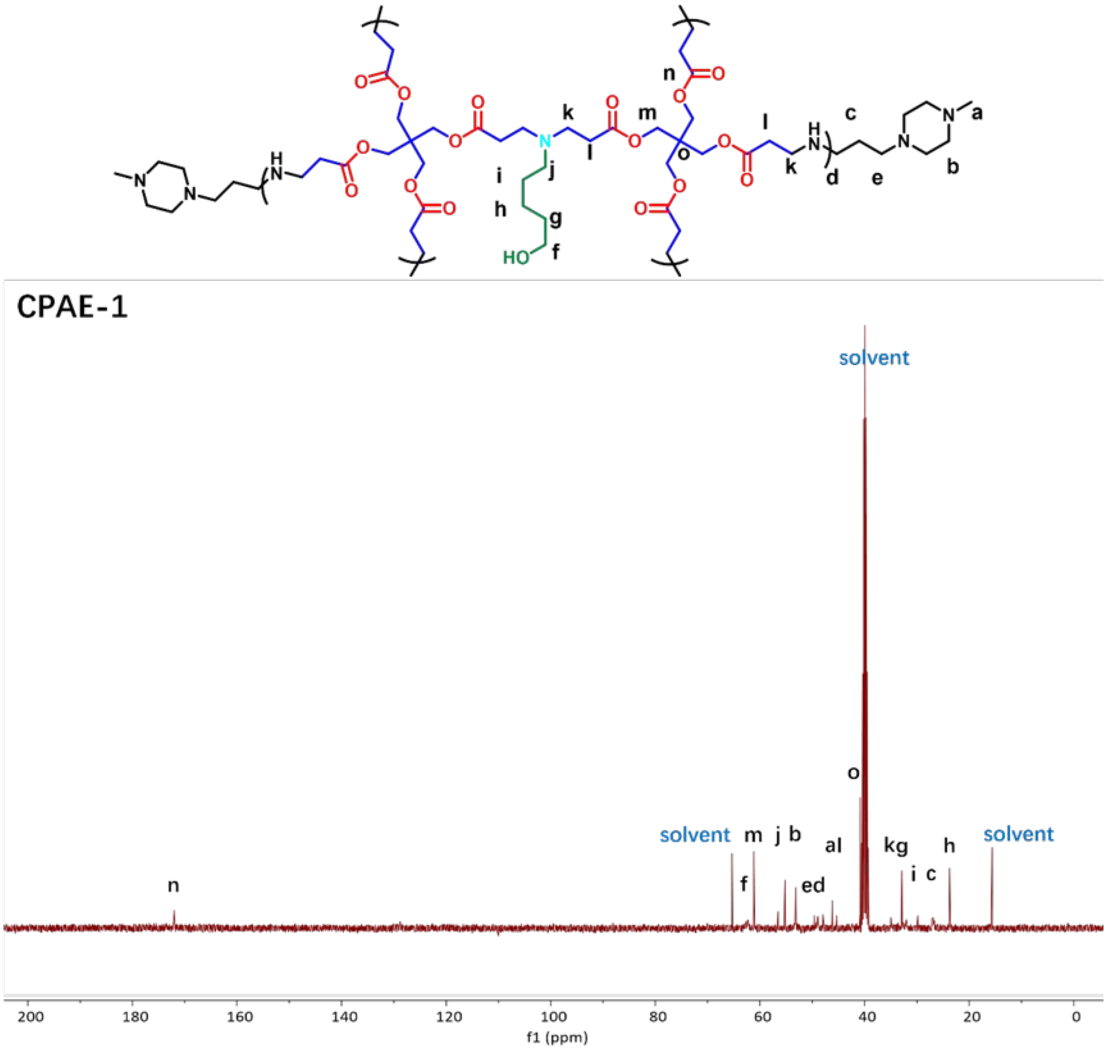

Figure S6. ^{13}C NMR spectrum of CPAE-1 synthesized by Method 1.

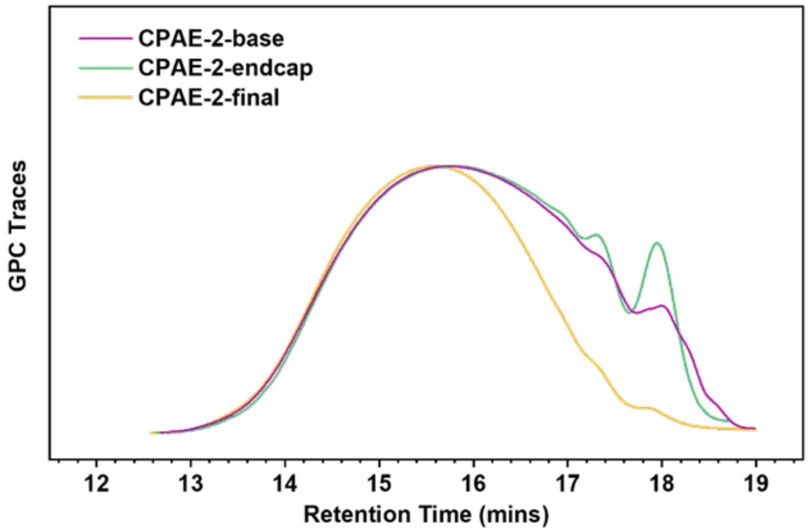

Figure S7. GPC traces of CPAE-2 synthesized by Method 2.

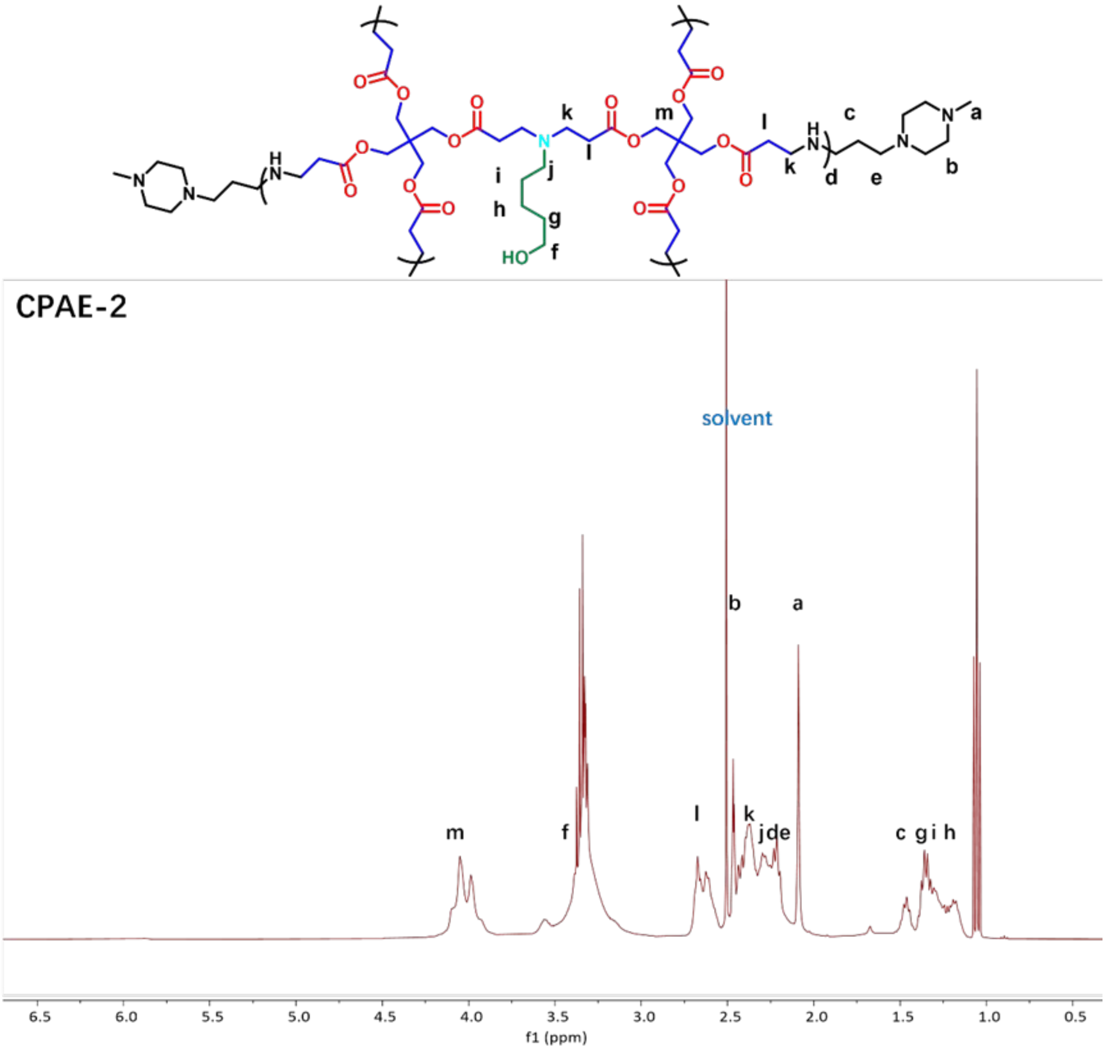

Figure S8. ^{1}H NMR spectrum of CPAE-2 synthesized by Method 2.

Terminal ratio (TR) = [E7] / [PTTA] = [(I_a) / 3] / [I_m / 8], where I_a and I_m stand for the integral intensity of peaks a and m in ^{1}H NMR spectrum.

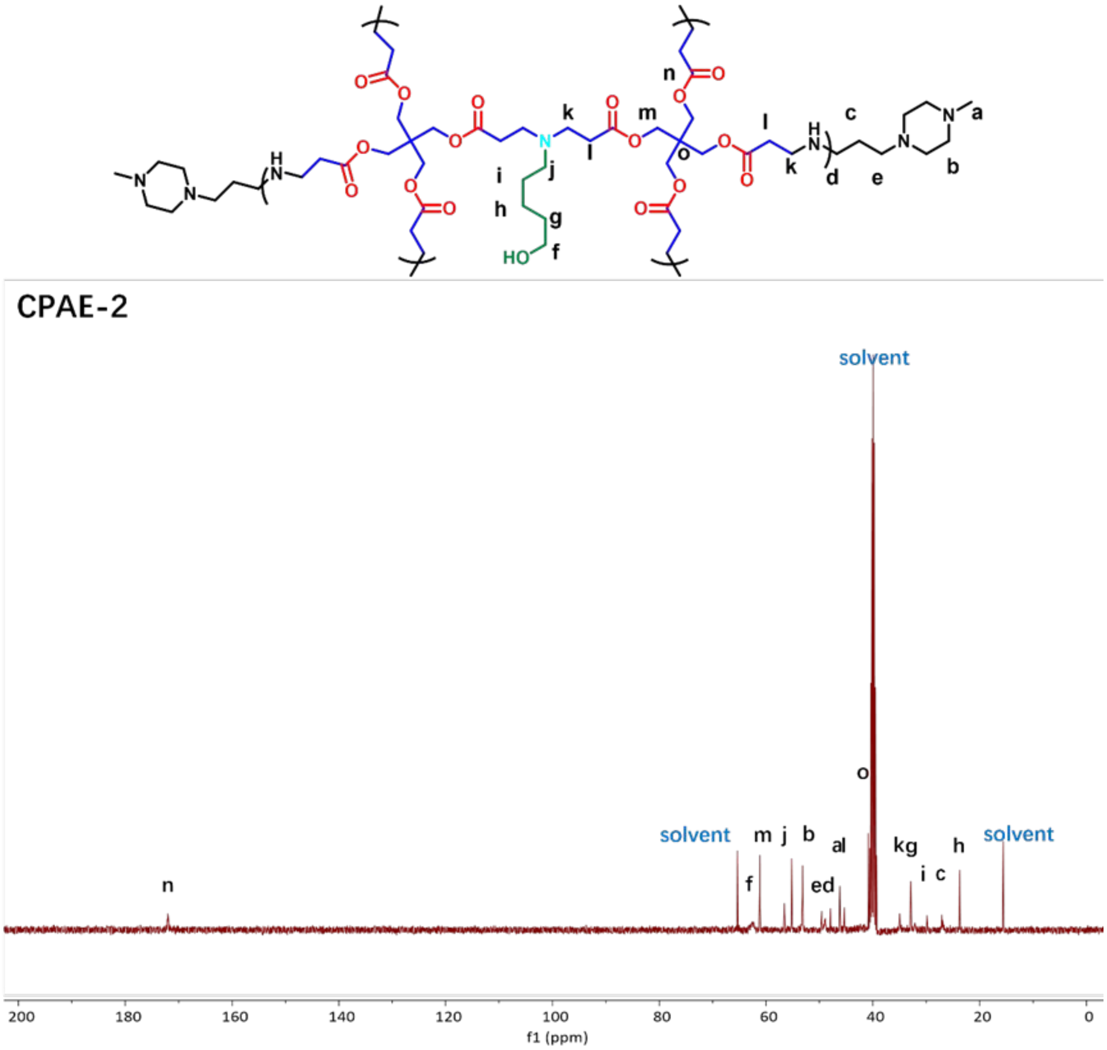

Figure S9. ^{13}C NMR spectrum of CPAE-2 synthesized by Method 2.

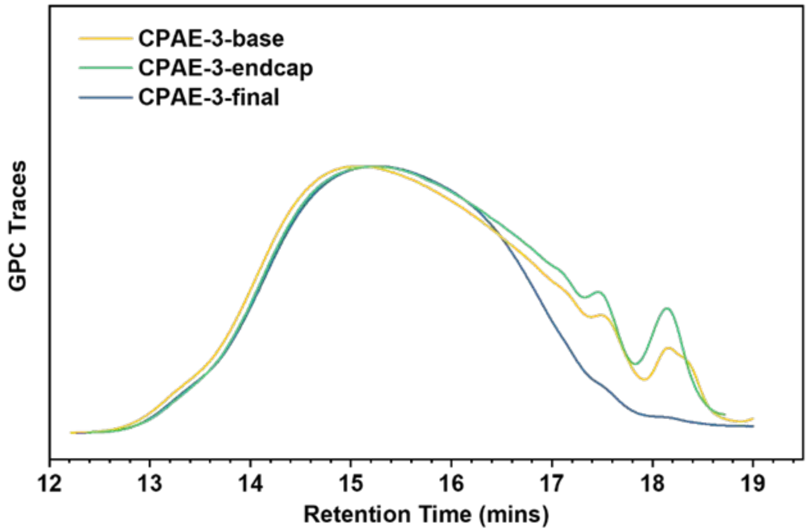

Figure S10. GPC traces of CPAE-3 synthesized by Method 3.

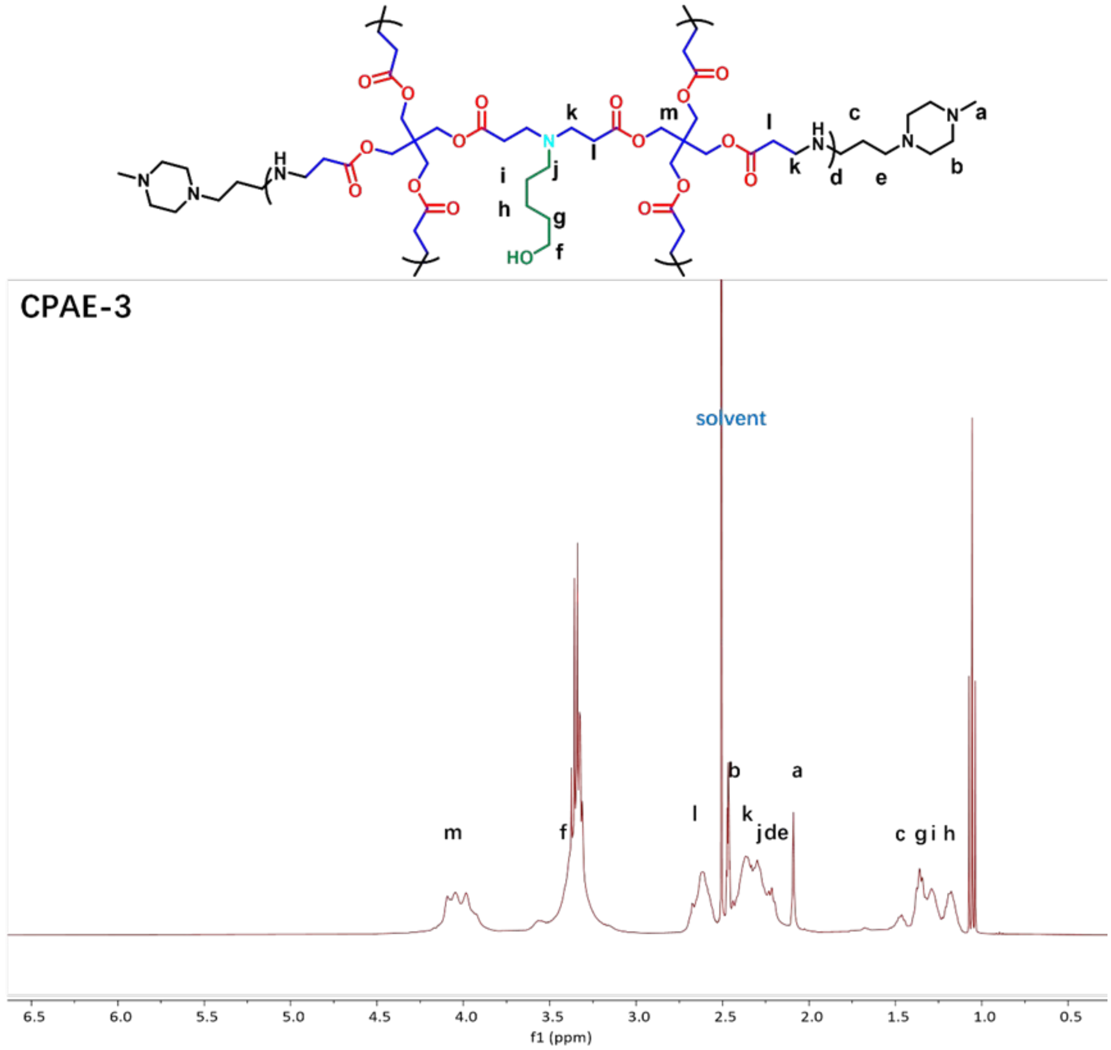

Figure S11. ^{1}H NMR spectrum of CPAE-3 synthesized by Method 3.

Terminal ratio (TR) = [E7] / [PTTA] = $[(I_a) / 3] / [I_m / 8]$, where I_a and I_m stand for the integral intensity of peaks a and m in ^{1}H NMR spectrum.

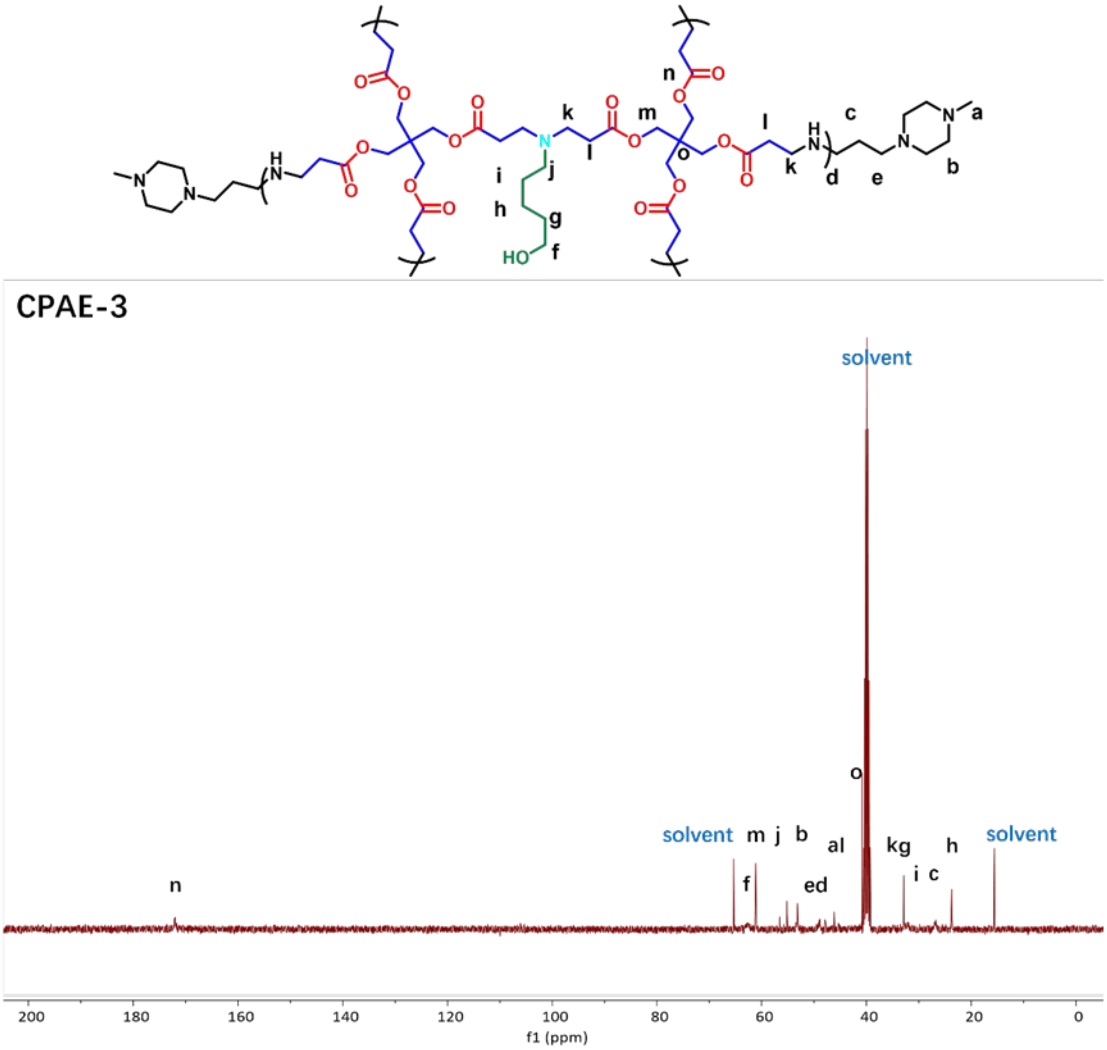

Figure S12. ^{13}C NMR spectrum of CPAE-3 synthesized by Method 3.

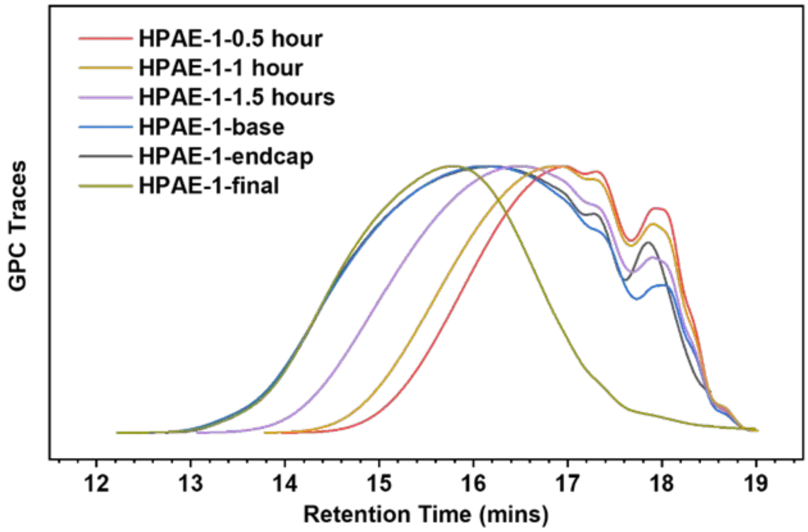

Figure S13. GPC traces of HPAE-1 synthesized by the classic method.[9]

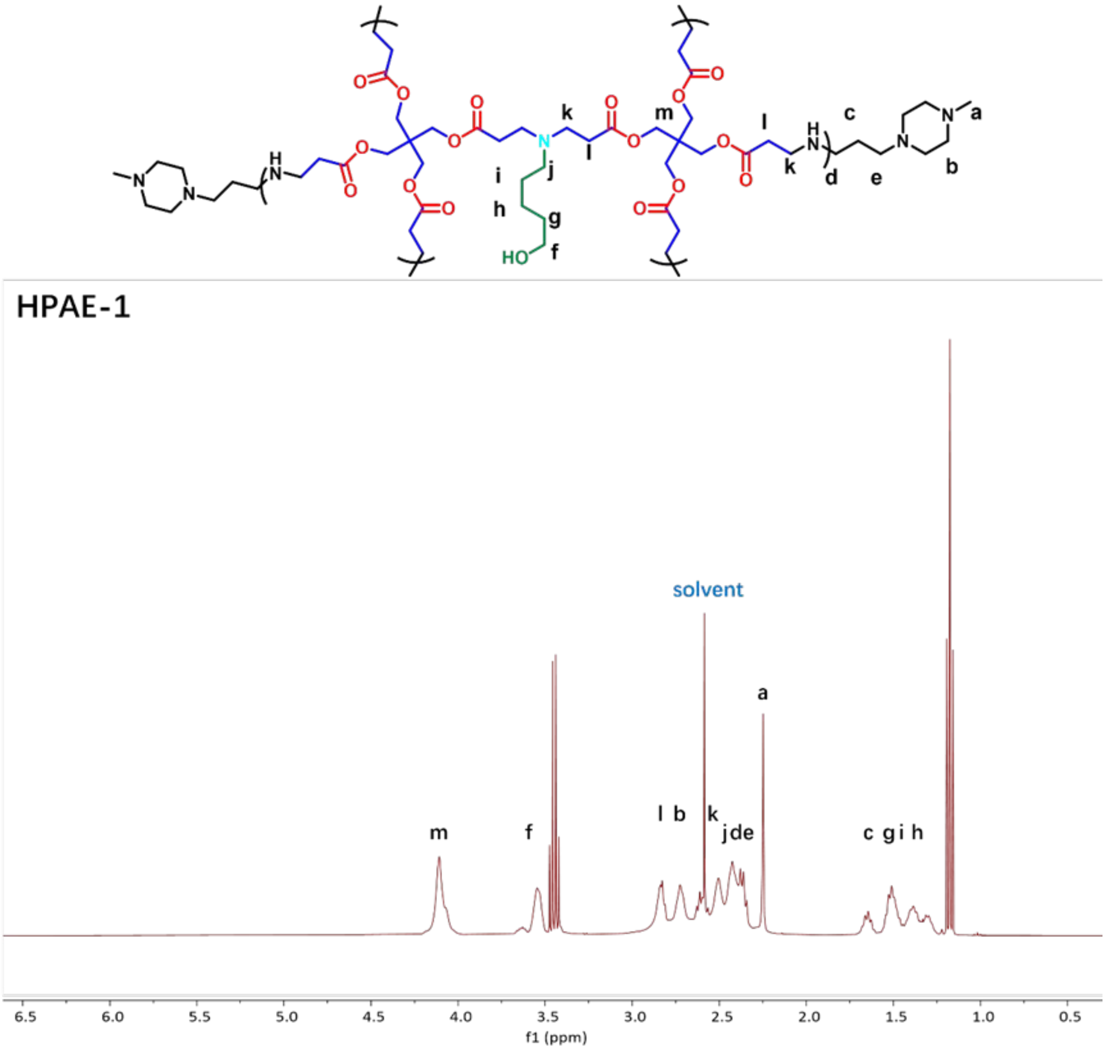

Figure S14. ^{1}H NMR spectrum of HPAE-1 synthesized by the classic method.[9]

Terminal ratio (TR) = [E7] / [PTTA] = $[(I_a) / 3] / [I_m / 8]$, where I_a and I_m stand for the integral intensity of peaks a and m in ^{1}H NMR spectrum.

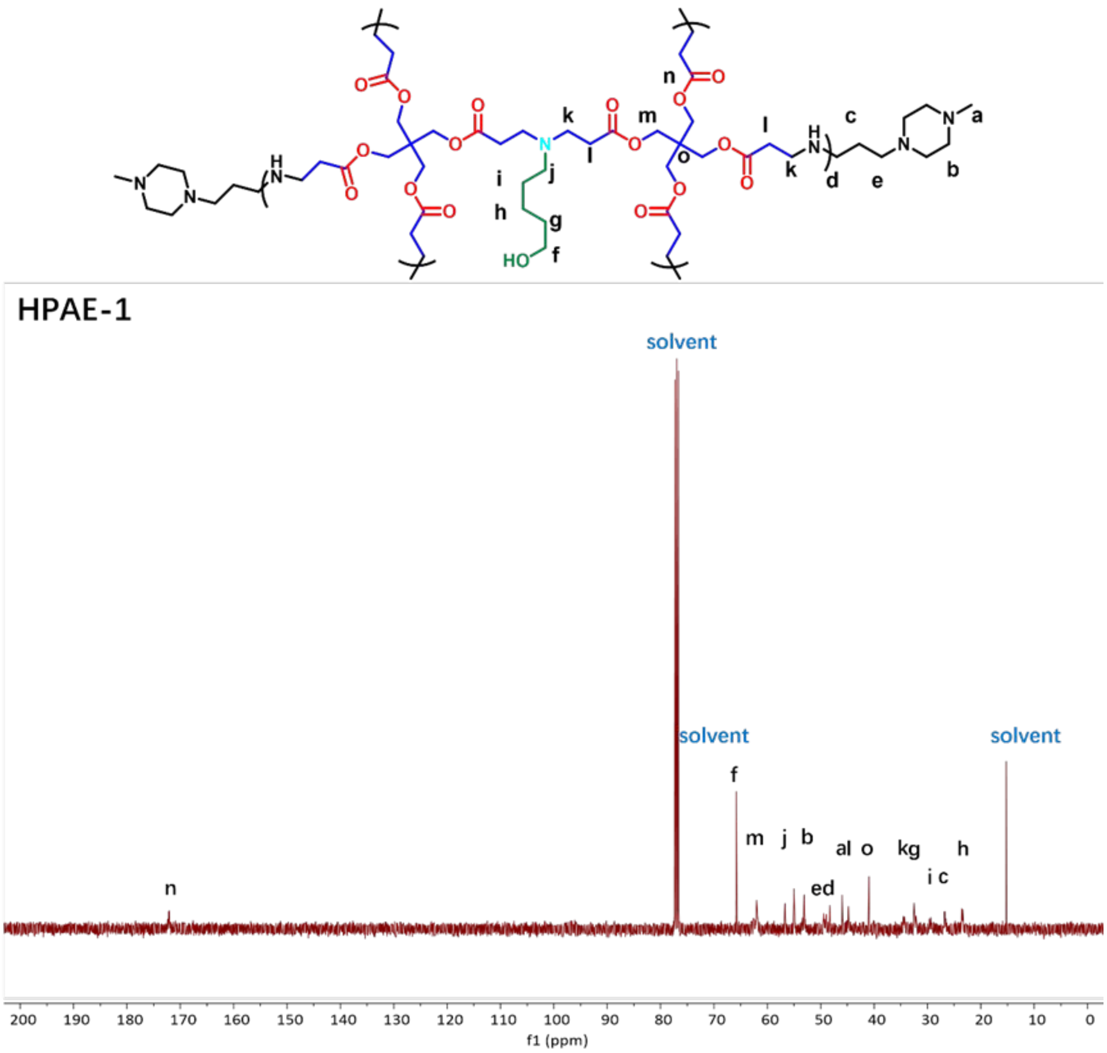

Figure S15. ^{13}C NMR spectrum of HPAE-1 synthesized by the classic method.[9]

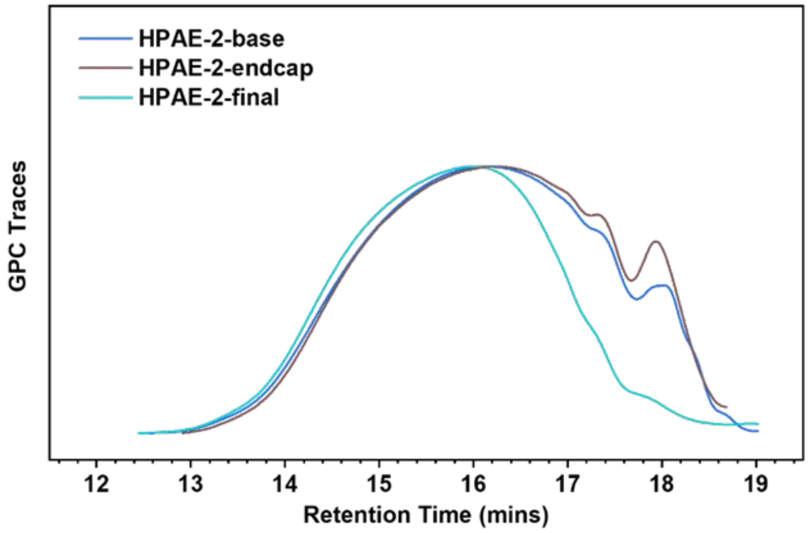

Figure S16. GPC traces of HPAE-2 synthesized as control group.

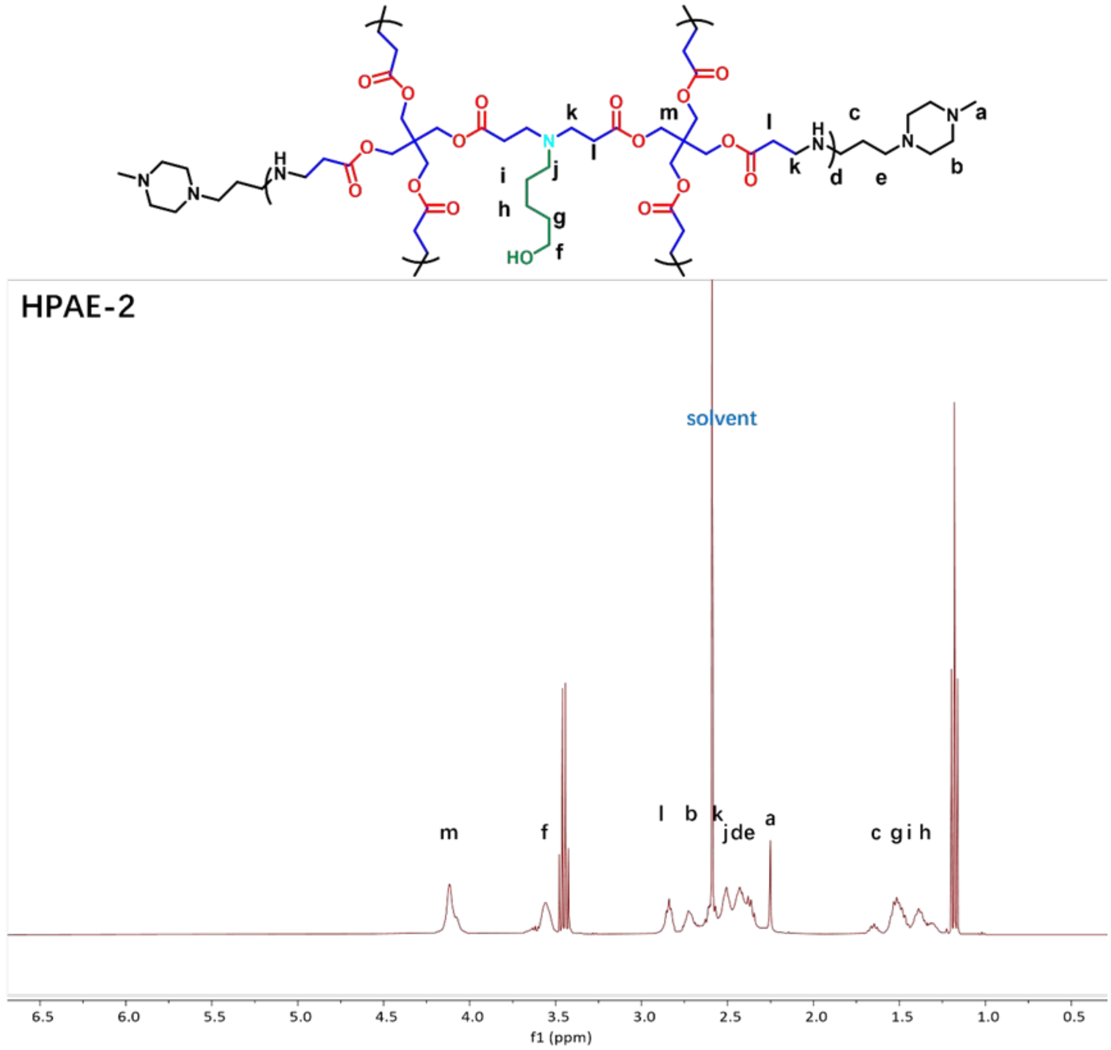

Figure S17. ^{1}H NMR spectrum of HPAE-2 synthesized as control group.

Terminal ratio (TR) = [E7] / [PTTA] = [(I_a) / 3] / [I_m / 8], where I_a and I_m stand for the integral intensity of peaks a and m in ^{1}H NMR spectrum.

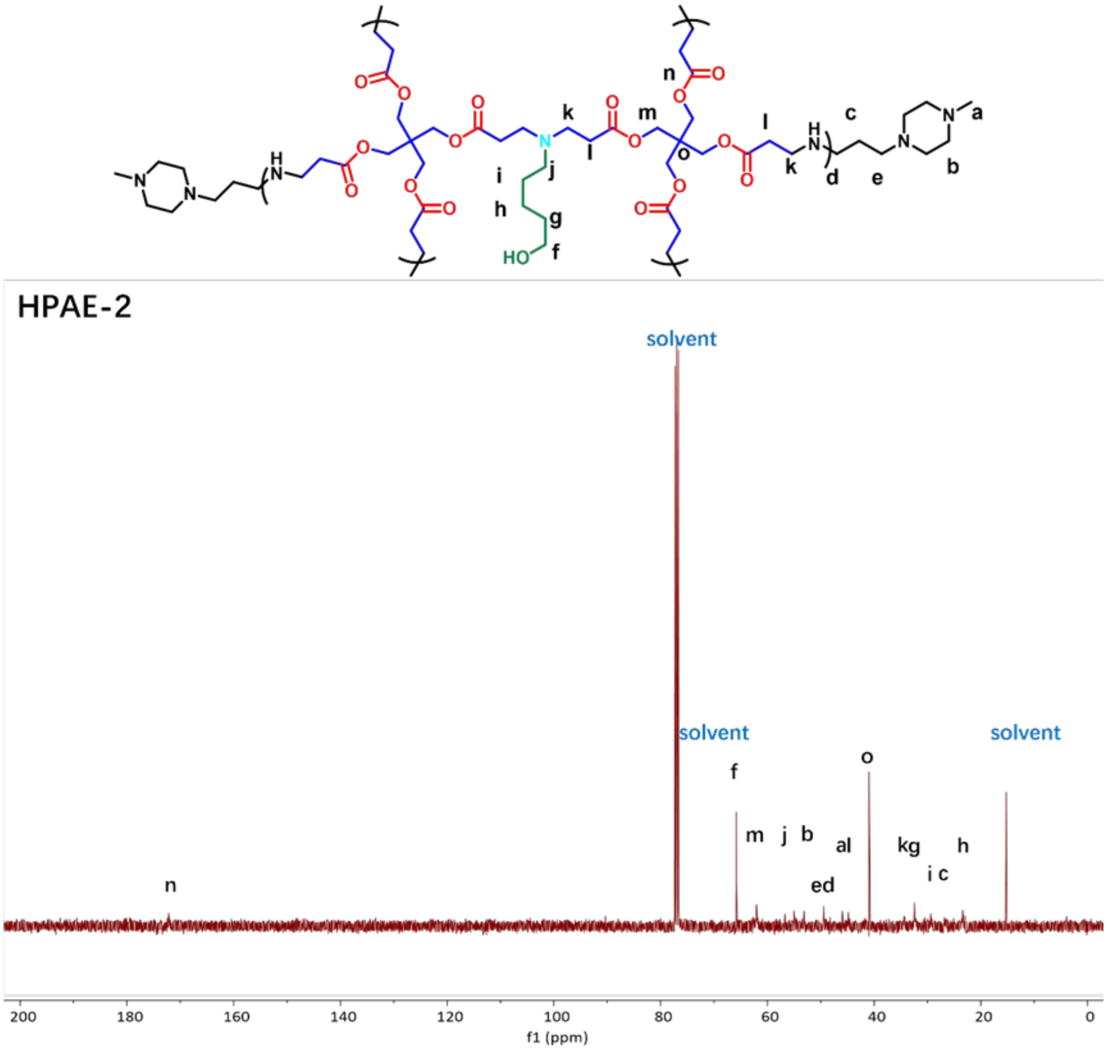

Figure S18. ^{13}C NMR spectrum of HPAE-2 synthesized as control group.

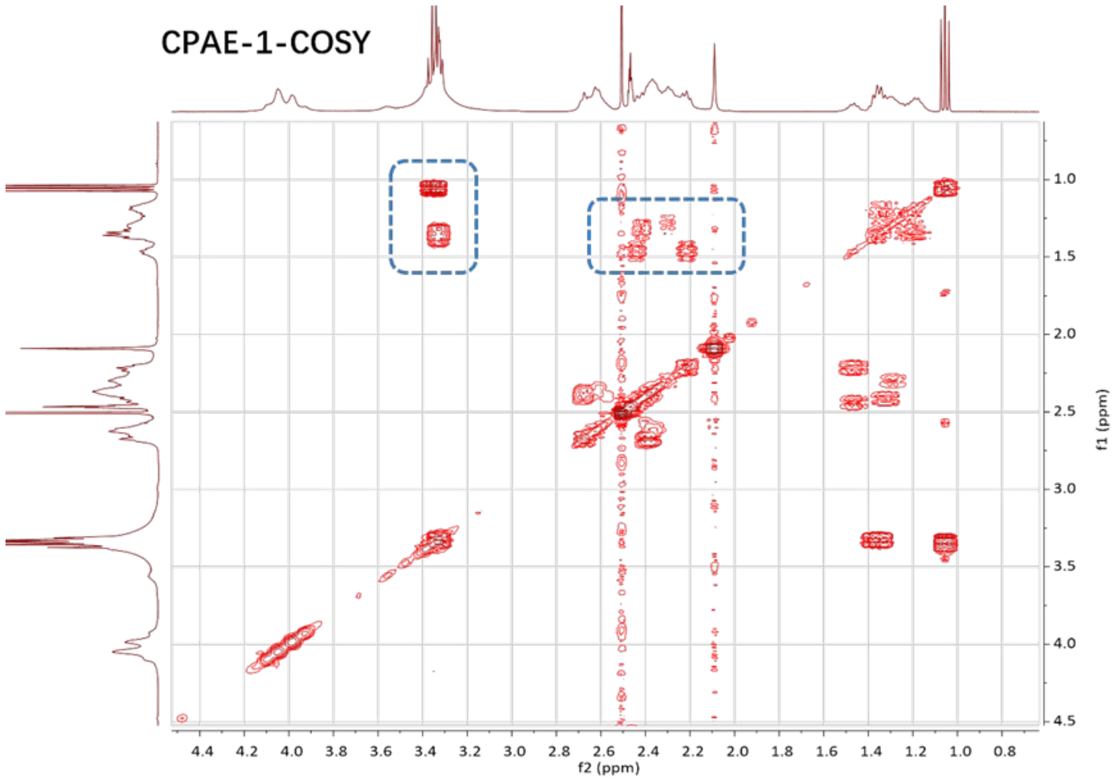

Figure S19. ^{1}H, ^{1}H -COSY spectrum of CPAE-1.

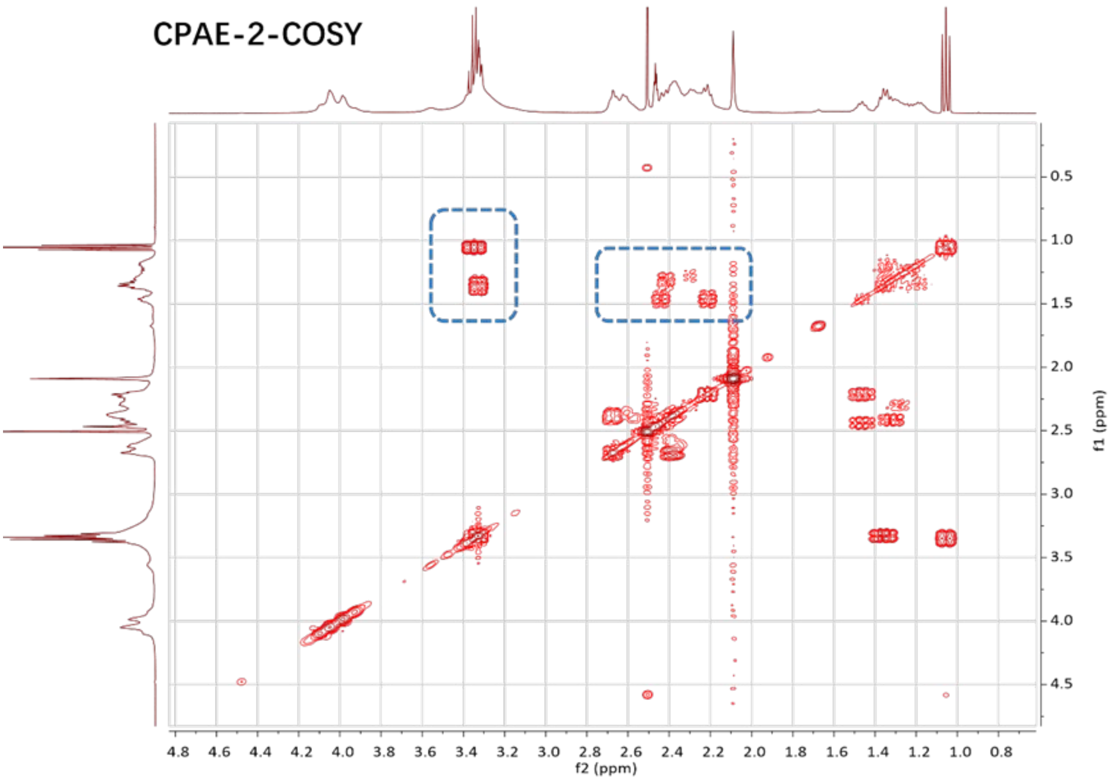

Figure S20. ^{1}H, ^{1}H -COSY spectrum of CPAE-2.

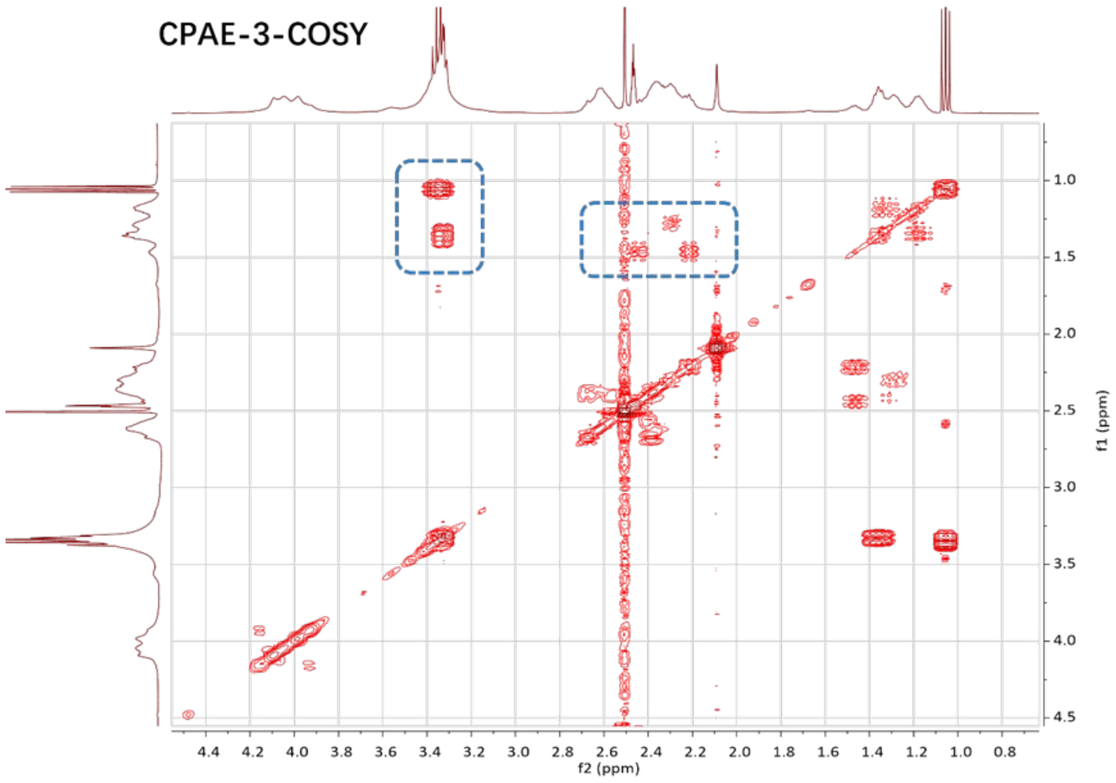

Figure S21. ^{1}H, ^{1}H -COSY spectrum of CPAE-3.

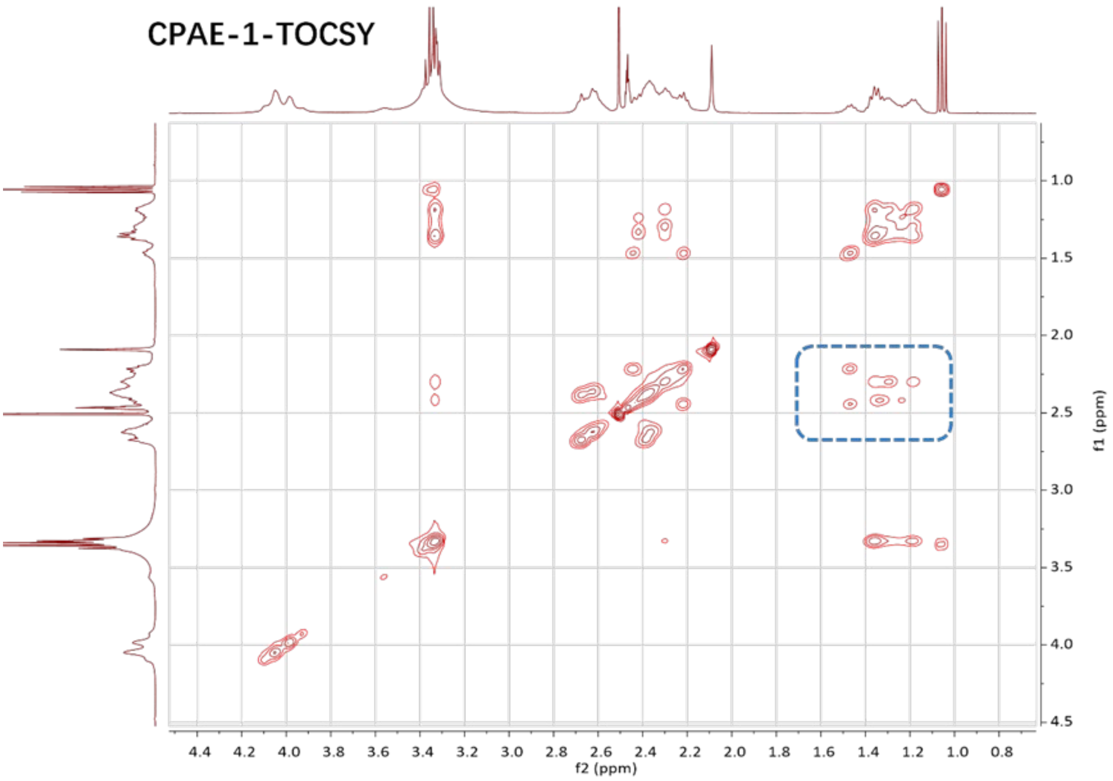

Figure S22. ^{1}H, ^{1}H -TOCSY spectrum of CPAE-1.

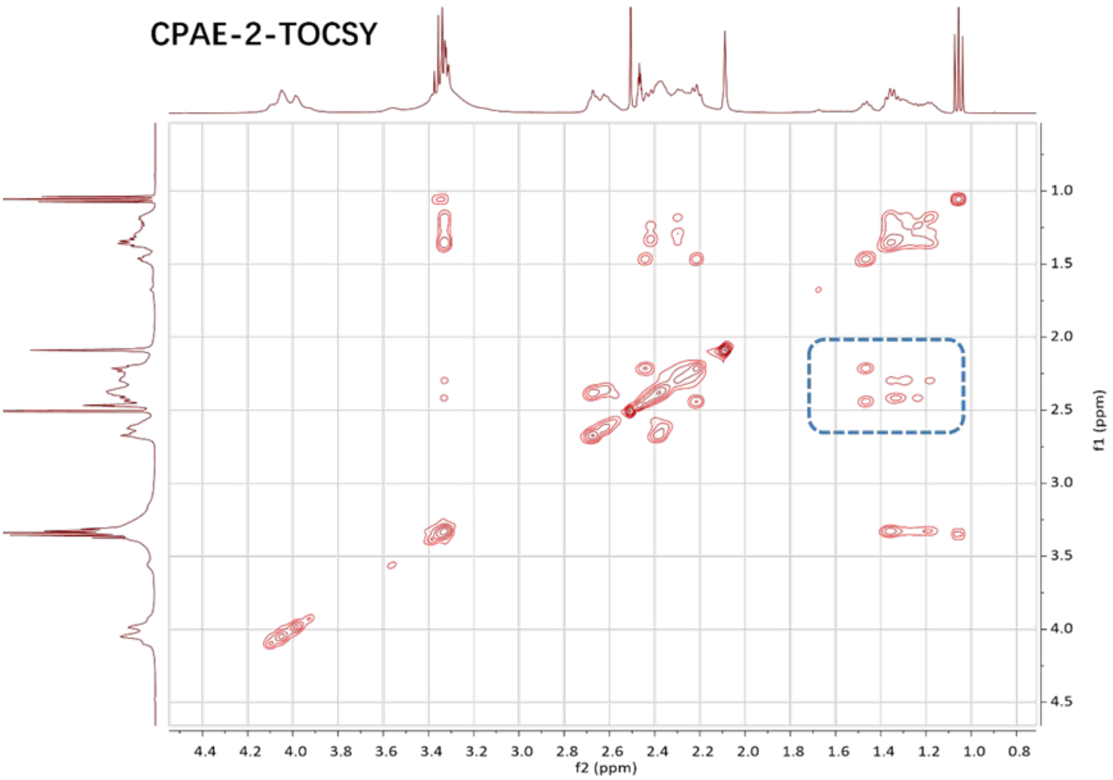

Figure S23. ^{1}H, ^{1}H -TOCSY spectrum of CPAE-2.

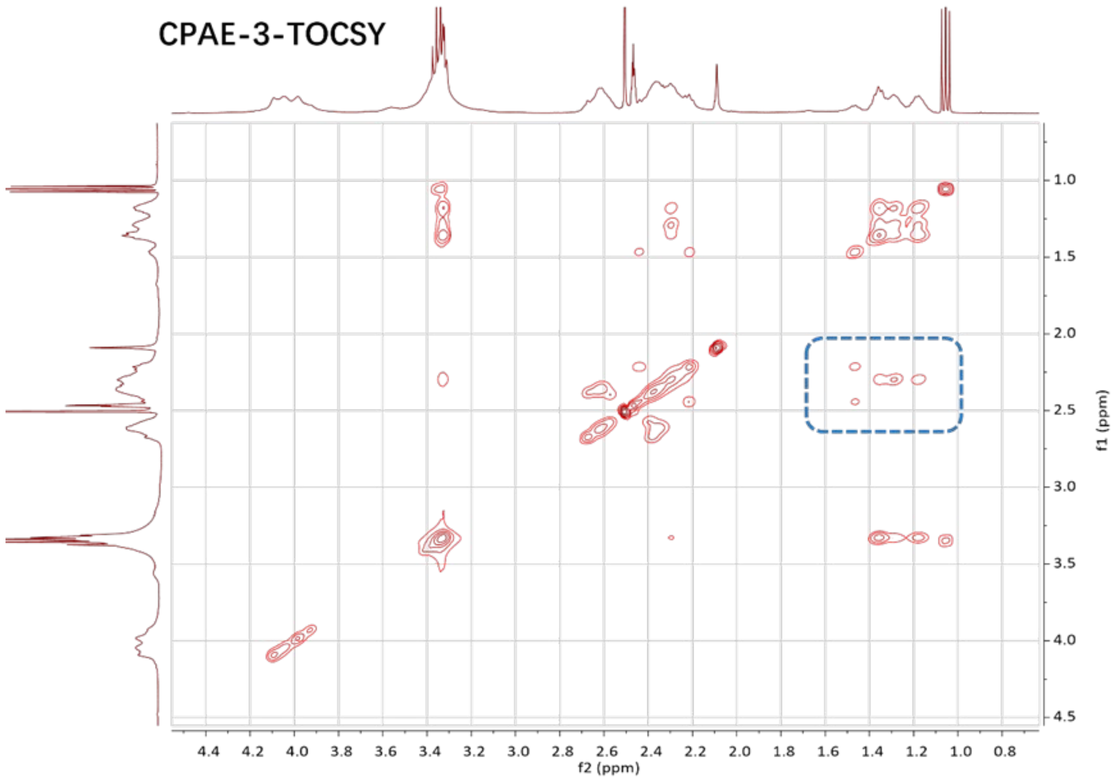

Figure S24. ^{1}H, ^{1}H -TOCSY spectrum of CPAE-3.

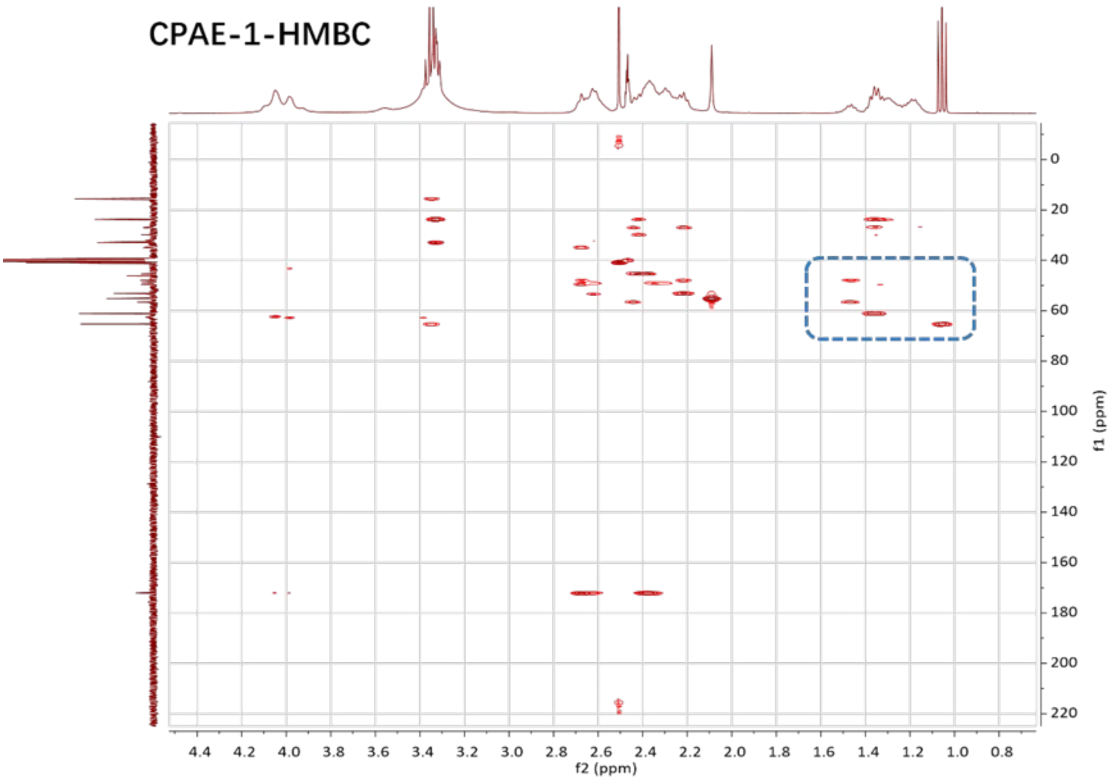

Figure S25. ^{13}C, ^{1}H -HMBC spectrum of CPAE-1.

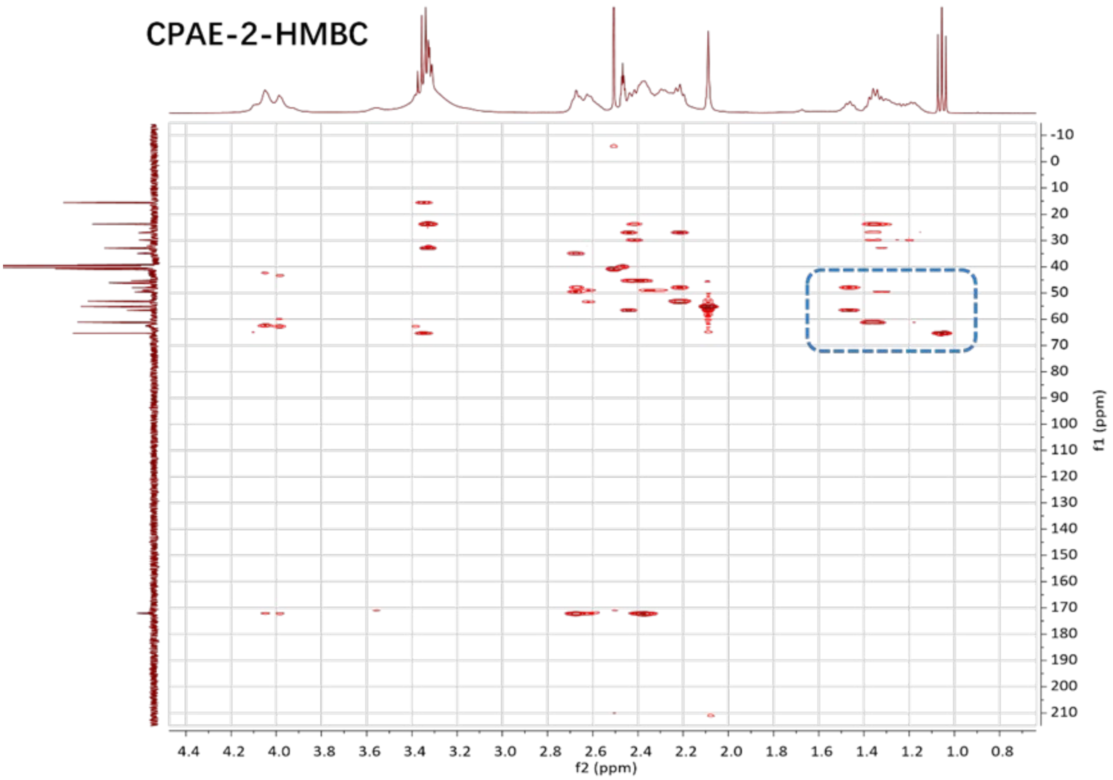

Figure S26. ^{13}C, ^{1}H -HMBC spectrum of CPAE-2.

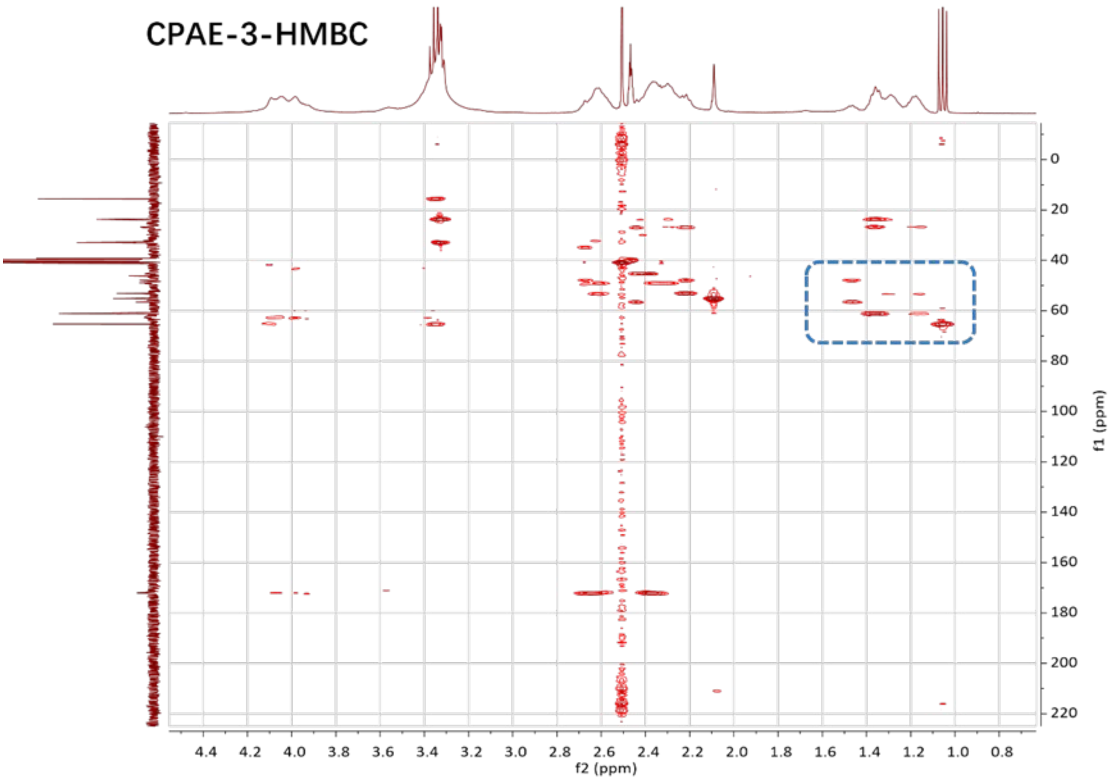

Figure S27. ^{13}C, ^{1}H -HMBC spectrum of CPAE-3.

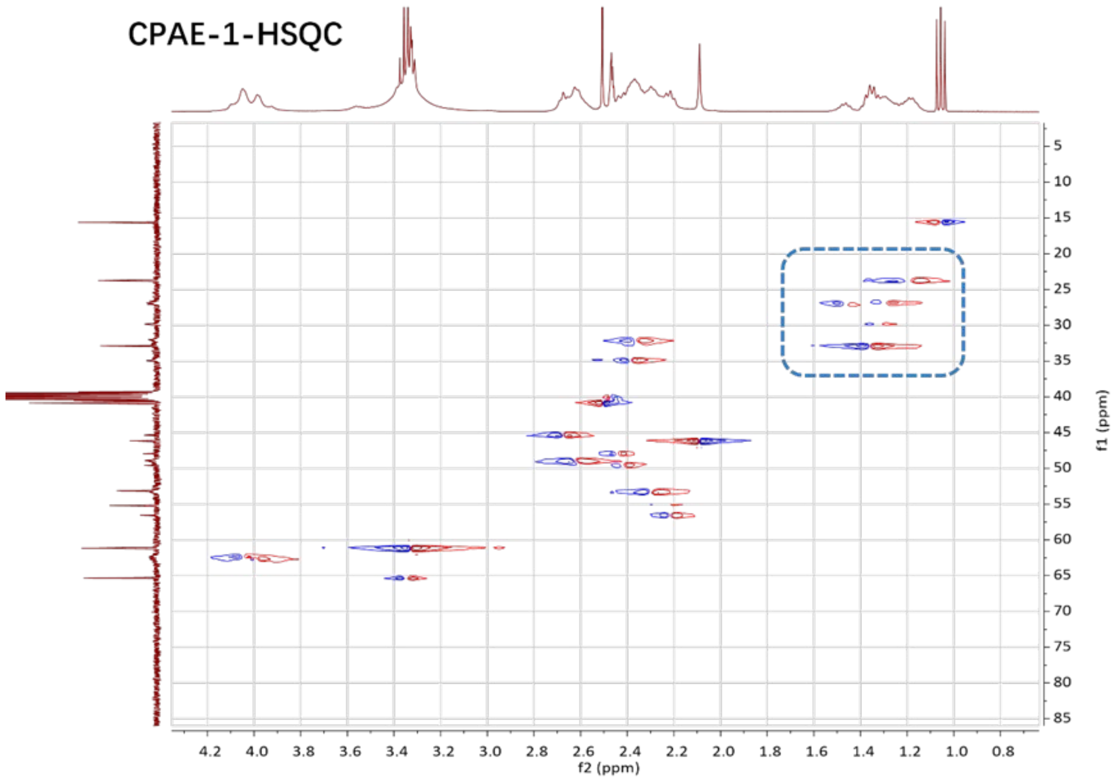

Figure S28. ^{13}C, ^{1}H -HSQC spectrum of CPAE-1.

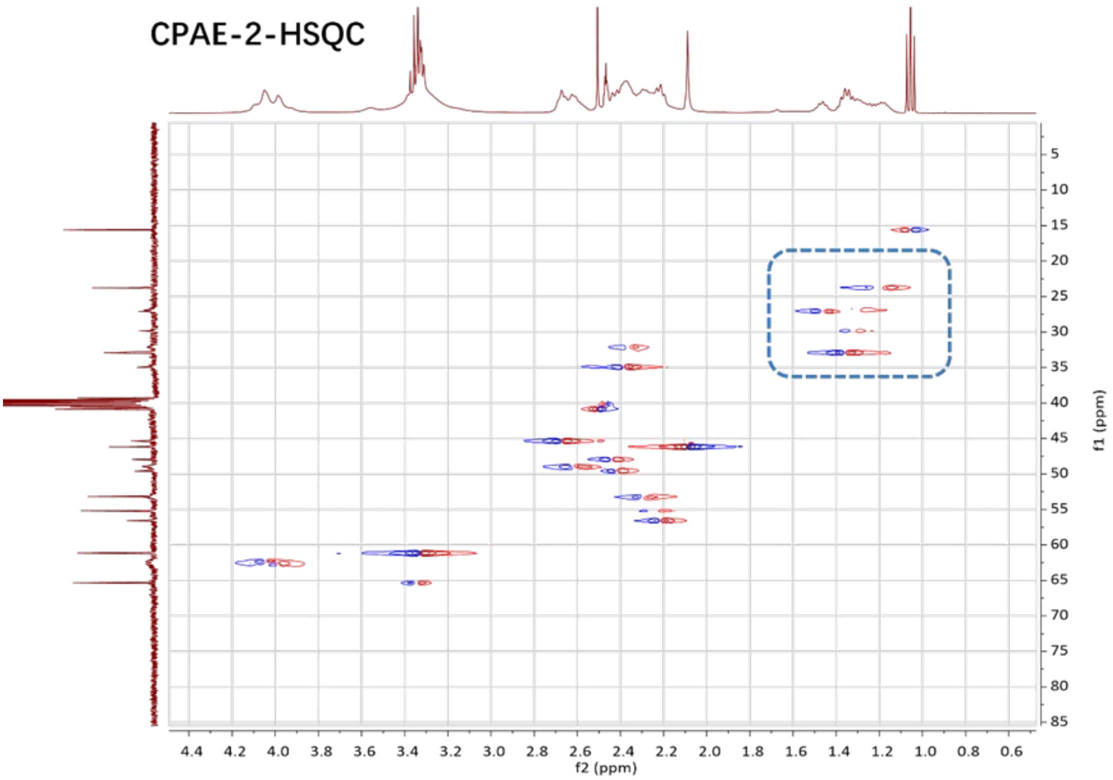

Figure S29. ^{13}C, ^{1}H -HSQC spectrum of CPAE-2.

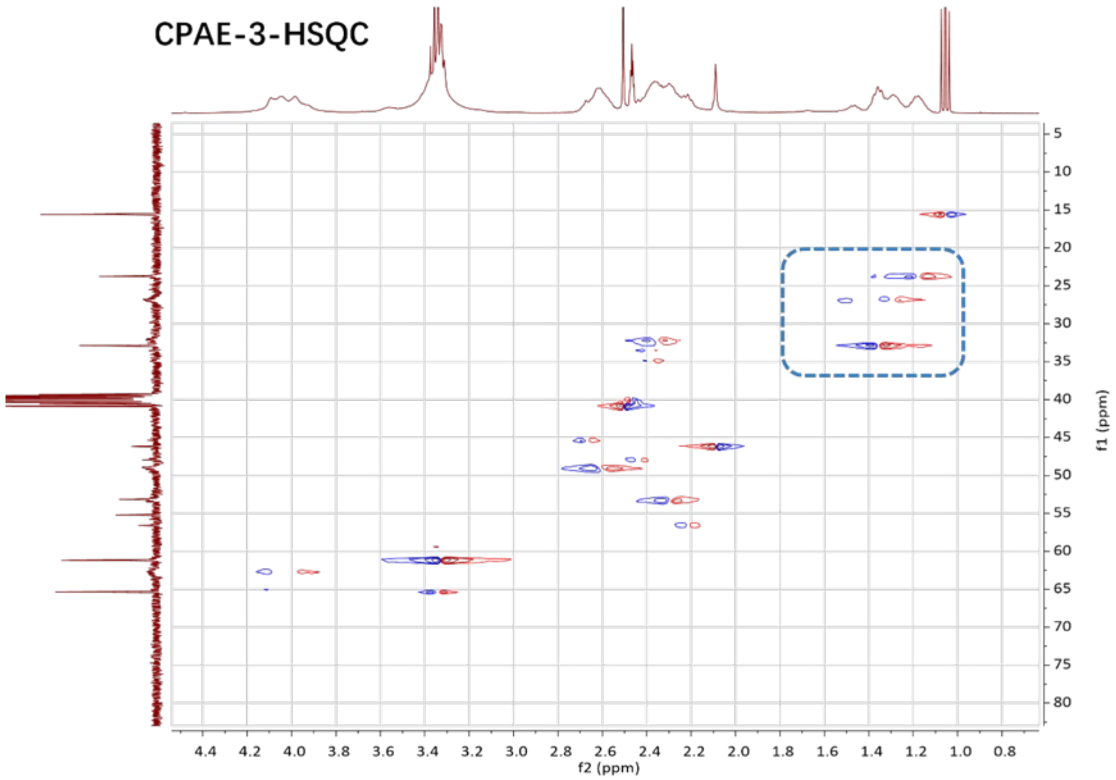

Figure S30. ^{13}C, ^{1}H -HSQC spectrum of CPAE-3.

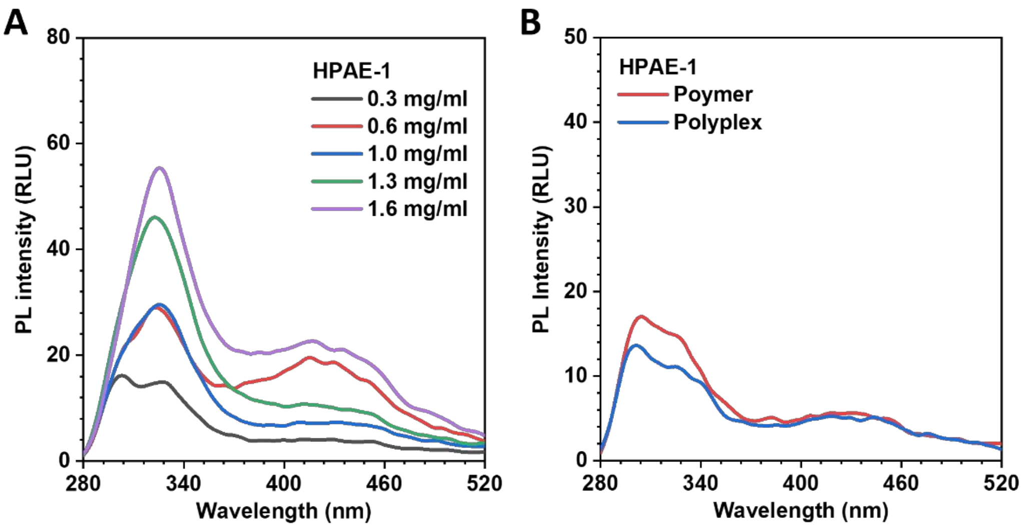

Figure S31. (A) Different photoluminescence behaviors in relative light units (RLU) of HPAE-1 at different CPAE concentrations (in water). (B) The photoluminescence behaviors of HPAE-1 and its polyplex in 25 mM sodium acetate (pH=4.8).

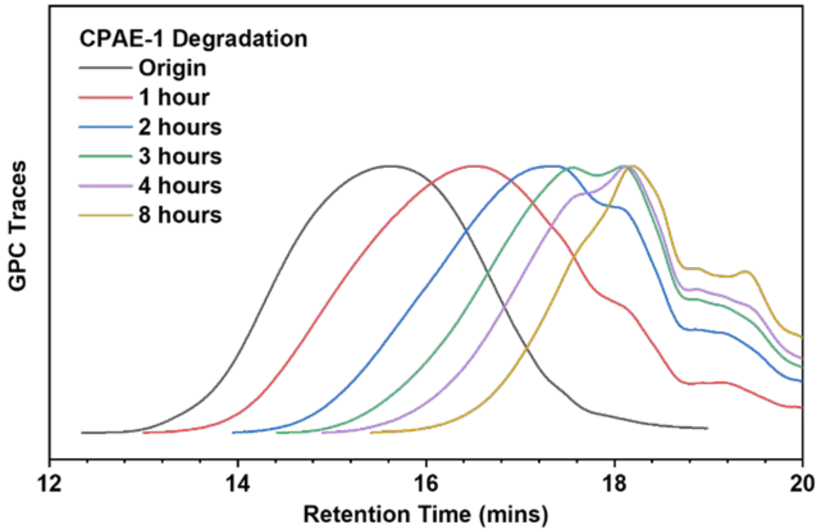

Figure S32. GPC traces of CPAE-1 at 37 ℃ in sodium acetate.

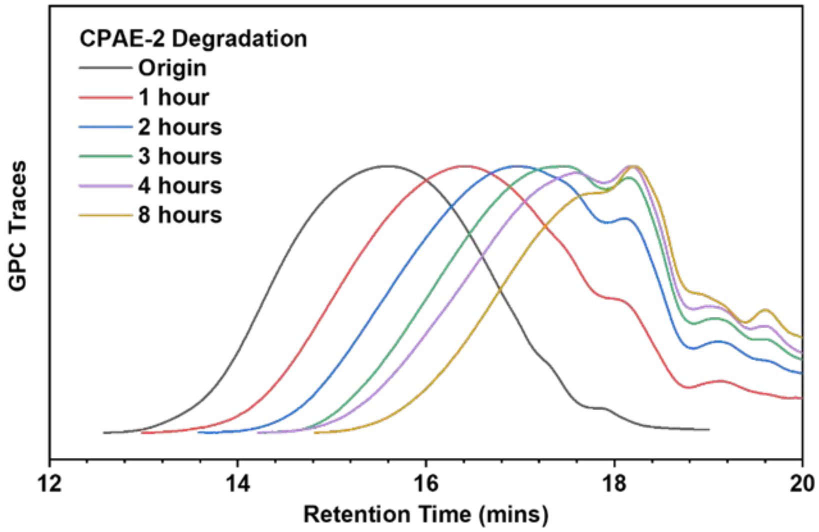

Figure S33. GPC traces of CPAE-2 at 37 ℃ in sodium acetate.

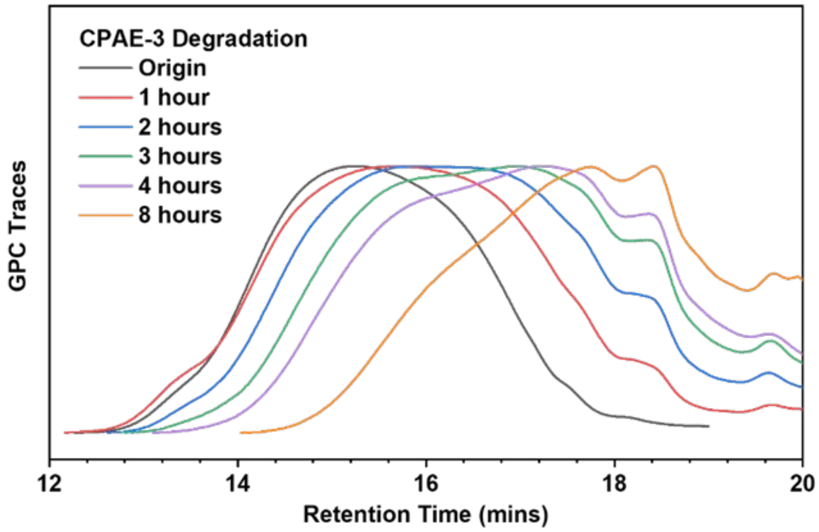

Figure S34. GPC traces of CPAE-3 at 37 ℃ in sodium acetate.

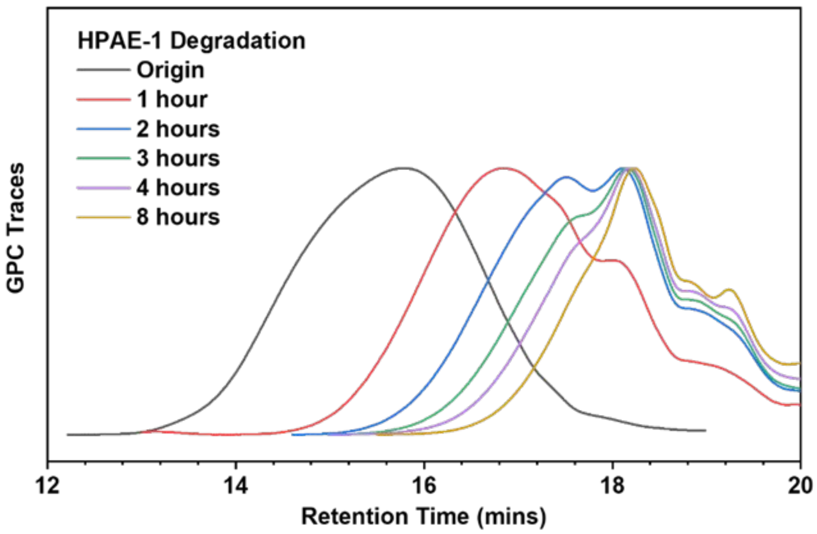

Figure S35. GPC traces of HPAE-1 at 37 ℃ in sodium acetate.

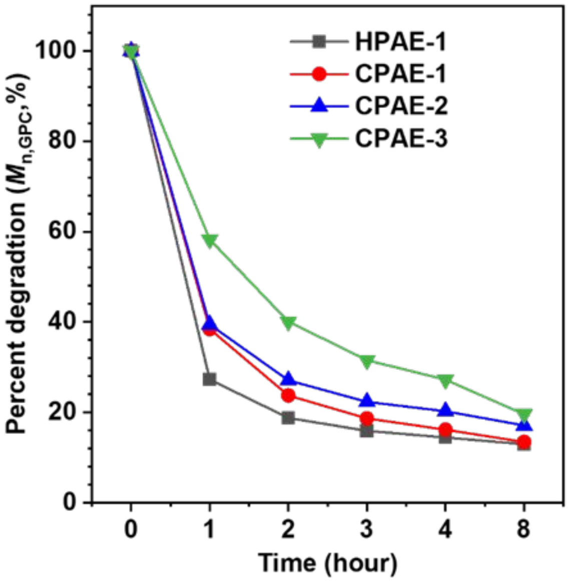

Figure S36. Polymer degradation at 37 ℃ in sodium acetate buffer. Degradation is expressed as percent degradation of polymer number average molecular weight ($M_{n,GPC,}$ Table S1) over time based on GPC data.

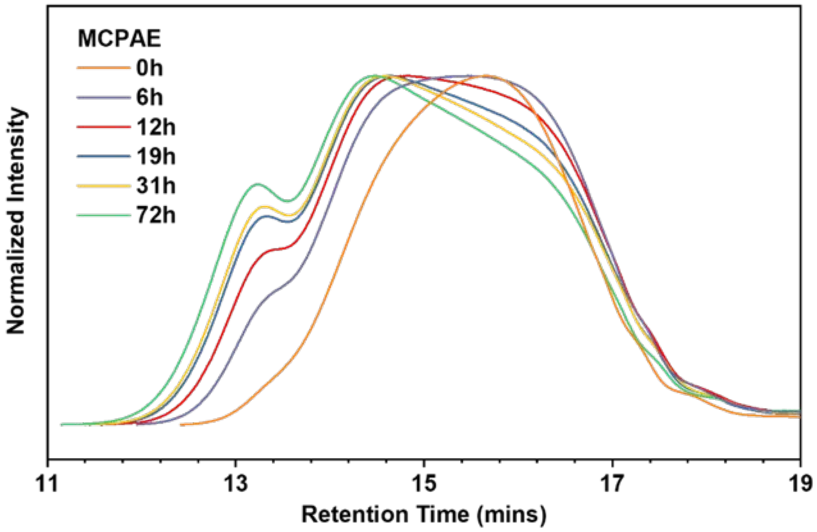

Figure S37. GPC traces of MCPAE-0h to -72h with different cyclization extent synthesized by Method 3.

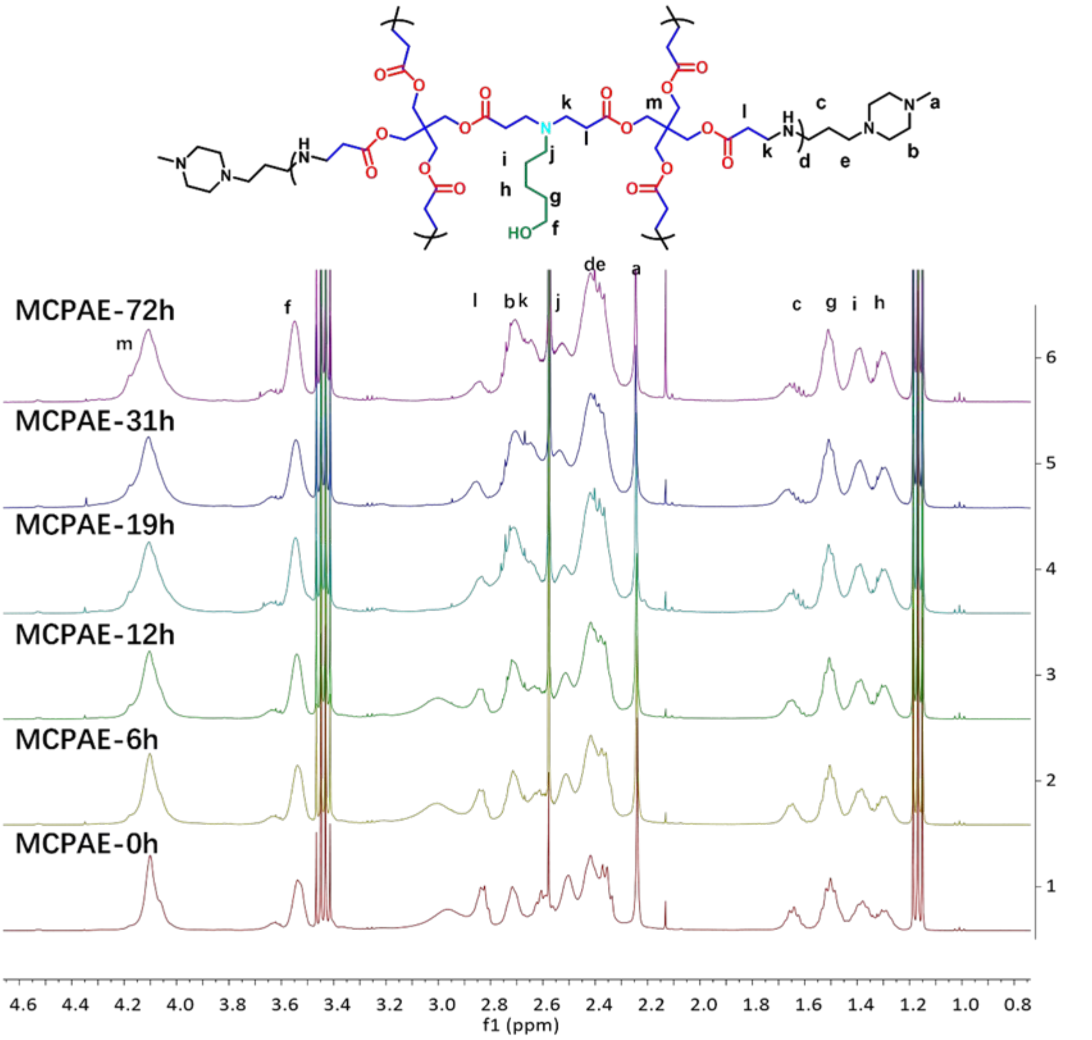

Figure S38. ^{1}H NMR spectra of MCPAE-0h to -72h with different cyclization extent synthesized by Method 3.

Terminal ratio (TR) = [E7] / [PTTA] = $[(I_a) / 3] / [I_m / 8]$, where I_a and I_m stand for the integral intensity of peaks a and m in ^{1}H NMR spectra.

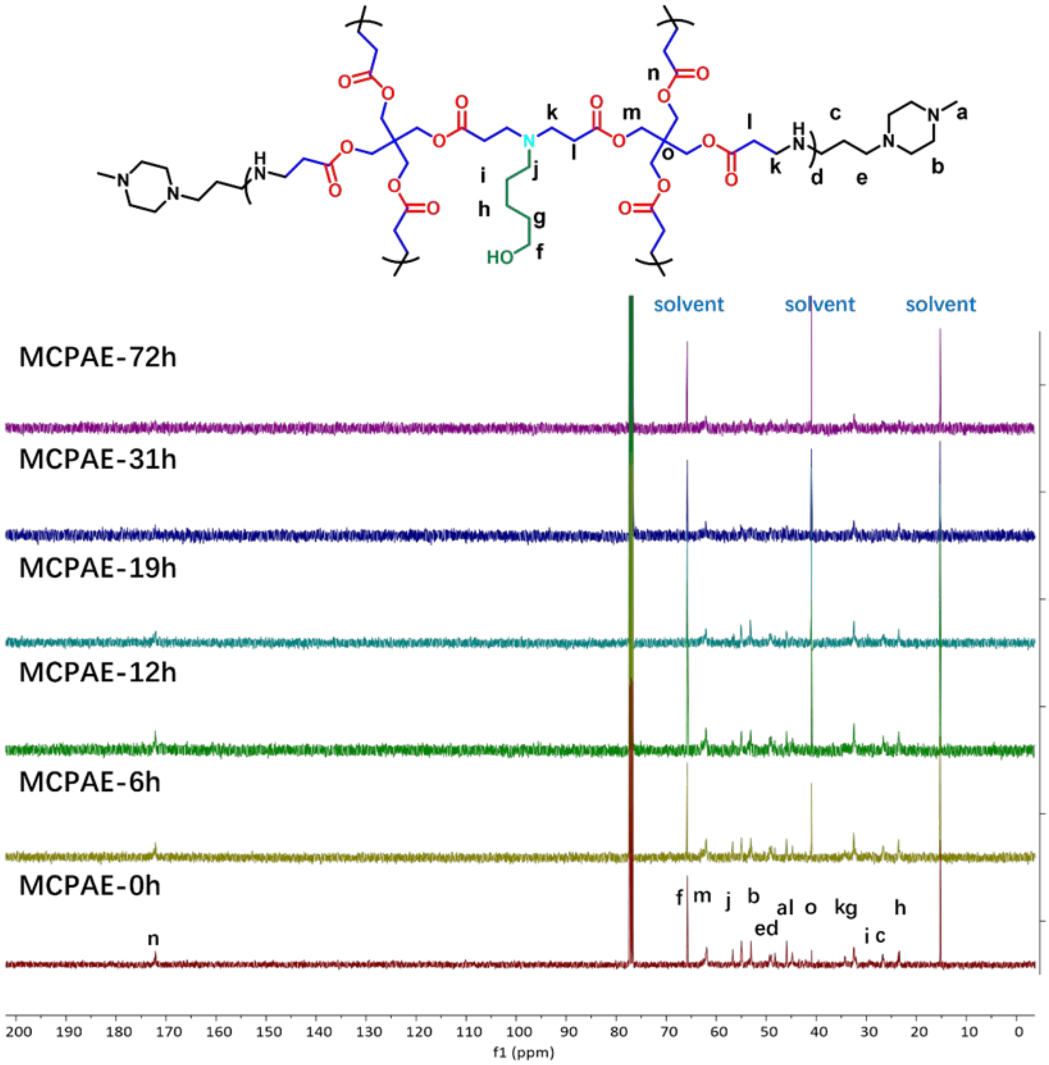

Figure S39. ^{13}C NMR spectra of MCPAE-0h to -72h with different cyclization extent synthesized by Method 3.

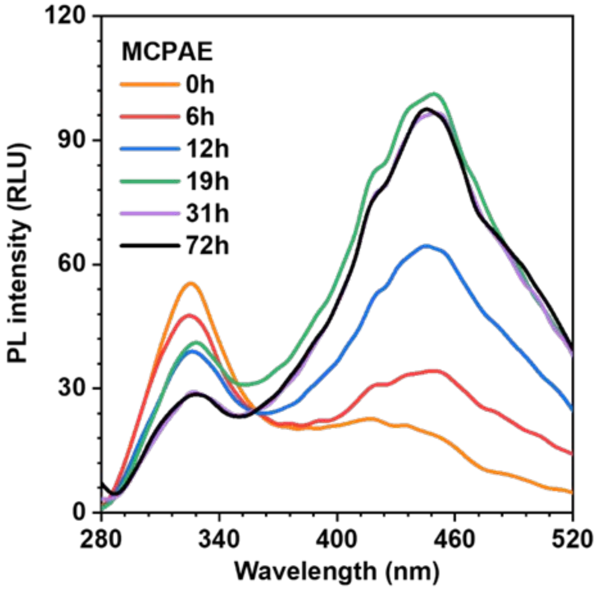

Figure S40. The photoluminescence behaviors of MCPAE-0h to -72h in water.

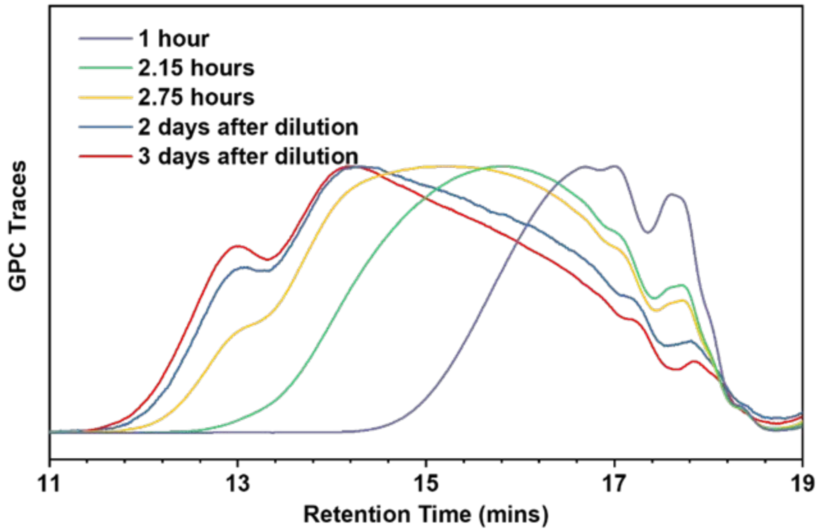

Figure S41. GPC traces of HPAE and MCPAE synthesized for terminal groups optimization. HPAE obtained at 2.75 hours was used as the base polymer for screening and optimization of different terminal groups. MCPAEs obtained at 3 days after dilution was used as the base polymer for screening and optimization of different terminal groups.

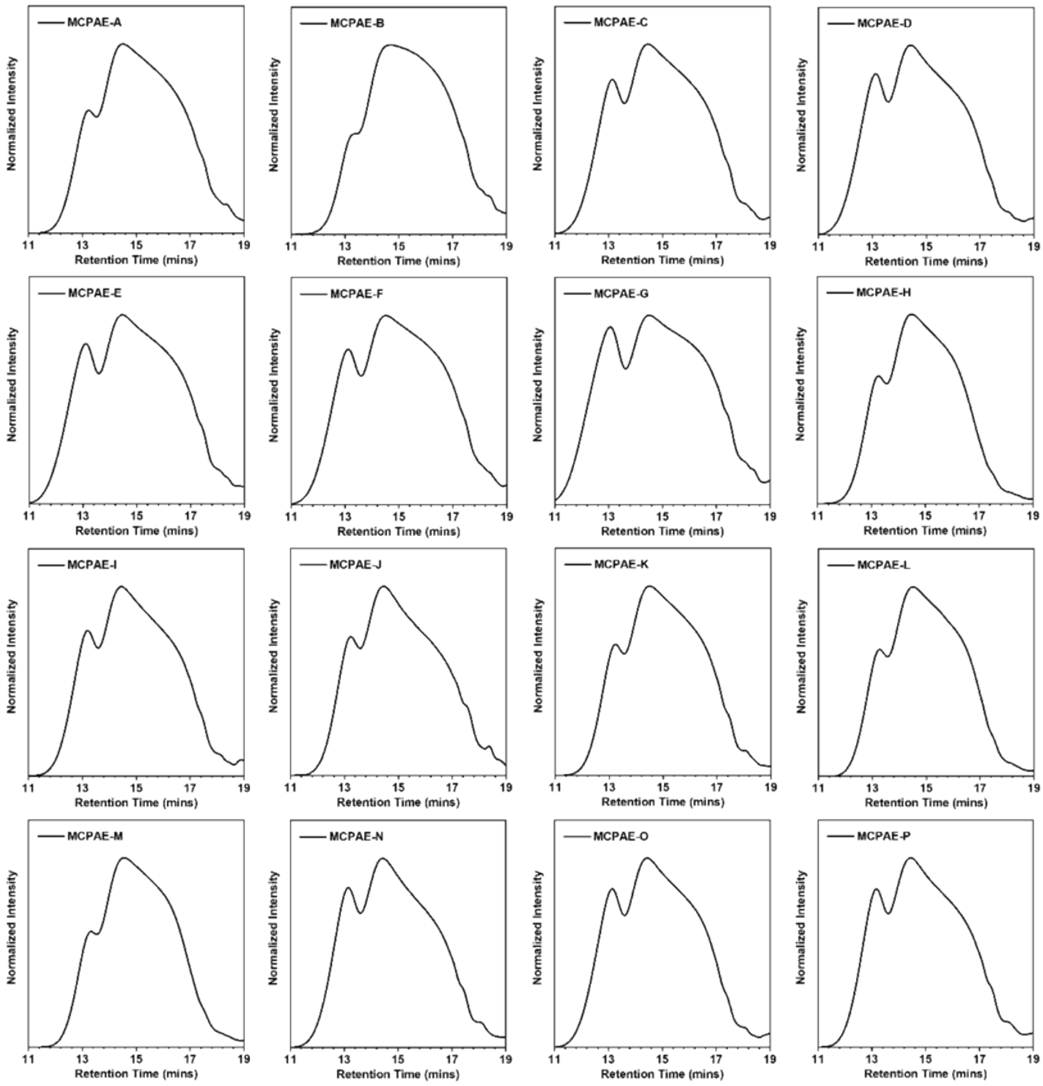

Figure S42. GPC traces of the MCPAE-A to -P synthesized by Method 3.

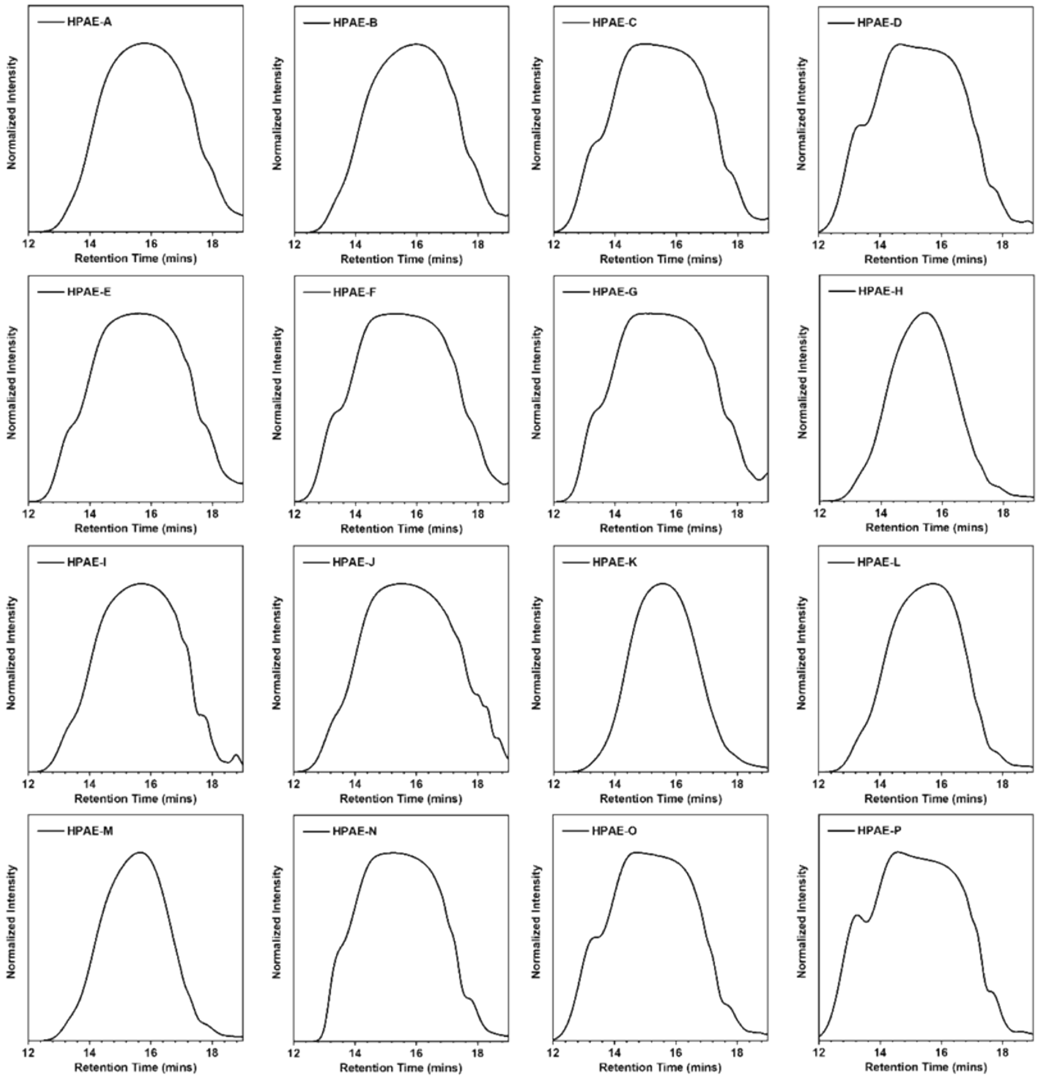

Figure S43. GPC traces of the HPAE-A to -P synthesized by Method 3.

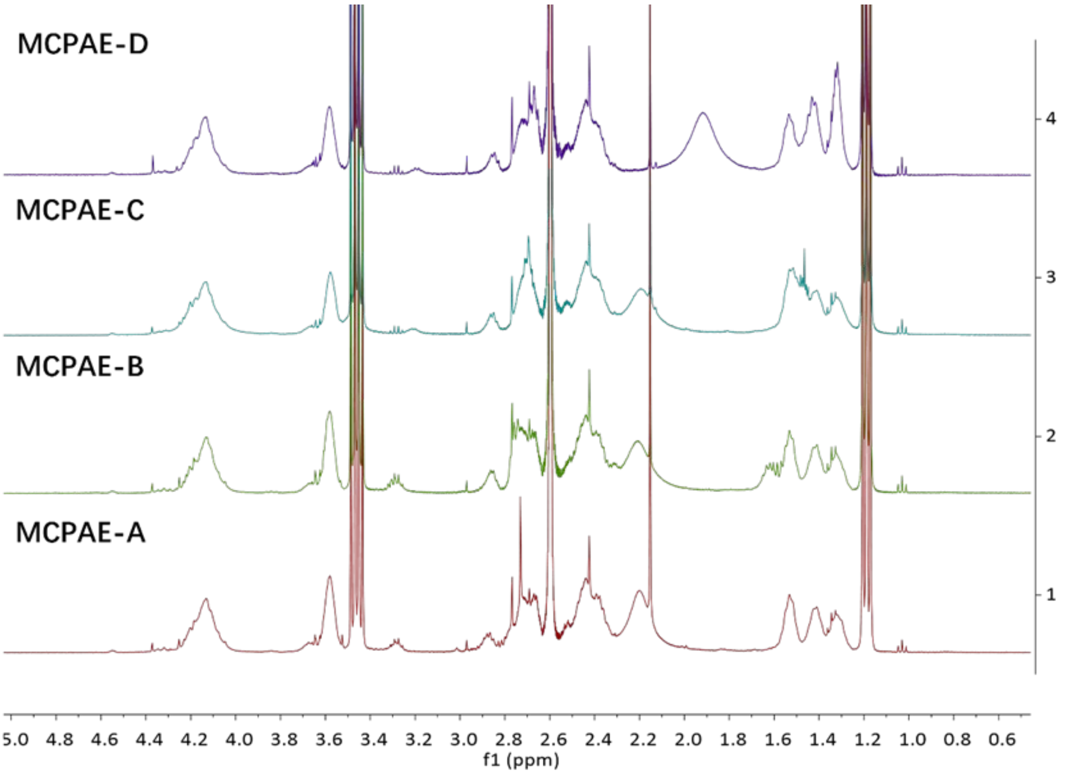

Figure S44. ^{1}H NMR spectra of MCPAE-A to -D synthesized by Method 3.

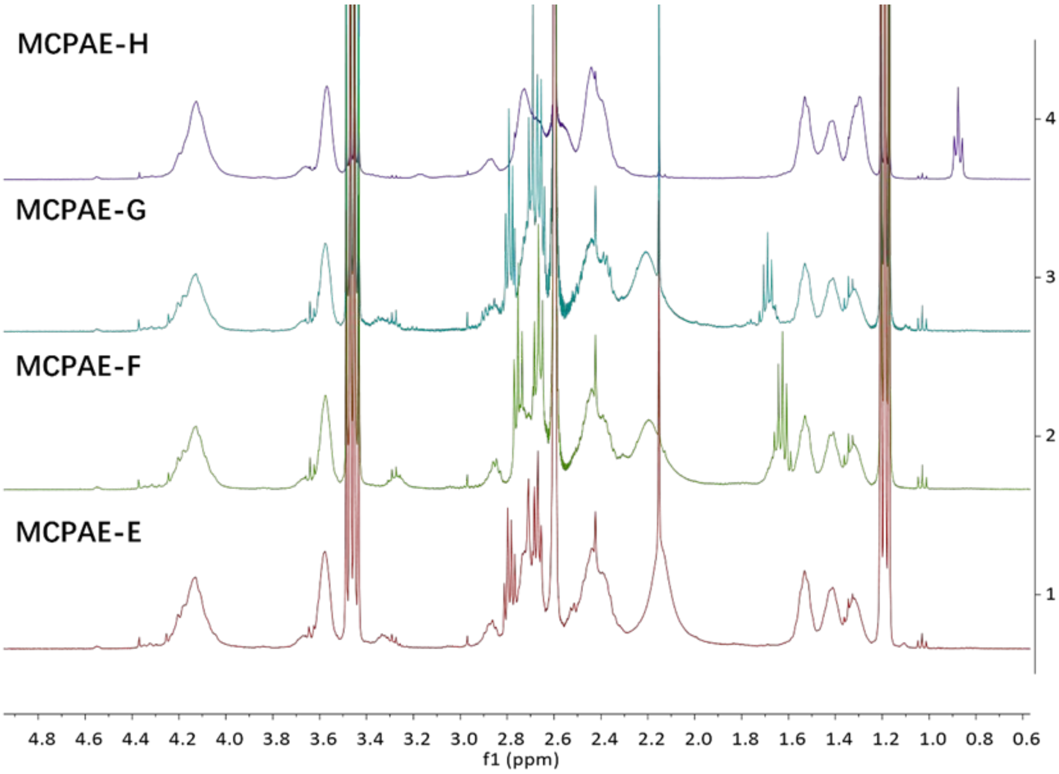

Figure S45. ^1H NMR spectra of MCPAE-E to -H synthesized by Method 3.

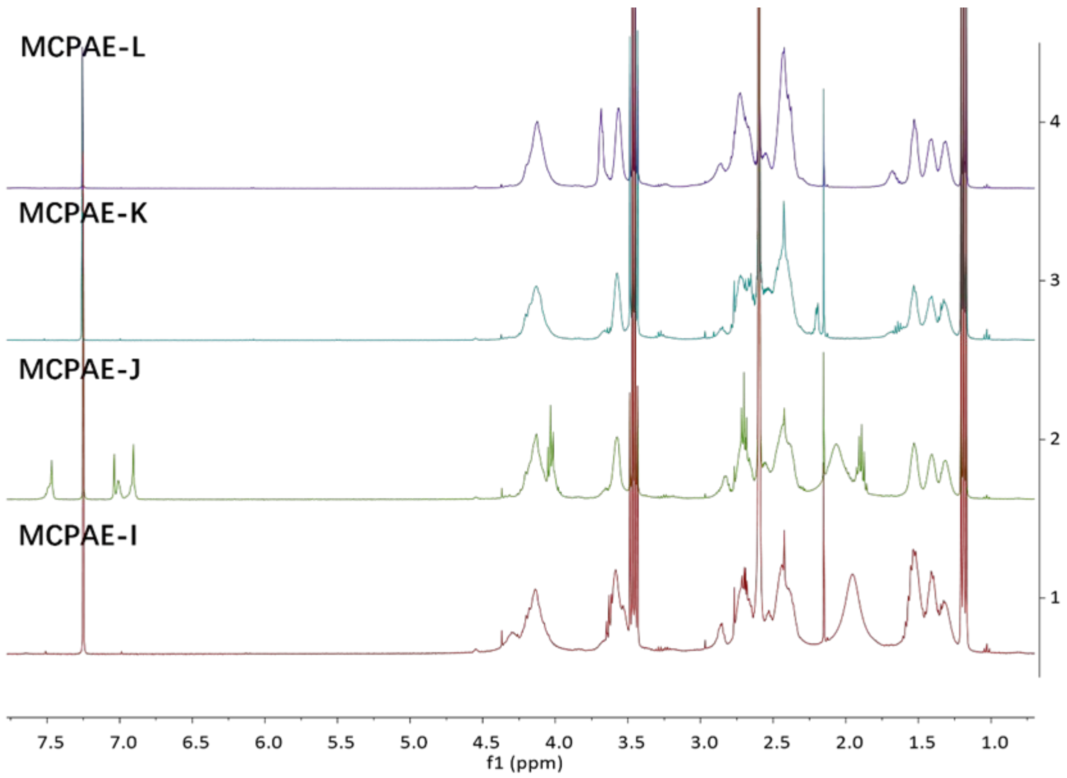

Figure S46. ^{1}H NMR spectra of MCPAE-I to -L synthesized by Method 3.

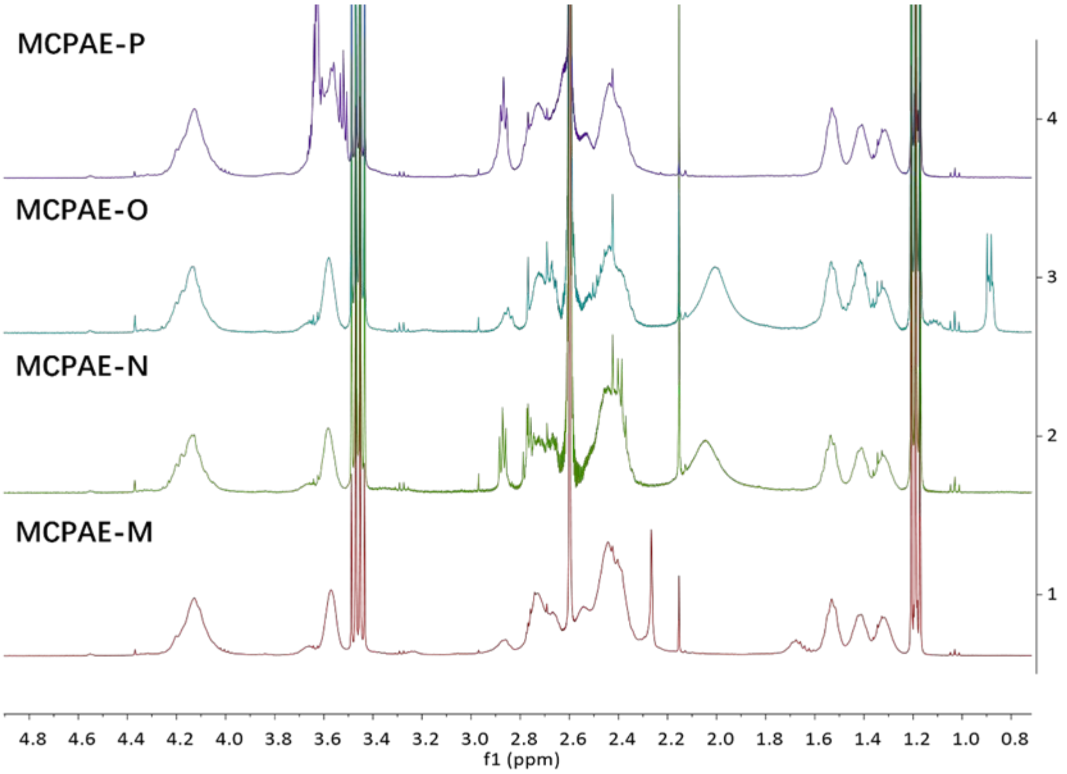

Figure S47. ^{1}H NMR spectra of MCPAE-M to -P synthesized by Method 3.

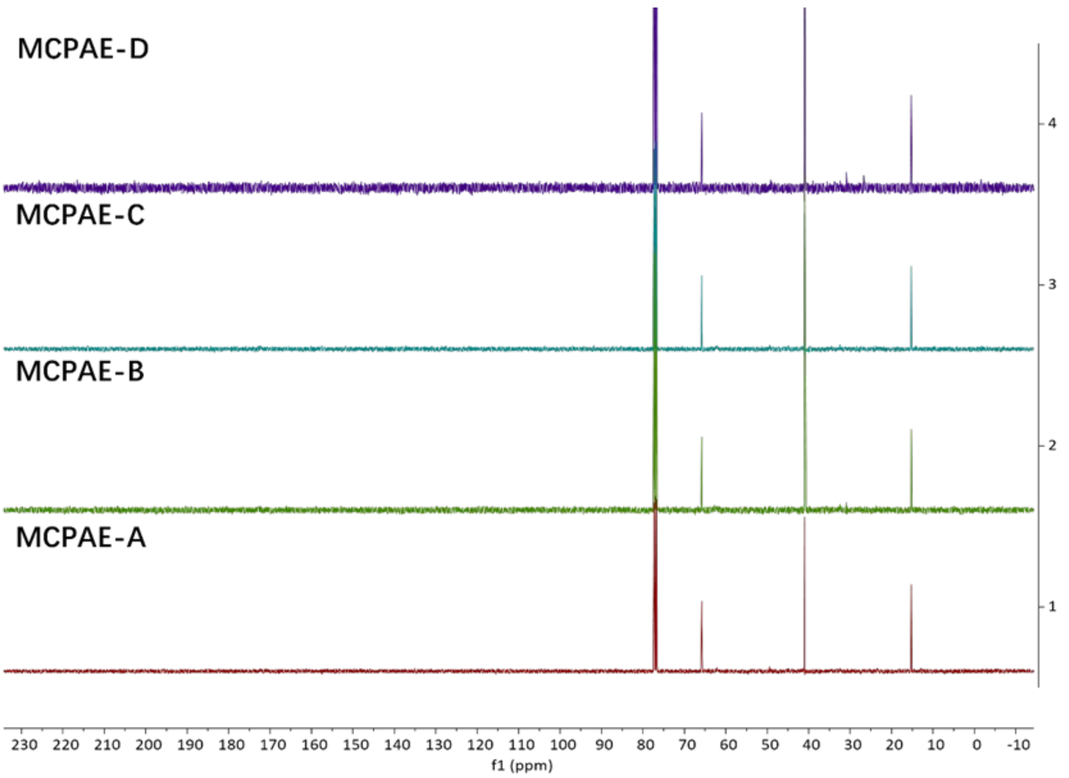

Figure S48. ^{13}C NMR spectra of MCPAE-A to -D synthesized by Method 3.

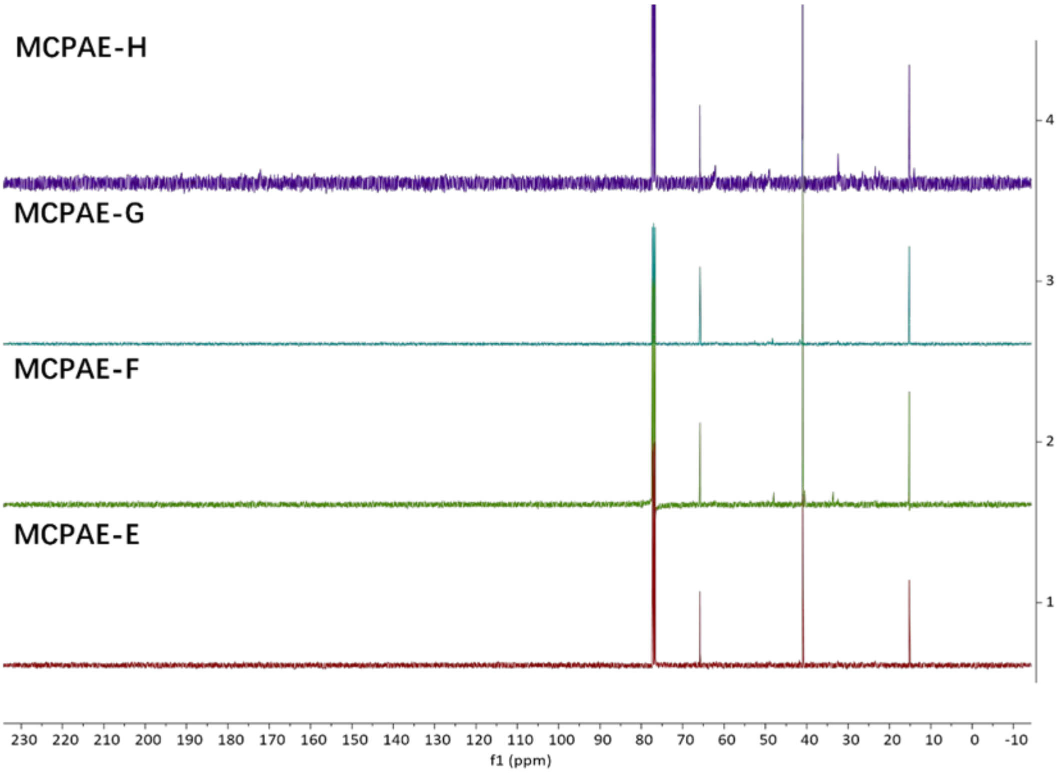

Figure S49. ^{13}C NMR spectra of MCPAE-E to -H synthesized by Method 3.

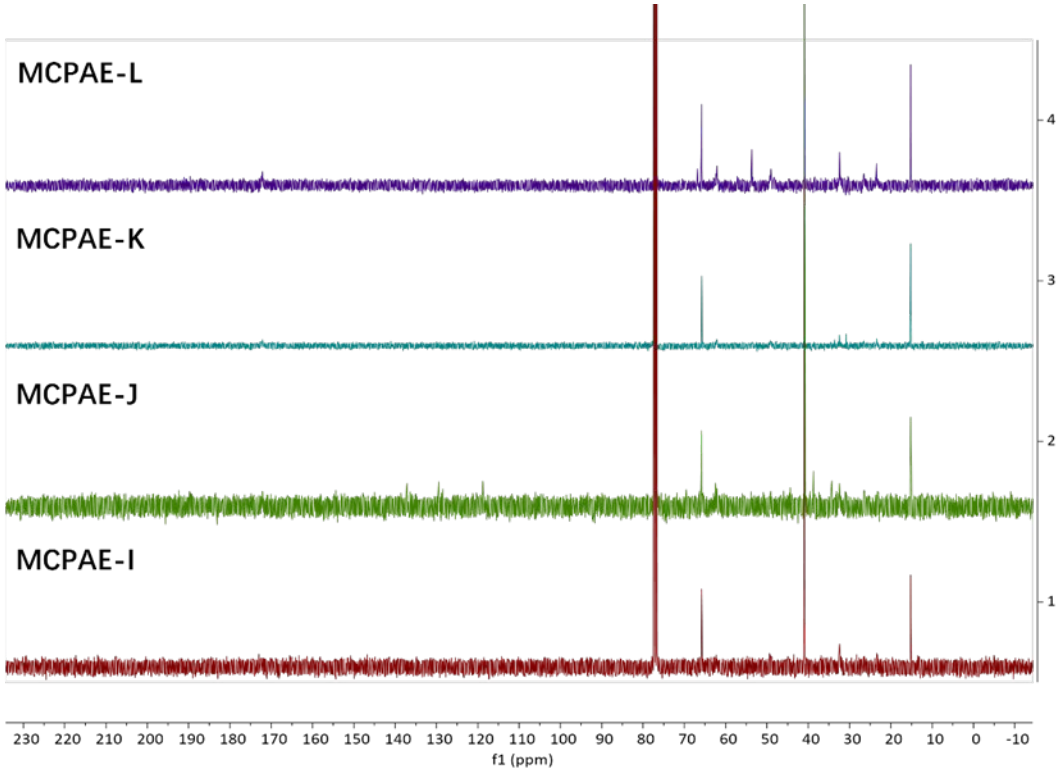

Figure S50. ^{13}C NMR spectra of MCPAE-I to -L synthesized by Method 3.

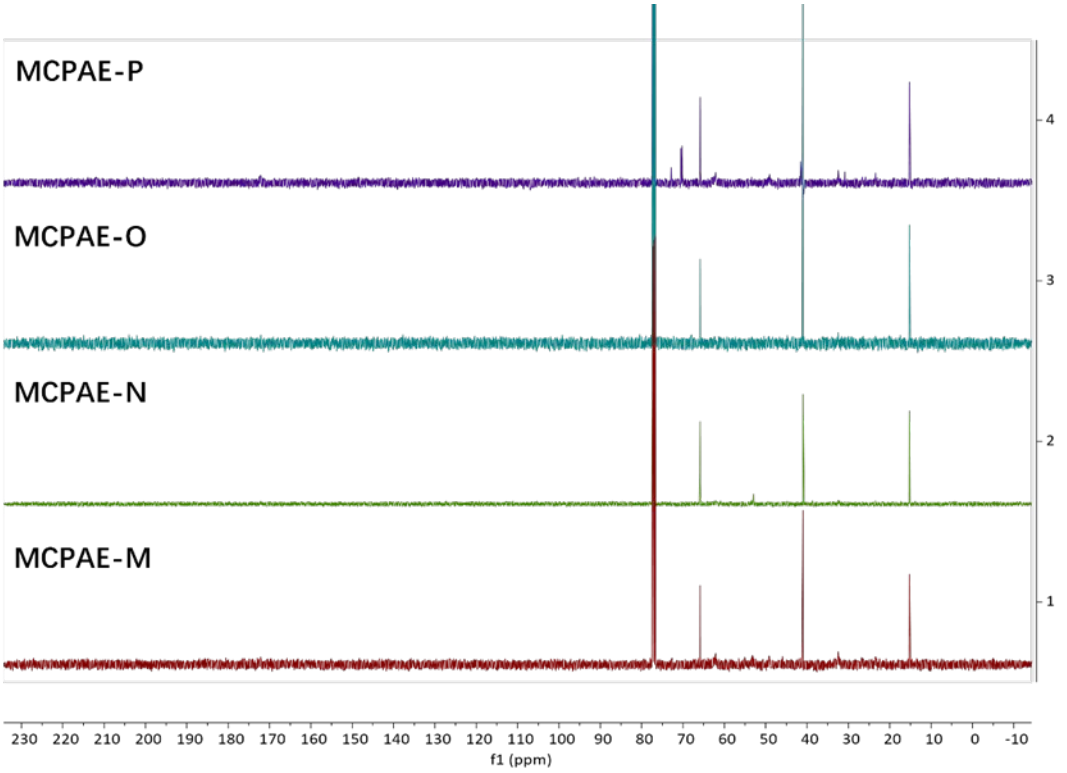

Figure S51. ^{13}C NMR spectra of MCPAE-M to -P synthesized by Method 3.

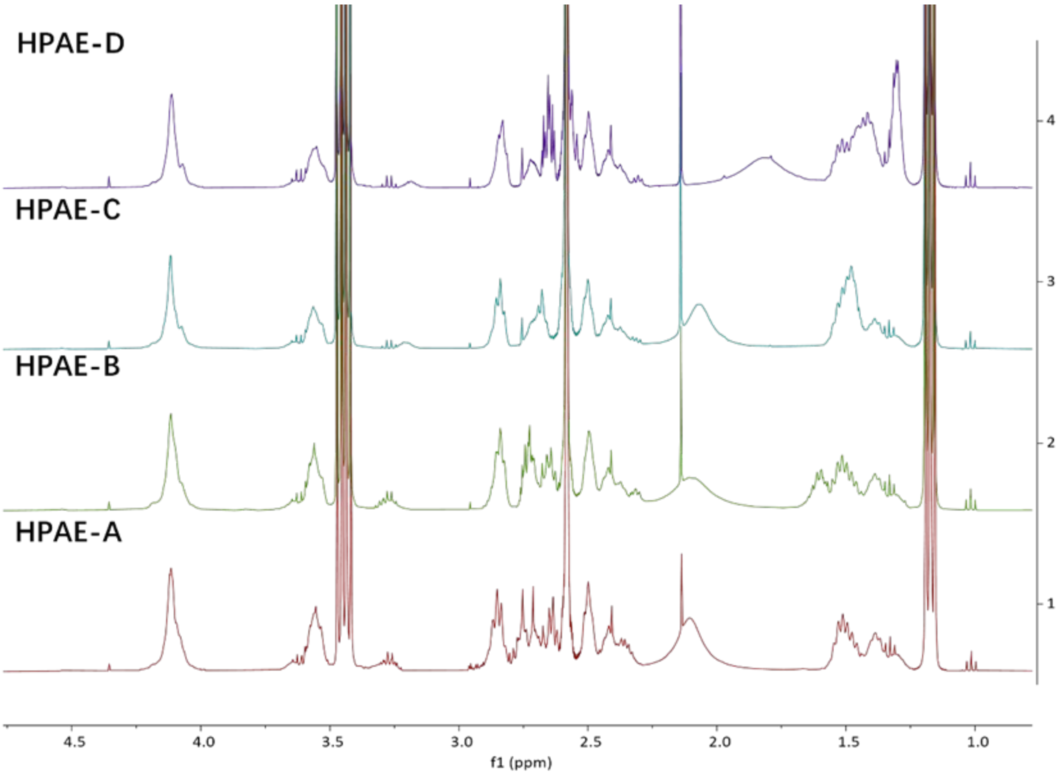

Figure S52. ^{1}H NMR spectra of HPAE-A to -D.

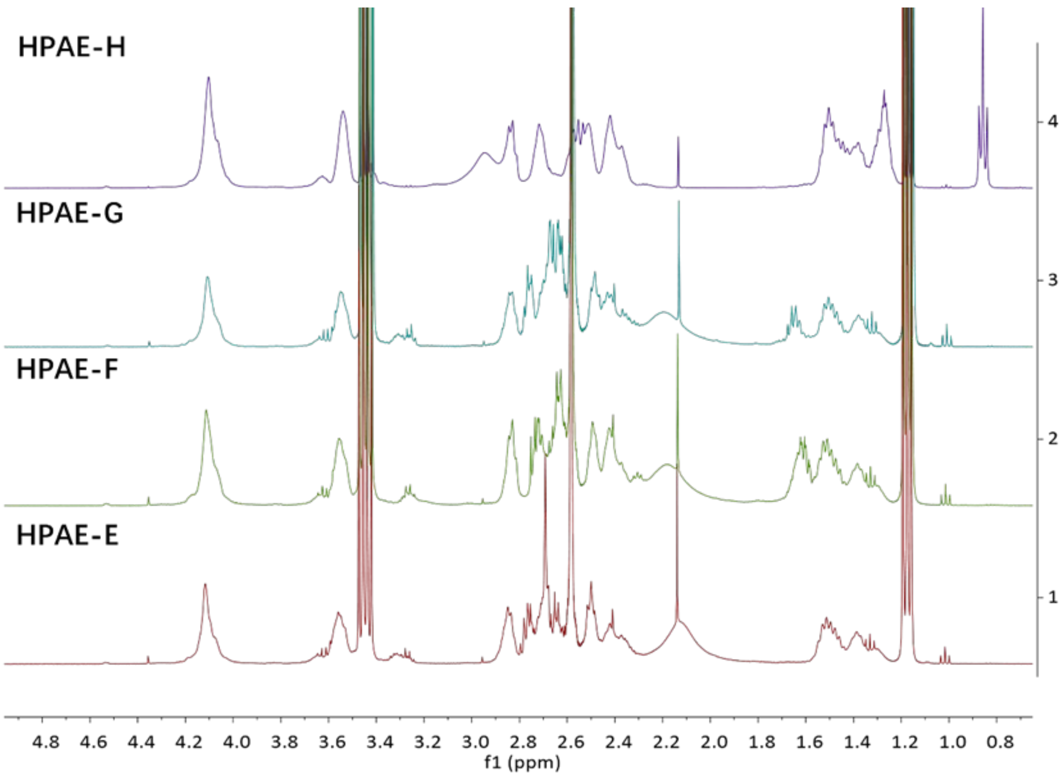

Figure S53. ^{1}H NMR spectra of HPAE-E to -H.

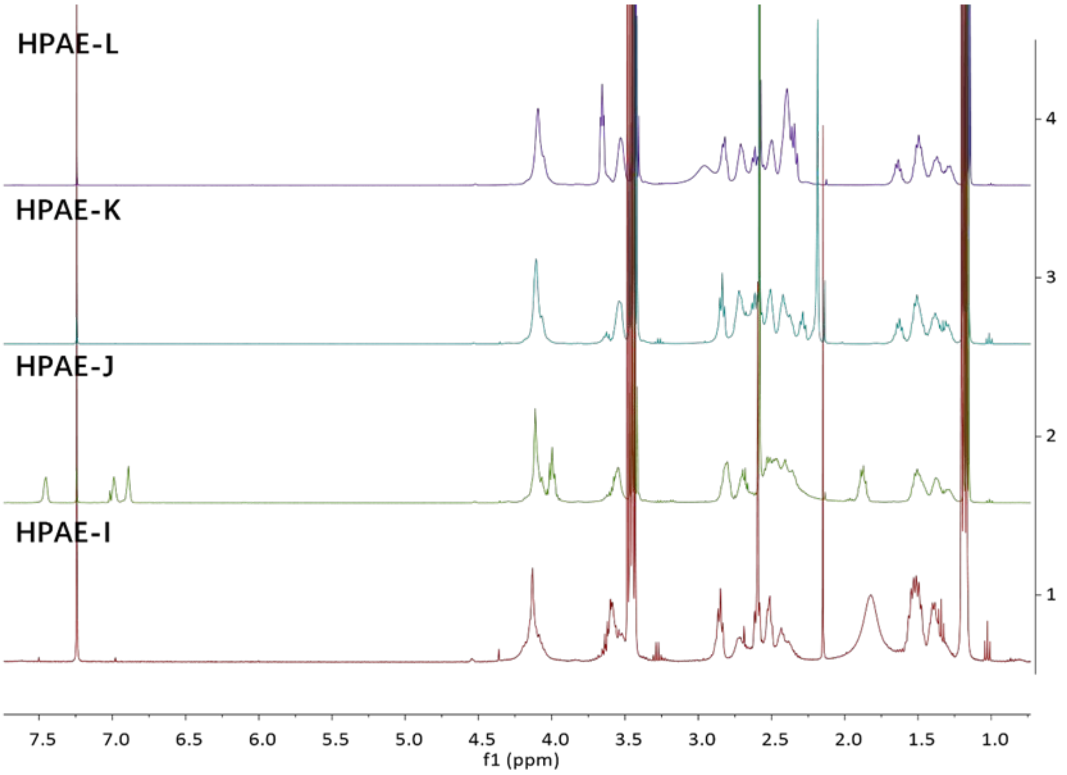

Figure S54. ^{1}H NMR spectra of HPAE-I to -L.

.

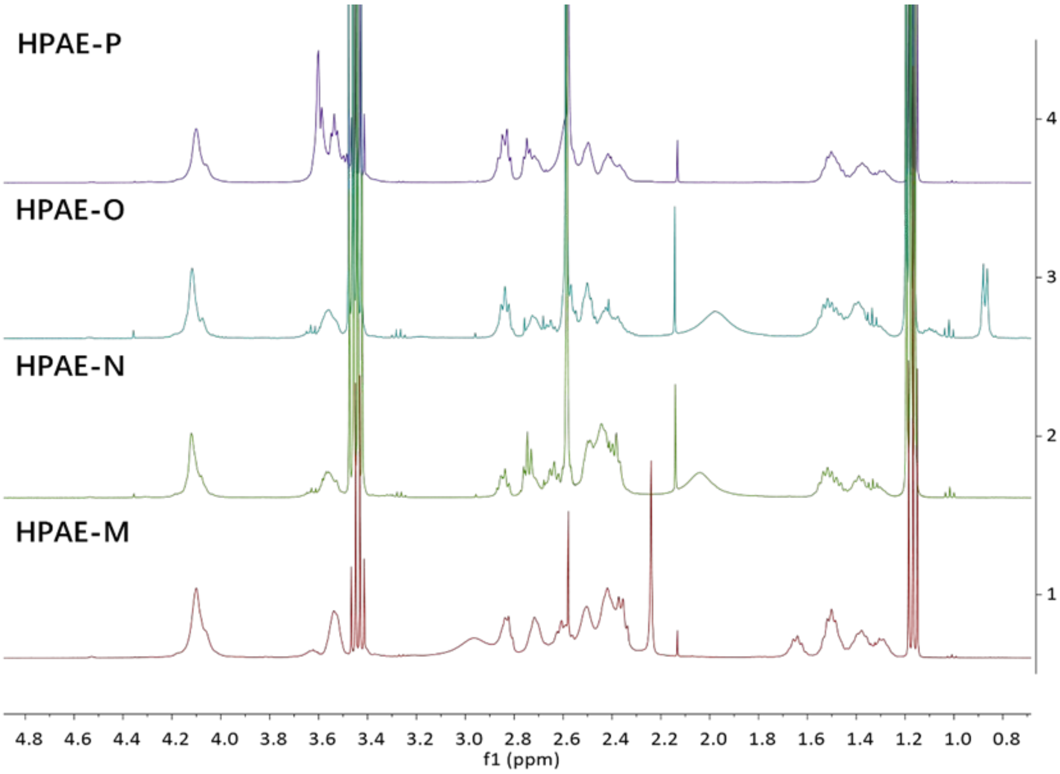

Figure S55. ^{1}H NMR spectra of HPAE-M to -P.

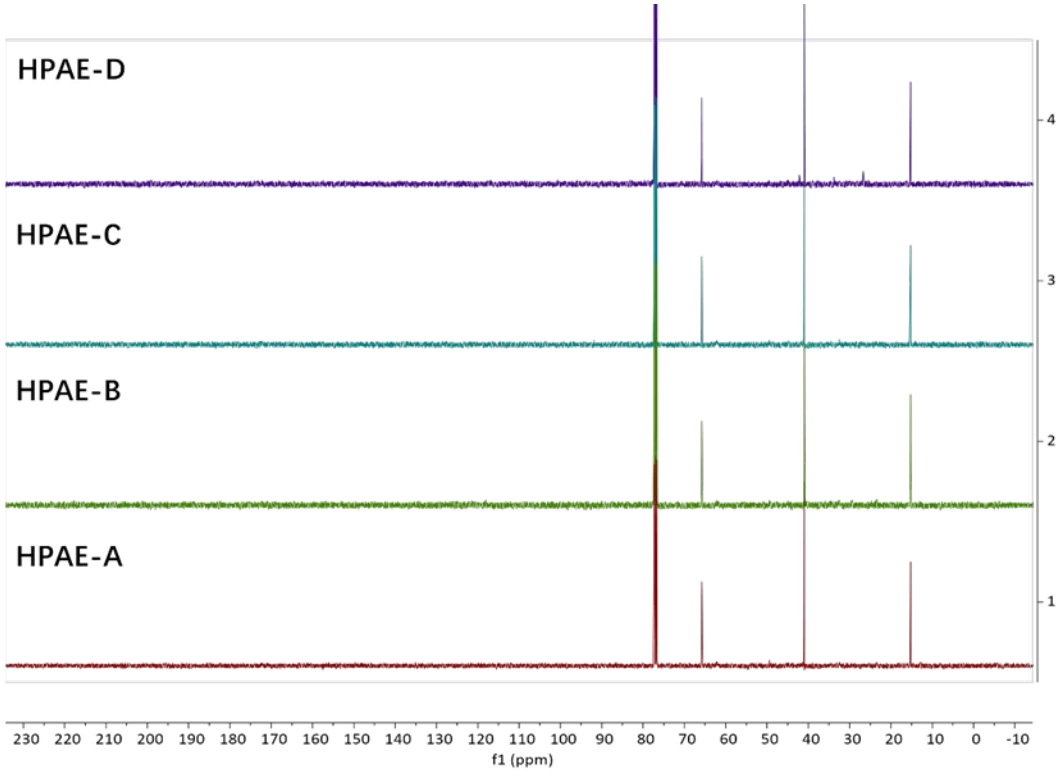

Figure S56. ^{13}C NMR spectra of HPAE-A to -D.

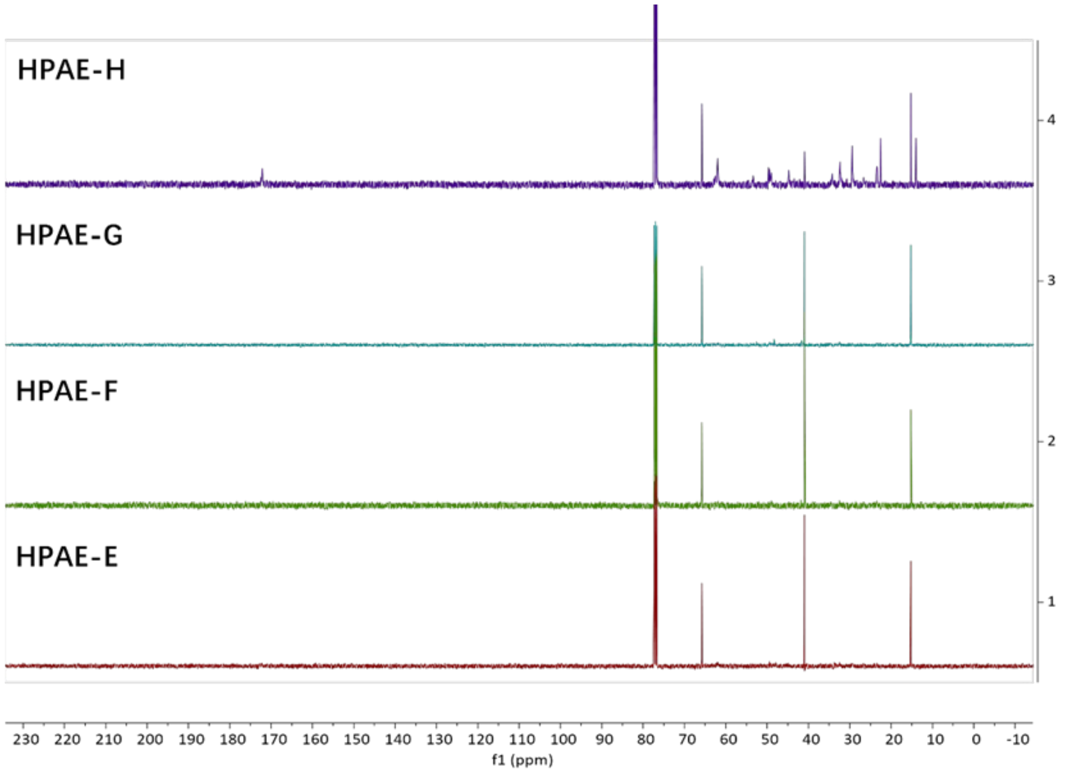

Figure S57. ^{13}C NMR spectra of HPAE-E to -H.

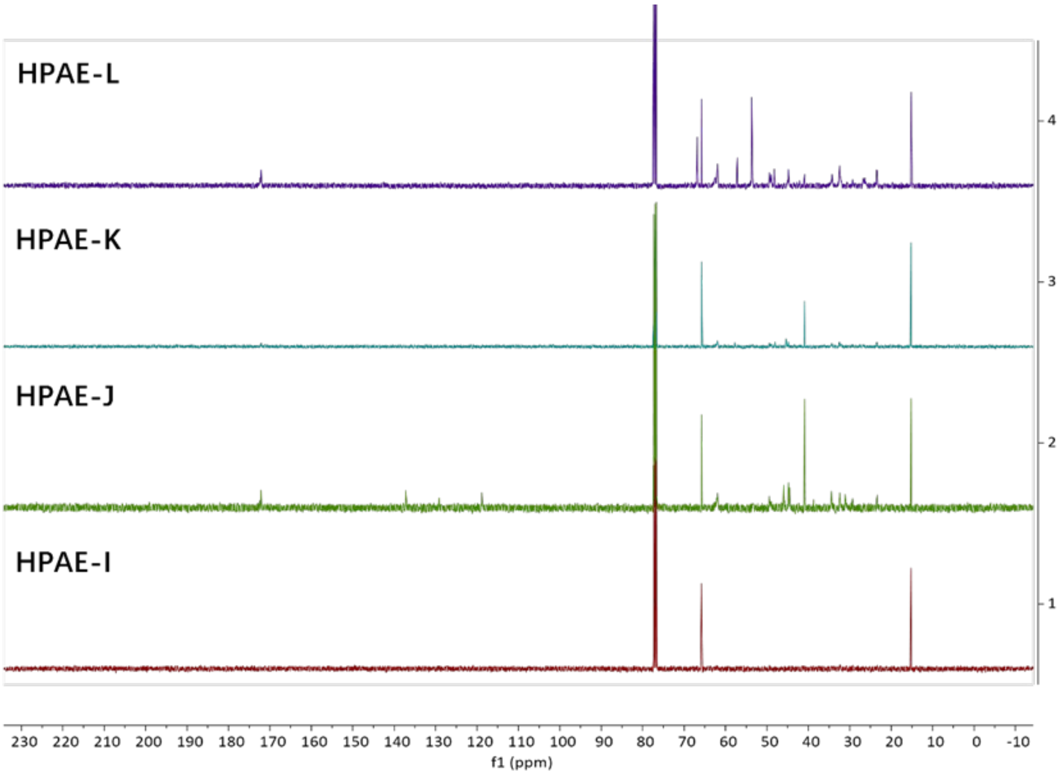

Figure S58. ^{13}C NMR spectra of HPAE-I to -L.

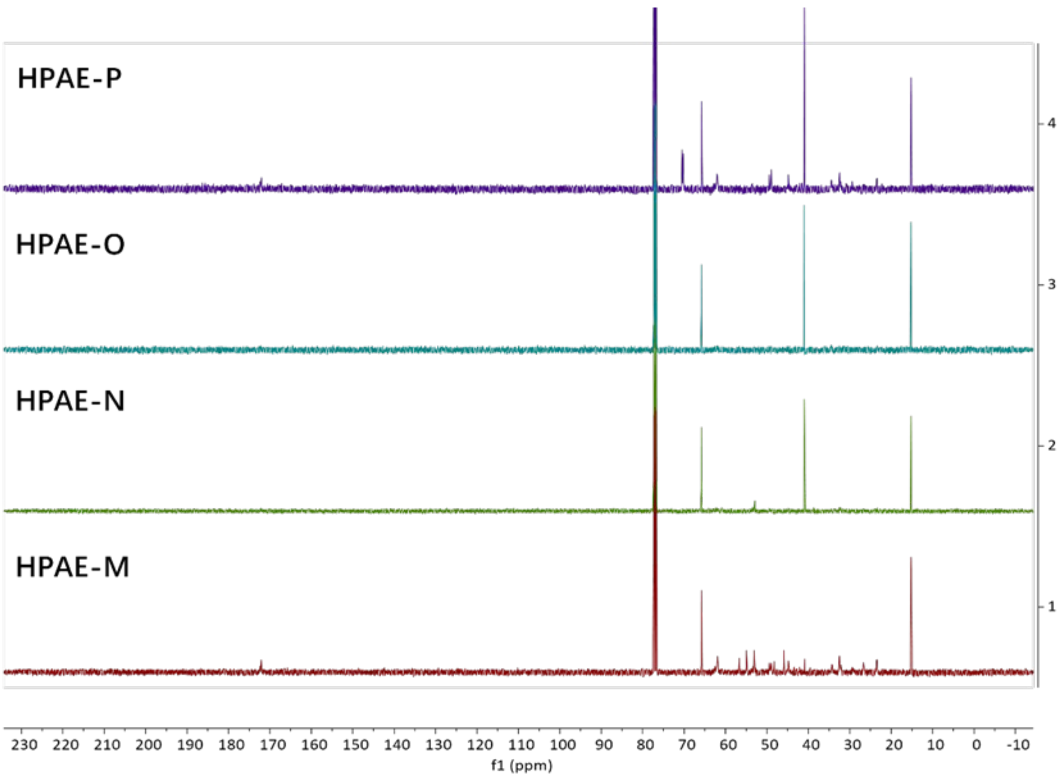

Figure S59. ^{13}C NMR spectra of HPAE-M to -P.

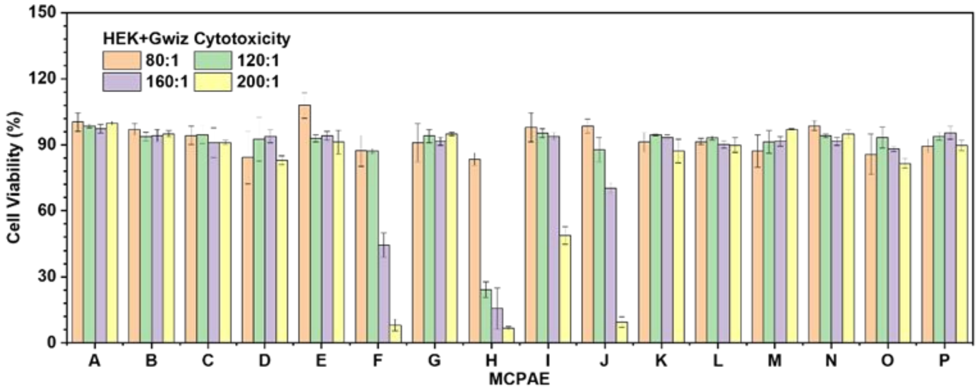

Figure S60. Cytotoxicity of MCPAEs with different terminal groups after 48 h transfection at different polymer:DNA ratios (w/w) of 80:1 to 200:1.

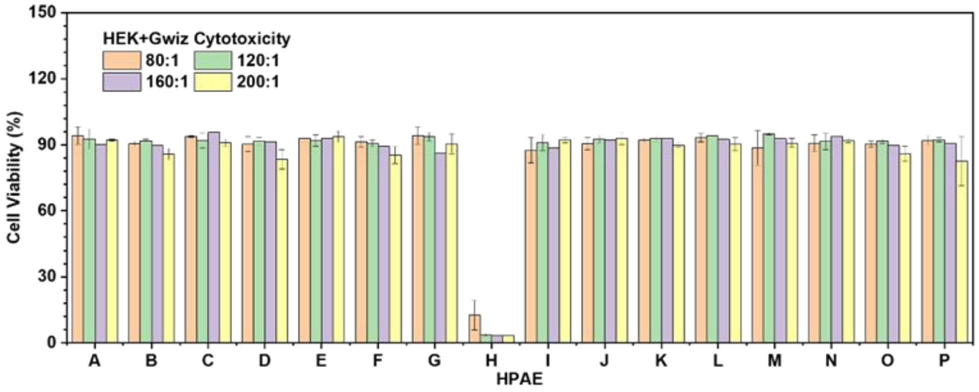

Figure S61. Cytotoxicity of HPAEs with different terminal groups after 48 h transfection at different polymer:DNA ratios (w/w) of 80:1 to 200:1.

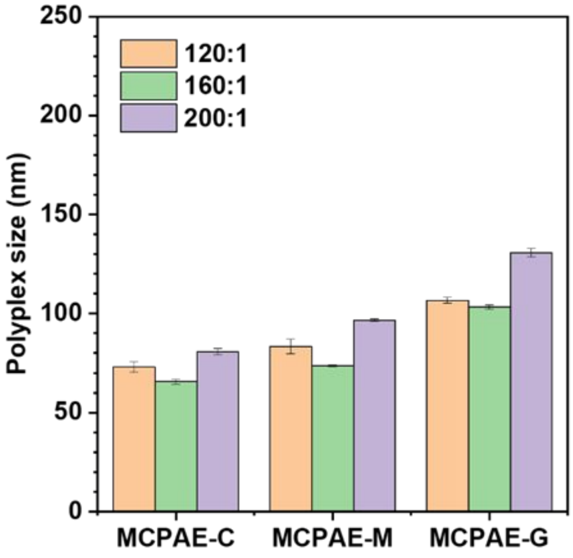

Figure S62. Polyplex size of MCPAE-C, -M and -G in 25 mM sodium acetate buffer at polymer:DNA ratios (w/w) of 120:1 to 200:1.

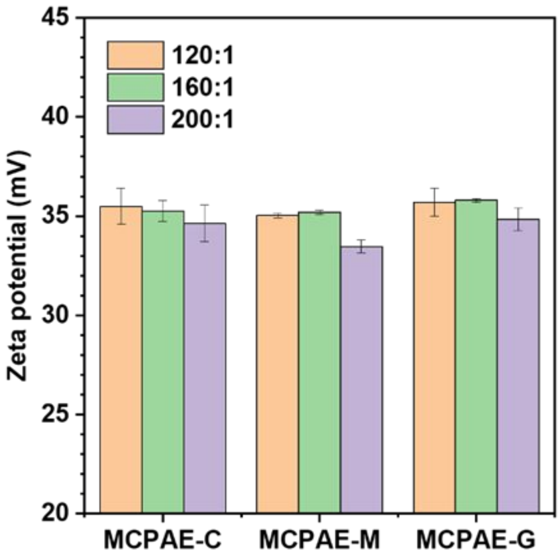

Figure S63. Potential of MCPAE-C, -M and -G in 25 mM sodium acetate buffer at polymer:DNA ratios (w/w) of 120:1 to 200:1.

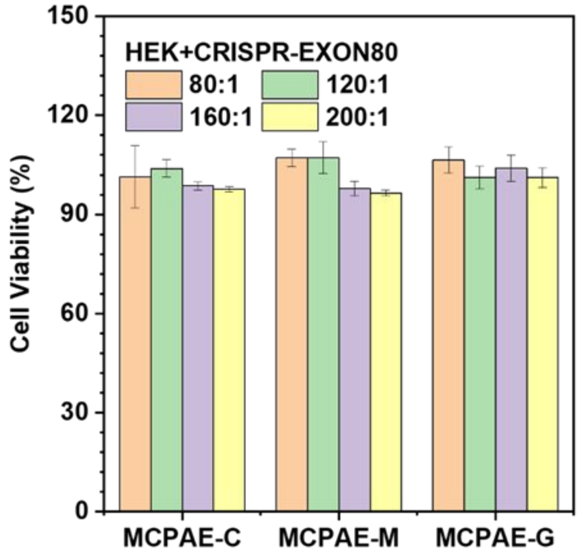

Figure S64. Cytotoxicity of MCPAE-C, -M, and -G with CRISPR-EXON80 plasmid in HEK cell line measured by alamarBlue assay at different polymer:DNA ratios (w/w) of 80:1 to 200:1.

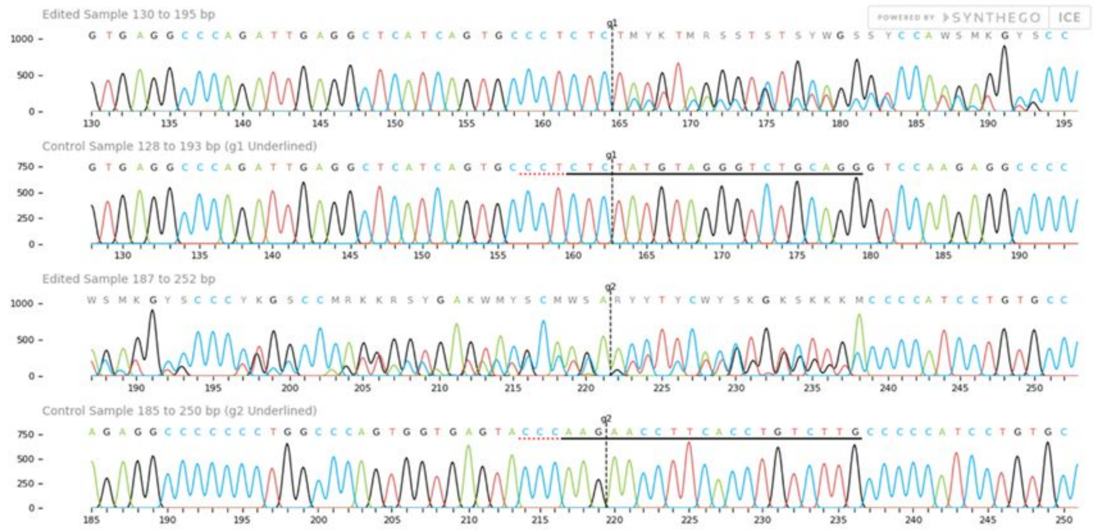

Figure S65. Trace of the edited gene on HEK cells post MCPAE-G with CRISPR-EXON80 plasmid treatment.

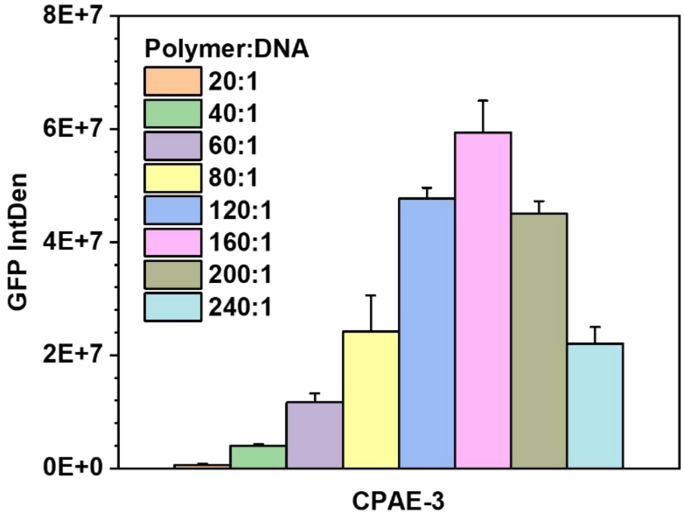

Figure S66. In vitro assessment of the gene transfection performance of CPAE-3 in HEK cells

Table S1. Polymer degradation study of CPAEs and HPAEs[c]

**Polymer**	**Degradation Time**	$M_{w,GPC}$ **(Da)**[a]	$M_{n,GPC}$ **(Da)**[b]	$Đ$
CPAE-1	0 hour	11651	4785	2.4
	1 hour	5556	1839	3.0
	2 hours	2603	1135	2.3
	3 hours	1932	892	2.2
	4 hours	1316	773	1.7
	8 hours	923	642	1.4
CPAE-2	0 hour	11609	4697	2.5
	1 hour	5330	1852	2.9
	2 hours	3277	1272	2.6
	3 hours	2300	1048	2.2
	4 hours	1995	950	2.1
	8 hours	1442	802	1.8
CPAE-3	0 hour	14244	4826	3.0
	1 hour	12011	2811	4.3
	2 hours	8277	1934	4.3
	3 hours	6048	1522	4.0
	4 hours	4701	1314	3.6
	8 hours	2419	944	2.6
HPAE-1	0 hour	10854	4663	2.3
	1 hour	2633	1271	2.1

	2 hours	1545	875	1.8
	3 hours	1179	741	1.6
	4 hours	985	672	1.5
	8 hours	800	604	1.3

[a]$M_{w,GPC}$: weight average molecular weight distribution measured by GPC; [b]$M_{n,GPC}$: number average molecular weight distribution measured by GPC.

Table S2. GPC and ^{1}H NMR characterization results of MCPAEs synthesized by Methods 3

**Entry**	**Polymer**	$M_{w,GPC}$ **(Da)**[a]	**Terminal ratio (TR)**[b]
1	MCPAE-0h	13257	1.315
2	MCPAE-6h	18305	1.151
3	MCPAE-12h	22064	1.034
4	MCPAE-19h	25669	0.918
5	MCPAE-31h	26911	0.883
6	MCPAE-72h	31067	0.772

[a]$M_{w,GPC}$: weight average molecular weight distribution measured by GPC; [b]TR: terminal ratio, calculated from the molar ratio of E7/PTTA according to ^{1}H NMR.

Table S3. GPC characterization results of MCPAEs with different terminal groups synthesized by Methods 3.

**Entry**	**Polymer**	$M_{w,GPC}$ **(Da)**[a]	$M_{n,GPC}$ **(Da)**[b]	**Đ**
1	MCPAE-A	11592	2934	3.9
2	MCPAE-B	11832	2958	3.9
3	MCPAE-C	19402	3769	5.1
4	MCPAE-D	22443	4517	4.9
5	MCPAE-E	16158	3222	5.0
6	MCPAE-F	17875	3305	5.4
7	MCPAE-G	17183	3233	5.3
8	MCPAE-H	14929	5309	2.8
9	MCPAE-I	14522	3726	3.9
10	MCPAE-J	14091	2777	5.1
11	MCPAE-K	11596	4441	2.6
12	MCPAE-L	14569	4734	3.1
13	MCPAE-M	13257	4905	2.7
14	MCPAE-N	15964	4402	3.6
15	MCPAE-O	22649	5021	4.5
16	MCPAE-P	25900	4898	5.2

[a]$M_{w,GPC}$: weight average molecular weight distribution measured by GPC; [b]$M_{n,GPC}$: number average molecular weight distribution measured by GPC.

Table S4. GPC characterization results of HPAEs with different terminal groups.

**Entry**	**Polymer**	$M_{w,GPC}$ **(Da)**[a]	$M_{n,GPC}$ **(Da)**[b]	$Đ$
1	HPAE-A	28020	4199	6.6
2	HPAE-B	22175	3561	6.2
3	HPAE-C	38247	4792	7.9
4	HPAE-D	41369	5416	7.6
5	HPAE-E	41556	4660	8.9
6	HPAE-F	38446	4344	8.8
7	HPAE-G	50622	4406	11.4
8	HPAE-H	30677	6459	4.7
9	HPAE-I	33751	5146	6.5
10	HPAE-J	30501	4387	6.9
11	HPAE-K	29920	5101	5.8
12	HPAE-L	29304	5763	5.1
13	HPAE-M	26827	5549	4.8
14	HPAE-N	38511	5402	7.1
15	HPAE-O	39785	5768	6.8
16	HPAE-P	35989	5200	6.9

[a]$M_{w,GPC}$: weight average molecular weight distribution measured by GPC; [b]$M_{n,GPC}$: number average molecular weight distribution measured by GPC.
